# Wearables for Running Gait Analysis: A Systematic Review

**DOI:** 10.1007/s40279-022-01760-6

**Published:** 2022-10-15

**Authors:** Rachel Mason, Liam T. Pearson, Gillian Barry, Fraser Young, Oisin Lennon, Alan Godfrey, Samuel Stuart

**Affiliations:** 1 Department of Sport, Exercise and Rehabilitation, Faculty of Health and Life Sciences, Northumbria University, Newcastle upon Tyne, UK; 2 DANU Sports Ltd., Dublin, Ireland; 3 Department of Computer and Information Sciences, Northumbria University, Newcastle upon Tyne, UK; 4 Northumbria Healthcare NHS Foundation Trust, Newcastle upon Tyne, UK

## Abstract

**Background:**

Running gait assessment has traditionally been performed using subjective observation or expensive laboratory-based objective technologies, such as three-dimensional motion capture or force plates. However, recent developments in wearable devices allow for continuous monitoring and analysis of running mechanics in any environment. Objective measurement of running gait is an important (clinical) tool for injury assessment and provides measures that can be used to enhance performance.

**Objectives:**

We aimed to systematically review the available literature investigating how wearable technology is being used for running gait analysis in adults.

**Methods:**

A systematic search of the literature was conducted in the following scientific databases: PubMed, Scopus, Web of Science and SPORTDiscus. Information was extracted from each included article regarding the type of study, participants, protocol, wearable device(s), main outcomes/measures, analysis and key findings.

**Results:**

A total of 131 articles were reviewed: 56 investigated the validity of wearable technology, 22 examined the reliability and 77 focused on applied use. Most studies used inertial measurement units (*n* = 62) [i.e. a combination of accelerometers, gyroscopes and magnetometers in a single unit] or solely accelerometers (*n* = 40), with one using gyroscopes alone and 31 using pressure sensors. On average, studies used one wearable device to examine running gait. Wearable locations were distributed among the shank, shoe and waist. The mean number of participants was 26 (± 27), with an average age of 28.3 (± 7.0) years. Most studies took place indoors (*n* = 93), using a treadmill (*n* = 62), with the main aims seeking to identify running gait outcomes or investigate the effects of injury, fatigue, intrinsic factors (e.g. age, sex, morphology) or footwear on running gait outcomes. Generally, wearables were found to be valid and reliable tools for assessing running gait compared to reference standards.

**Conclusions:**

This comprehensive review highlighted that most studies that have examined running gait using wearable sensors have done so with young adult recreational runners, using one inertial measurement unit sensor, with participants running on a treadmill and reporting outcomes of ground contact time, stride length, stride frequency and tibial acceleration. Future studies are required to obtain consensus regarding terminology, protocols for testing validity and the reliability of devices and suitability of gait outcomes.

**Clinical Trial Registration:**

CRD42021235527.

**Supplementary Information:**

The online version contains supplementary material available at 10.1007/s40279-022-01760-6.

## Key Points

**Table:** 

The majority of studies tested young adult recreational runners, with an average sample size of *n* < 30.
Most studies used one wearable (on shoe or tibia), typically an inertial measurement unit with a sampling rate of 100 Hz, with ground contact time, stride length, stride frequency and tibial acceleration outcomes most reported.
Most studies tested participants indoors, using a treadmill for a set duration or distance at a controlled speed.

## Introduction

Running is one of the most popular sport and recreational activities worldwide as well as being a core component of many sports [1]. In addition to its beneficial effects on health, the prevalence and cumulative incidence proportions of running-related injuries (RRI) are high, which results in participation cessation [2]. It is well established that a contributing factor to RRI is abnormal running gait, meaning early detection of potentially harmful running gait pathologies is essential. Where biomechanics have been implicated, clinical running analysis has largely been limited to the use of subjective clinical observation or rating scales (e.g. the High-Level Mobility and Assessment tool), which may not be sensitive to subtle changes in performance with training or injury [3–5].

Quantitative running gait analysis, as a clinical tool for minimising injury risk and as a performance measure, has been well documented in the literature [6–8]. However, quantification of running beyond clinical observation has largely been performed using a two-dimensional video analysis [3, 5] (particularly in commercial settings, such as running shoe stores), but this is limited to certain gait outcomes (i.e. foot strike patterns [FSP]) and still requires subjective visual/manual inspection and analysis of videos. To analyse more advanced measures, such as spatiotemporal (e.g. stride length [SL], stride time, step frequency [SF], speed), kinematic (e.g. angular velocity and joint angles) and kinetic (e.g. ground reaction forces [GRF]) measures, more cumbersome and expensive traditional (reference/gold-standard) gait laboratory measures are required (e.g. three-dimensional [3D] motion capture, force plate equipment, instrumented treadmills). However, use of gait laboratories for running gait assessment is limited because of the expense of equipment, the need for trained practitioners to collect and analyse data, and the requirement to attend a laboratory setting. Therefore, those traditional techniques are not readily available to performance or clinical settings and provide a limited understanding of running in ‘real-world’ environments [9–11]. Furthermore, laboratory-based testing often uses constrained protocols that may not represent usual running behaviour, such as assessing single foot strikes, unnatural force platform targeting and limited numbers of consecutive steps [12]. Numerous studies have sought to overcome this issue by using instrumented treadmills; however, further studies demonstrate the inconsistencies in running gait between over-ground and treadmill running [13]. In order to enhance understanding of running gait, further research in a natural running environment is required [12].

Wearable technology offers an alternative to overcome traditional assessment limitations and is becoming increasingly accepted by runners, coaches and clinicians [14]. Wearables utilising accelerometers, gyroscopes and magnetometers, applied individually or in combination as an inertial measurement unit (IMU), and ‘pressure-sensitive’ insoles allow quantification of a combination of spatiotemporal, kinetic and kinematic variables and have become a viable alternative owing to their portability and affordability [15]. Evidently, wearable devices can quantify various running gait outcomes in any setting (i.e. laboratory or outdoor/real world), which may enhance understanding of running performance, fatigue and injury mechanisms. Although research in this area is emerging, there have been some interesting developments. For example, previous studies have only been able to assess discrete timepoints (‘snap-shots’) throughout a run because of the use of force platforms and video analysis [16–18], whereas with recent improvements in accuracy, sensitivity and computing power, wearables have the potential to be an effective tool to measure the effects of fatigue on running biomechanics in the field, capturing the full duration of a run [19, 20].

Studies have also explored the use of wearable technology to quantify running gait patterns [19–21]. Within those studies, a wide range of protocols have been used indicating a lack of standardised methodology, and it is unclear whether the various wearables are valid or reliable for running gait assessment, which limits running gait interpretation. Coaches, researchers, clinicians or athletes who want to conduct similar running gait assessments or research are left with a choice of numerous protocols, which differ in many aspects. In the process of developing robust protocols, it is often helpful to have evidence-based recommendations. Therefore, the purpose of this review is to provide a comprehensive overview of studies that have used wearable technology for a running gait analysis, in order to provide some guidance regarding the selection of appropriate methodologies. We focused the review on the following: (1) methodologies employed to assess the validity and reliability of wearables for running gait assessment; (2) the application of wearables to assess running gait (i.e. aims, participants, environment, sensor type/location, protocol); (3) commonly reported running gait outcomes and findings; and (4) recommendations for future protocols and research. For the purposes of this review, when reporting our findings, we first provide a comprehensive description of all reviewed studies and then group the reviewed articles into two areas: (A) those that purely examined the *validity and reliability* of wearables for running gait assessment and (B) *application* of wearable sensors to assess running gait in different populations to inform performance or clinical outcomes.

## Methods

The protocol was prospectively registered on the PROSPERO International Prospective Register for Systematic Reviews website (registration no. CRD42021235527) in February 2021. Design and reporting of this review have followed the Preferred Reporting Items for Systematic Reviews and Meta-Analyses (PRISMA) 2020 statement [22].

### Search Strategy and Study Selection Process

**Fig. 1:**
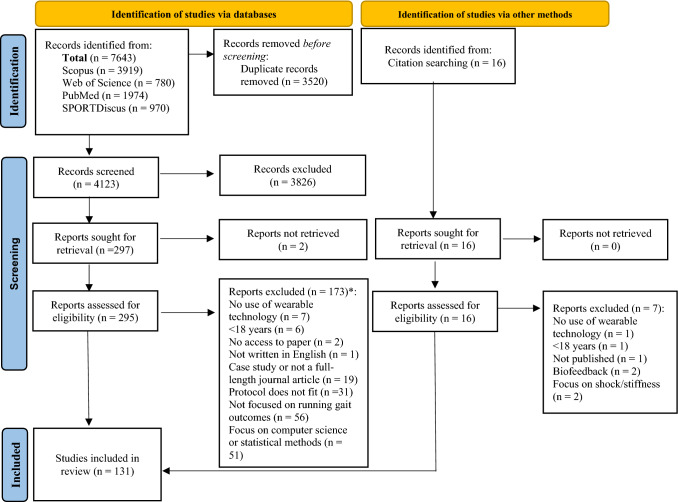
Preferred Reporting Items for Systematic Reviews and Meta-Analyses (PRISMA) 2020 flow diagram. *An in-depth list of excluded articles and the reasons can be found in Table 1 of the ESM

A systematic search was conducted to identify potentially relevant papers in the following scientific databases: PubMed, Scopus, Web of Science and SPORTDiscus. The focus of this review was on journal articles published in English that described the use of wearable technology to assess natural running gait in adults. The general search strategy and search terms are described in Table 1. Articles published up to 4 May, 2022 were reviewed. Thereafter, the article selection process consisted of the following steps using the PRISMA guidelines (Fig. 1): (1) an initial title screen for relevant articles was performed by independent authors (SS, RM), once the searched database results had been combined and duplicates had been removed; (2) both the titles and abstracts of the selected articles were reviewed (SS, RM) [a review of the full text was completed if it was not clear from the title or abstract whether the study met the review criteria]; and (3) the authors (SS, RM) read the full texts and selected articles based on the inclusion/exclusion criteria (Table 2). Additionally, the references of all included studies were checked for additional publications that could be included in this review. At all stages of the study selection process, decisions regarding inclusion or exclusion were made by two authors (SS and RM), with a third author (GB) consulted to resolve discrepancies (Table 1 of the Electronic Supplementary Material [ESM]).

**Table 1:** Systematic search strategy key terms

Wearable technology	“Wearable*” OR "Wearable Technology" OR "Wearable Devices" OR "Wearable Sensors" OR “IMU” OR “Inertial Sensor" OR "Inertial Measurement Unit" OR "Gyroscope" OR "Magnetometer" OR Acceleromet* OR "Force Plate" OR "Pressure Plate" OR "Pressure Sensor" TITLE-ABS-KEY
Running gait	“Running” OR “Jogging” OR “Run” OR “Jog” OR “Sprint” OR “Sprinting” OR “Sprints” OR “Runners” OR “Joggers” OR “Athletics” TITLE-ABS-KEY

* indicates a wildcard, that the search term can have any ending, TITLE-ABS-KEY indicates a title, abstract and keyword search

**Table 2:** Eligibility criteria

**Inclusion criteria** The articles contain a system for running gait analysis using wearable technology Sensing modality used was a wearable accelerometer, gyroscope, magnetometer or a combination of those (IMU), or pressure insoles Included at least one clearly defined running gait outcome measure, for example: Spatiotemporal (global outcomes of the running gait cycle): e.g. running velocity, acceleration of centre of mass, distance, ground contact time, step length, step frequency (cadence), stance time and flight time Kinematics (description of segmental or joint movement, generally in the three cardinal planes: sagittal, coronal [frontal], transverse planes, without consideration for forces): e.g. ankle dorsiflexion angle, ankle angular velocity or ankle angular acceleration Kinetic (the action of forces in producing or changing motion): e.g. GRF, peak pressure, centre of pressure, braking, impulse, time to peak pressure, pressure time integral, loads, force time integral, contact area and peak tibial acceleration Articles were written in the English language
**Exclusion criteria** Book chapters, review papers, case studies (i.e. a study examining one individual), letters, short communications, technical notes, conference proceedings and other non-peer-reviewed literature Studies evaluating the use of wearable technology for determination of step counts, distance, level of physical activity, classification or recognition of types of physical activity Studies focusing on the estimation of physiological measures (e.g. metabolic equivalents), maximal oxygen consumption, examination of external or neuromuscular load, stiffness, vibration and shock absorption of lower limbs Studies aiming to determine running power, stability or economy Studies investigating walking gait variability or regularity Studies not evaluating straight running (e.g. change in direction tasks or cutting manoeuvres) Studies investigating the use of biofeedback or gait retraining (i.e. non-natural running gait) Studies involving use of altered weight conditions (e.g. wearable resistance, anti-gravity treadmills or water-based protocols) Aims to evaluate only computer algorithms, machine learning or statistical approaches Studies evaluating robotic systems, exoskeletons, prosthetics, virtual reality environments and simulated data or models Study involves participants < 8 years of age Study concerns non-human animal subjects

*GRF* ground reaction force, *IMU* inertial measurement unit

### Data Extraction

Data were extracted by the author (RM) using a custom form to support standardised extraction (Appendix 1). Data were synthesised into a table format by the author (RM) and a second author (SS) confirmed data entry. Studies were divided into two categories based on the aims of this review: validity and reliability and application. Information extracted from each article included participants, sensor(s), study protocol, reference/additional measure, analysis, outcome measures and key findings.

## Results

### Search Results

**Fig. 2:**
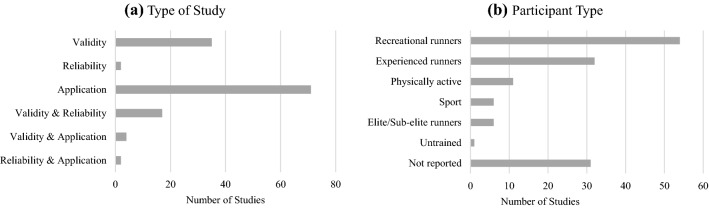
Summary of types of studies (**a**) and participant type (**b**) included in the review

From the 7643 articles identified through the database search, 122 papers met the inclusion criteria. An additional nine articles were identified through a search of reference lists. The complete flow diagram of the screening procedure is shown in Fig. 1. A total of 131 articles were reviewed, with overlapping reports on several topics; specifically, 56 examined validity, 22 examined reliability and 77 investigated the application of wearable technology for a running gait analysis (Fig. 2). Table 2 of the ESM provides key details about each article.

### Participant Characteristics

Overall, studies included between three [19] and 187 [23] participants, with the average number of participants being 26 (± 27). The mean age of participants was 28.3 (± 7.0) years. Two studies did not provide any age-related details [21, 24], with three studies providing age ranges only [25–27]. Three studies investigated running gait in participants with an average age over 50 years, none of which performed a comparison of gait patterns across age groups that included older adults [28–30]. Most of the reviewed studies (*n* = 82) included both male and female participants, with eight examining differences between male and female participants [31–38] and three of these studies finding significant differences between sexes [32, 36, 37]. Thirty-nine studies had male participants only, while only three studies solely examined female participants [39–41], seven studies did not report the sex of participants [21, 42–47] and two studies did not provide a breakdown of the sexes [26, 48]. The primary group of interest was healthy young adults who were recreationally active (Fig. 2), with only six studies investigating injured runners [39, 48–52] (Table 2 of the ESM). Twenty-four studies commented on the FSP of the participants: 18 of these investigated rear-foot strikers [34, 43, 52–70], one study examined rear-foot strikers or neutral FSP [71], and five studies compared running gait parameters between FSPs [23, 40, 52, 68, 69].

### Wearable Instrumentation

#### Inertial Measurement Units

Sixty-two articles stated that they used IMUs; however, 14 of these studies only used the accelerometer capabilities within the IMU [23, 24, 38, 52, 56, 57, 62, 72–78] and 20 studies stated they used the accelerometer and gyroscope components for the data analysis [26, 33, 43, 49, 50, 54, 59, 61, 79–90]. The remaining 27 studies either did not comment on components used [70, 91–98] or implied they used all accelerometer, gyroscope and magnetometer components for the data analysis [19–21, 28, 29, 36, 42, 99–109]. One study used an IMU and a separate one-dimensional accelerometer [61]. One study solely used the gyroscope housed within the IMU, using a sampling frequency of 102.4 Hz and not commenting on the gyroscope range [27]. Across these studies, the most common sampling frequency was 100 Hz (*n* = 12) [19, 21, 28, 29, 38, 42, 73, 75, 76, 78, 82, 94], but included use of 10 Hz [36] and 2000 Hz [43], and the range of the accelerometers was between ± 2.0 g [36] and ± 200 g [56, 57], with 16 g being the most frequently used (*n* = 14) [24, 26, 36, 59, 83, 85–89, 99, 100, 102, 103]. The gyroscope ranges (± °/s) used were 1200 (*n* = 7) [19, 20, 59, 86–89], 2000 (*n* = 10) [21, 26, 42, 61, 83, 85, 92, 100, 102, 103], 4000 [43] and a variety (*n* = 1) [36]. A variety of sampling frequencies (4–1000 Hz), accelerometer (2, 4, 6, 8, 16 g) and gyroscope ranges (250, 500, 1000, 2000°/s) were used in one study that used an IMU [36]. Twenty-eight studies reported the weight and/or size of the IMU used, with a large range. IMUs were as small as 6.0 × 1.85 × 0.5 cm [83] up to 8.8 × 5.0 × 1.9 cm [38], and the weight of the IMUs ranged from 4 g [43] to 550 g [92] (Table 2 of the ESM).

#### Accelerometers Only

Of the 40 studies that stated they used single accelerometer configurations in their methodology, notably, 13 studies did not comment on the dimensions [31, 41, 51, 58, 60, 63, 64, 110–115], one-dimensional accelerometers were used exclusively in four studies [44, 48, 61, 116], one study featured a two-dimensional accelerometer [53], one study used both one-dimensional and 3D accelerometers [117], and 21 studies used 3D accelerometers only (Table 2 of the ESM). Where reported, sampling frequency was between 30 Hz [117] and 1667 Hz [111], with 1000 Hz being the most common (*n* = 14) and the range of the accelerometers was between ± 0.05–2.0 g [117] and ± 50 g [118], with 16 g being the most frequently used (*n* = 8). There was a large range in reported sizes of accelerometers from as small as 4.0 × 2.2 × 1.2 cm [119] up to 5.42 × 10.25 × 1.7 cm [120], weighing between 2.5 g [119] and 67 g [121] (Table 3 and Table 2 of the ESM).

**Table 3:** Type of sensor used within reviewed studies

Type of wearable technology used	*n*	References
IMU (a combination of sensors in one unit; accelerometer, gyroscope, magnetometer)	61	[19–21, 23, 24, 26–29, 33, 36, 38, 42, 43, 49, 50, 52, 54, 56, 57, 59, 62, 70, 72–97, 99–109, 128]
Accelerometer only	37	[30–32, 34, 35, 41, 44, 46, 48, 51, 58, 60, 63, 64, 110–121, 129–139]
Pressure sensor/insole	27	[25, 37, 39, 40, 45, 47, 55, 65–69, 71, 124, 125, 140–151]
Pressure sensor and accelerometer	2	[53, 123]
Pressure sensor and IMU	2	[98, 122]
IMU and separate accelerometer	1	[61]
Gyroscope	1	[127]

Note: Table refers to how each study classified the technology used, rather than the components used for analysis (i.e. some studies used an IMU, but only analysed data from one element of the unit)

*IMU* inertial measurement unit

#### Pressure Sensors/Insoles

Of the 131 articles reviewed, 31 studies focused on pressure or force-sensitive insoles; two of those 31 studies investigated the use of a combined pressure insole and an IMU [98, 122] and a further two studies utilised a pressure insole alongside accelerometers [53, 123]. Of the studies that used pressure insoles, the lowest sampling frequency was 50 Hz [39, 124, 125] and the highest was 1029 Hz [123]; 100 Hz was the most common sampling frequency (*n* = 13). Seven studies commented on the dimensions of the insoles/sensors [25, 53, 65, 66, 71, 122, 126], with the dimension range from 0.6 × 0.4 × 0.12 cm [65] to 2.55 cm [66] (Table 3 and Table 2 of the ESM).

#### Gyroscope Only

One study solely used a gyroscope (not encompassed in an IMU), with a sampling frequency of 1500 Hz and a gyroscope range of 250°/s [127] (Table 3 and Table 2 of the ESM).

### Number of Sensors

In the reviewed studies that used IMUs, accelerometers or gyroscopes, most studies used one (*n* = 56) or two (*n* = 30) sensors. Few studies used more than two sensors, for example, others used three [77, 97], four [62, 74, 117], five [106], seven [103, 109], eight [19, 20, 85], nine [105], 12 [21] or 17 sensors [108]. Where studies used more than one sensor, they were not necessarily the same type of sensor (e.g. one IMU and one accelerometer). For example, two studies sought to compare multiple and single sensor units [93, 130]. Notably, of the studies that used multiple sensors, six sought to investigate the influence of sensor location on outcome measures [74, 85, 93, 101, 128, 130] (Table 2 of the ESM).

### Location

**Fig. 3:**
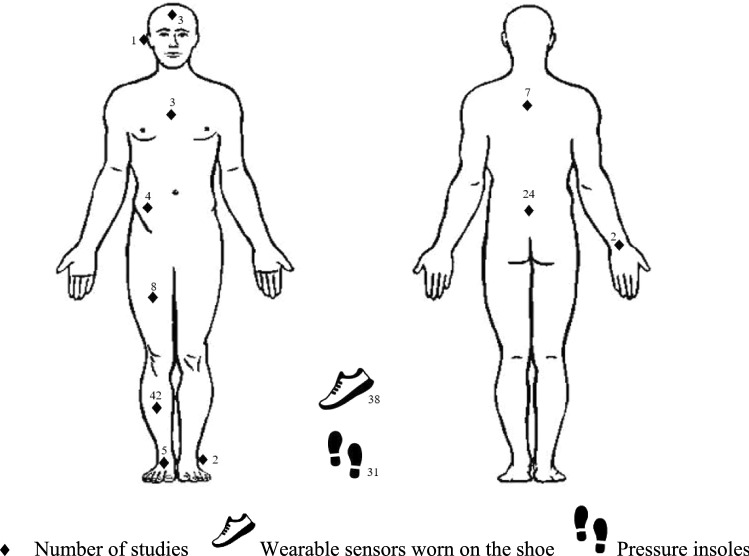
Frequency distribution of the body segments on which wearables were placed

The most common inertial wearable locations were the tibia (*n* = 42), mostly located at the distal anteromedial aspect; shoe (*n* = 38), varying locations of dorsal aspect/shoelaces/instep, cavity, ankle, heel and fifth metatarsal; and lower back [including sacrum] (*n* = 24). One study used instrumented earbuds [135], and a further four studies placed wearables on the sternum/chest and these were always in combination with a lower body sensor placement [19, 20, 89, 93]. In the seven studies that used wearables on the upper back, five studies placed the sensor in a harness/vest [21, 38, 105, 121, 129, 130, 133]. Two studies located accelerometers on the wrist, housed in GPS watches [21, 31] and one study mounted 17 sensors onto a lycra suit that participants wore [108] (Fig. 3 and Table 2 of the ESM).

### Extracted Features/Outcome Measures

Table 4 provides a full breakdown of reported outcome measures. Outcomes included spatiotemporal, kinematic and kinetic running gait parameters. Among the studies that investigated spatiotemporal parameters, measures of distance included SL (*n* = 29) and less commonly, vertical oscillation (*n* = 7), while ground contact time (GCT)/stance time (*n* = 49), SF (*n* = 36), and stride or step time (*n* = 16) were the most frequently reported temporal measures. Measures of acceleration included peak or average acceleration of a particular body segment, most commonly the tibia (*n* = 28). Where pressure insoles were used, plantar pressure (*n* = 17), contact area (*n* = 12) and pressure or force–time integral (*n* = 10) were the most reported outcomes.

**Table 4:** Outcome measures extracted from wearable technology within reviewed studies

Outcome	Definition	References
**Spatiotemporal**		
Ground contact time/stance time *n* = 49	The time between initial foot contact and toe-off for the same foot	[24, 27–29, 33, 37, 40, 42, 49–51, 53–55, 59, 63, 66, 67, 71, 75, 77, 79, 80, 85–87, 89, 90, 92, 95, 96, 99, 101, 105, 107, 110, 120, 123–125, 128, 134, 135, 139, 140, 143, 144, 150, 151]
Cadence, step/stride frequency/rate *n* = 36	The number of steps or strides taken during a given time	[20, 21, 28, 29, 31, 33–35, 51, 59, 70, 72, 77, 80, 82, 87, 90, 91, 93, 101, 102, 105, 106, 108, 110, 111, 117, 119, 120, 125, 128, 131, 132, 135, 139, 147]
Step/stride length *n* = 29	The distance between successive points of initial contact of the same foot (stride) or opposite foot (step)	[20, 33–35, 42, 49, 50, 54, 59, 61, 70, 80, 85, 87, 90–92, 98, 100–102, 105, 106, 108, 119, 128, 132, 139, 143]
Step/stride time *n* = 16	The time between two consecutive heel strikes of the same foot (stride) or opposite foot (step)	[27, 34, 42, 48, 59, 72, 75, 85, 90, 92, 100, 101, 106, 107, 128, 143, 144]
Foot strike pattern/strike index/foot strike angle *n* = 15	The moment, way or angle when the foot first makes contact with the ground	[21, 26, 33, 40, 59, 68, 69, 80, 84, 87–89, 93, 116, 144]
Flight time *n* = 15	The time between toe-off from one foot to initial contact of the other foot	[50, 77, 87, 89, 90, 92, 95, 96, 101, 105, 110, 124, 128, 139, 144]
Acceleration* *n* = 15	The rate of change of the velocity of any segment (excluding tibia)	[19, 35, 38, 62, 64, 73, 74, 78, 93, 97, 116, 119, 121, 129, 130]
Speed/velocity *n* = 15	The rate of change of position (directional)	[33, 42, 61, 79, 82, 85, 90–92, 98, 100, 102, 104, 106, 129]
Gait events *n* = 11	Identification of any of the key gait events, e.g. heel strike, toe-off, mid-stance	[27, 36, 59, 63, 71, 78, 92, 98, 130, 144, 150]
Vertical oscillation *n* = 9	The amount that the torso or COM moves vertically with each step or stride	[20, 21, 28, 29, 59, 90, 93, 134, 139]
Swing time *n* = 7	The period during which the foot is not in contact with the ground	[27, 39, 59, 89, 124, 125, 139]
Cycle time *n* = 4	The time taken to complete a single gait cycle (can be measured from any gait event to the same subsequent event on the same foot)	[46, 49, 54, 89]
Acceleration of centre of mass *n* = 4	The rate of change of the velocity of the centre of mass	[30, 32, 72, 76]
Step/stride height/foot clearance *n* = 2	The vertical distance obtained during the swing phase	[82, 98]
Symmetry *n* = 2	Any measure of imbalance between the right and left leg	[133, 137]
**Kinematic**		
Ankle/foot kinematics *n* = 19	Description of ankle or foot movement in any of the three cardinal planes: sagittal, frontal, transverse planes, without consideration for forces	[19, 20, 33, 43, 49, 50, 54, 73, 80, 85, 87, 89, 94, 103, 105, 107–109, 122]
Hip/pelvis kinematics *n* = 11	Description of hip or pelvis movement in any of the three cardinal planes: sagittal, frontal, transverse planes, without consideration for forces	[19, 20, 28, 29, 36, 73, 103, 105, 106, 108, 109]
Knee kinematics *n* = 10	Description of knee movement in any of the three cardinal planes: sagittal, frontal, transverse planes, without consideration for forces	[19–21, 73, 97, 103, 105, 106, 108, 109]
Trunk kinematics *n* = 3	Description of trunk movement in any of the three cardinal planes: sagittal, frontal, transverse planes, without consideration for forces	[21, 105, 108]
**Kinetic**		
Derivatives of tibial acceleration *n* = 28	Acceleration of the tibia, including peak positive acceleration, gradient, slope, magnitude, loading rate	[19, 34, 35, 41, 42, 44, 46, 48, 52, 53, 56–58, 60, 61, 64, 74, 111–115, 118, 119, 123, 131, 137, 138]
Plantar pressure *n* = 17	Pressure = force/area, where force describes the vGRF exerted and area describes the surface area of the foot that is in contact with the ground during running	[25, 37, 40, 45, 47, 53, 55, 65–69, 143, 145, 146, 148, 151]
Ground reaction force *n* = 13	The force exerted by the ground on a body in contact with it	[24, 59, 87, 95–97, 123, 130, 136, 139–142]
Contact area *n* = 12	Area of contact for each or any plantar region	[37, 47, 55, 65–69, 145, 146, 148, 151]
Pressure or force–time integral *n* = 10	The cumulative effect of pressure or force during a step cycle (area under the pressure–time or force–time curve)	[40, 47, 65, 66, 68, 69, 124, 125, 145, 150]
Impact *n* = 9	The vertical force observed during the initial contact	[23, 33, 49–51, 54, 80, 93, 147]
Force *n* = 8	Including rate and magnitude of force development	[39, 40, 47, 90, 104, 124, 149, 150]
Impulse *n* = 8	A measure of the force applied for a specific time and distance	[34, 45, 47, 123, 124, 140, 142, 149]
Braking *n* = 7	The force applied to the body that causes it to slow down	[28, 29, 33, 49, 50, 54, 93]
Load/loading rate *n* = 5	The speed at which you apply forces to the body	[45, 97, 123, 143, 149]
Centre of pressure *n* = 2	The point of application of the GRF	[125, 141]

Note: Some studies fall under more than one category

*GRF* ground reaction force, *vGRF* vertical ground reaction force

### Protocol

#### Environment

**Fig. 4:**
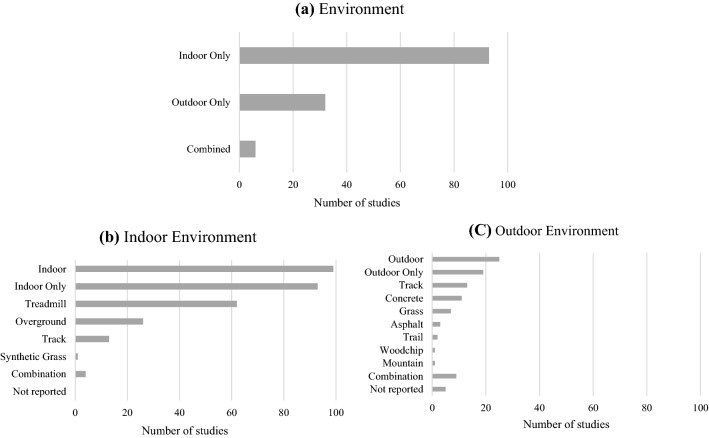
Summary of environments that studies were conducted within (**a**) overall, (**b**) indoor environments and (**c**) outdoor environments

Figure 4 provides an overview of the environments used for running assessments. Most studies (*n* = 93) used indoor facilities only that primarily involved treadmill running (*n* = 62). Thirty-two of the reviewed studies investigated running gait in outdoor environments only, and six studies used a combination of both indoor and outdoor testing [21, 53, 55, 60, 91, 143]. Eighteen studies examined running gait over more than one surface [21, 36, 46, 53–55, 60, 66, 67, 80, 87, 91, 105, 118, 125, 131, 143, 151]. The most popular outdoor surface was a running track (*n* = 16), followed by concrete (*n* = 13). Five studies did not report the outdoor surface type where testing took place [28, 33, 50, 59, 89] (Fig. 4 and Table 2 of the ESM).

#### Running Gait Protocol

##### Duration/Distance

The duration or distance of the analysed running protocol varied greatly by study. One hundred and nine studies analysed running gait in a single day, while 22 studies tested running gait over 2 or more days (Table 2 of the ESM). Protocols were heterogeneous and consisted of:

- *Analysing a certain number of steps, strides or gait cycles* (*n* = 50)*.* For example, four stages of 100 strides [20], three different footwear types, and five trials each, analysing one right foot strike per trial [115].
- *Analysing running gait for less or equal to 60 s* (*n* = 42). For example, one 15-s run [56, 74], and 30 trials lasting 30 s (five trials, six conditions, last 30 s of 3-min trials) [71].
- *Analysing running gait in trials lasting over 1 min and less than 5 min* (*n* = 17) [32, 36, 49, 72, 75, 80, 83, 92, 97, 98, 101, 120, 123, 128, 134]. For example, three sessions each consisting of three 5-min runs at varying speeds [75], seven 100-m runs (outdoor) and seven 60-s runs (treadmill) [91], or 3 min [36].
- *Analysing gait patterns over longer distances that were more representative of a typical run [i.e. more than 5 min]* (*n* = 22). For example, dissecting a 100-km (ultra-marathon) into ten 10-km segments to investigate the effects of fatigue [31], or analysing one 10-km segment and 15 2-km segments of a marathon race [29]. One study examined various distances; however, different participants were used for each distance [80].

##### Speed/Pace

There was variation in speed amongst the reviewed studies. Seventy-seven studies used controlled speeds (58 of these controlled at a set pace), with a range from 2 m/s [85, 121] to 26 km/h [117]. Nine studies controlled speed based on individual performance; four of these studies used personal bests as the benchmark [42, 48, 87, 108] and two studies controlled speed based on the participants’ preferred speed (e.g. 85 and 115% preferred speed) [21, 98]. The remaining three studies used physiological measures to determine speed used [19, 70, 111], for example, one study controlled running speed at 2 mmol/L blood lactate [70] (Table 2 of the ESM).

Fifty-five studies examined running gait at self-selected speeds; amongst these studies there were large variations in instructing speed. For example, six studies used race scenarios [20, 29, 31, 59, 82, 89], 14 studies asked participants to run based on perception (e.g. ‘easy run’/‘comfortable’, 75% maximum effort) [33, 44, 49, 54, 79, 99, 101, 105, 125, 128, 136, 141, 147, 148], and a further 11 studies instructed participants to run at maximum effort/speed [74, 86, 88, 90, 99, 100, 104, 105, 129, 132, 133].

Eight studies combined controlled and self-selected speeds [35, 62, 76, 116, 118, 127, 132, 135]. For example, Giandolini et al. examined participants at 10, 12, 14 (female) and 16 km/h (male), maximum aerobic speed and participant’s preferred speed [116]. Where speeds were reported, 46 studies included two or more speeds in their protocol.

##### Gradient

Sixteen studies commented on the running gradient [28, 34–36, 76, 80, 90, 101, 108, 109, 125, 128, 131, 144, 147, 149]. The majority of studies (*n* = 5) used a 0% gradient [34, 101, 109, 128, 144] or a 1% gradient (*n* = 4) on the treadmill [35, 36, 76, 125]. Three studies analysed the effects of different gradients [28, 80, 90], and one study investigated the effects of low and high altitudes on running gait [82].

##### Footwear

Forty-three studies required participants to wear standardised shod running shoes, of whom 42 utilised the participant’s own running shoes. Two studies tested participants in standardised footwear and in their own footwear [109, 116]. One study tested participants in socks as participants wore the insoles seeking validation wearing tight-fitted socks without shoes to allow a more direct measurement comparison [150]. Lucas-Cuevas et al. used standardised shoes and participants’ own insoles inside the participants’ own running shoes [119]. Forty-six studies did not comment on the footwear used (Table 2 of the ESM).

### Validity and Reliability Studies

Fifty-six studies focussed on the validation of wearables for running gait assessments, with 18 also examining the reliability of devices [47, 98, 99, 103, 104, 110, 117, 120, 130, 134, 136, 140, 144, 149]. Eleven studies investigated between-day reliability [34, 47, 98, 106, 117, 120, 122, 140, 142, 144, 149], and three studies solely examined the reliability of wearable technology [87, 134, 138] (Table 2 of the ESM).

#### Protocols for Validity and Reliability

##### Participants

Protocols to assess validity and reliability varied greatly. Overall, studies included between five [27] and 100 [95, 96] participants, with the average number of participants being 22 (± 18). The mean age of participants was 26.8 (± 4.5) years. Two studies only provided age ranges [26, 27] and one study did not report age [24]. Sixteen studies used male-only participants [61, 73, 84, 92, 94, 97, 98, 110, 115, 117, 121, 127, 129, 130, 133, 138], two did not report or provide the breakdown of sex [26, 45], and the remaining studies included both male and female participants. All studies included healthy participants and four studies commented on the FSP of the participants [34, 61–63].

##### Environmental Control

Six validity and/or reliability studies used outdoor environments, with participants running on concrete [79, 87], artificial turf [105] and track [102, 105, 120, 152]. Of the remaining studies that used indoor environments, 31 ran on treadmills, 15 ran over-ground [45, 63, 78, 85, 94, 104, 110, 115, 121, 124, 127, 130, 141, 142, 150] and six ran on a track [61, 99, 100, 129, 133, 136]. No studies used both indoor and outdoor testing or examined running gait over more than one surface. Seven studies commented on the treadmill gradient, one study set the treadmill at a 0, 10 incline and 10% decline [149], two studies used a 1% treadmill gradient [75, 76] and the remaining study stated that no gradient was used (i.e. 0%) [34, 101, 128, 144] (Table 2 of the ESM).

##### Distance/Time Control

Twenty-three studies focused on analysing a certain number of steps, strides or gait cycles, with the minimum being six foot strikes in total (three trials, two speeds) [127], and a maximum of 200 consecutive left and right steps of a 5-min run [140]. Thirty-three studies investigated running gait over particular distances or times whereby 23 studies analysed running gait for ≤ 60 s. Ten studies analysed running gait in trials lasting > 1 and < 5 min [36, 75, 92, 97, 98, 101, 106, 120, 123, 128]. One study examined gait patterns over a long distance, i.e. up to 4 km [79], and another study did not comment on the number of steps or distance analysed [94]. Within reliability studies, ten analysed test-re-test reliability in a single day (i.e. two sessions in 1 day) [98, 99, 103, 104, 106, 110, 130, 134–136] and 11 studies performed a test-re-test analysis on different days [34, 47, 87, 117, 120, 122, 138, 140, 142, 144, 149]. Those studies that assessed running gait on different days separated testing by a minimum of 24 h [34, 140, 144], and repeated testing within 1 week [120, 149], 2 weeks [47, 87] or 1 month [117, 142], with one study repeating testing at 1 week and 6 months [138] (Table 2 of the ESM).

##### Speed Control

Thirty-one studies used controlled speeds, with the slowest speed set at 7 km/h [84] and the fastest speed set at 26 km/h [117]. Self-selected speeds were used in 21 studies, with a range from jogging [136] to maximum effort/sprint [86, 99, 100, 102, 104, 105, 129, 133]. An additional five studies combined controlled and self-selected speeds [62, 76, 116, 127, 135]. One study did not comment on the treadmill speeds used [93]. Twenty-seven studies included more than one speed in their protocol; consequently 32 studies examined the effect of running speed on the validity and/or reliability of outcomes obtained (Table 2 of the ESM).

##### Footwear Control

Most studies did not comment on the footwear used. Thirteen studies standardised the footwear of participants [45, 61, 62, 83, 85, 115, 116, 123, 124, 138, 142, 144, 149], 17 allowed participants to wear their own running shoes [27, 34, 79, 81, 87, 98, 99, 101, 103, 105, 106, 120, 128, 140, 141] and one study required participants to run unshod while wearing insoles under socks [150].

#### Validation Reference Measures

Twenty-four studies used a laboratory reference of 3D motion capture, 14 used a two-dimensional video analysis [26, 99–101, 105, 110, 116, 123, 128, 129, 133, 136, 142, 144], 17 used force plates [45, 63, 75, 76, 98, 104, 115, 121, 122, 124, 127, 130, 133, 136, 141, 142, 150], 17 used instrumented treadmills [38, 62, 81, 84, 93, 95–97, 103, 106, 123, 135, 140, 144, 149], one study compared measures to an accelerometery system implemented in the treadmill [34], 12 used timing gates/light barriers [61, 63, 86, 99, 100, 102, 104, 105, 110, 115, 127, 129], five compared to other wearable technology [45, 79, 97, 120, 130] and one study used a practitioner observed step count [117] (Table 2 of the ESM).

#### Validity and Reliability Findings

##### Foot/Shoe Mounted Devices

Most validity studies (*n* = 22) assessed shoe-mounted or foot-mounted devices. Reviewed studies showed that wearables could accurately measure stride time [85], speed, oscillation and GCT measures [79, 86, 134], step rate [93], FSP data [26, 81, 84, 116] and SL [100] using shoe or foot mounted wearable technology. Conflicting findings regarding the validity of joint kinematics using shoe-mounted accelerometers were demonstrated [73, 83, 94].

##### Tibia-Mounted Devices

Fifteen studies showed that tibial-mounted devices are valid for the detection of gait events [63, 127], step length [34], stride/step time [27, 34, 106], SF [34, 93, 106], tibial acceleration [34, 115] and vertical GRF [136]. However, stance and swing times collected using a gyroscope yielded poor-to-moderate agreement with optical motion capture [27]. One study did not consider the validity; however, it demonstrated that an accelerometer had good-to-moderate reliability for peak tibial acceleration at 1 week and 6 months [138].

##### Lower Back and Waist Mounted Devices

Fifteen articles reported that wearables on the pelvis, waist or lower back are accurate for identifying stride, step, stance duration [106], centre of mass vertical acceleration [75, 76], gait events [78], running speed, SL, SF [102, 106] and kinetic measures [104]. Outcomes such as GCT, flight time and peak vertical GRF have conflicting evidence regarding accuracy and reliability [24, 95, 96, 110, 120].

##### Upper Back Mounted Devices

Six studies reported that wearables located on the upper back [38, 105, 121, 129, 130, 133] had poor validity for examining gait symmetry [133], predicting GRF [121, 130], measuring velocity [129] and peak or average accelerations [38, 130], as well as poor reliability [130].

##### Multiple Device and Other Locations

Ten studies used more than one wearable in various locations and demonstrated good validity and reliability regarding spatiotemporal [106, 117] and kinematic and kinetic measures [61, 62, 94, 97, 103, 122, 138]. However, the validity varied between outcome measures (i.e. good accuracy for knee kinematics but poor for ankle kinematics) [93, 103, 105]. Furthermore, the measurement of SF and GCT using an accelerometer embedded in a wireless earbud showed good test–retest reliability, face validity and concurrent validity [135].

##### Pressure Insole Devices

Eleven studies reported on pressure insoles, with most studies attempting to correlate plantar pressures with GRF [45, 98, 122–124, 140–142, 144, 149, 150]. Findings suggest that insoles are generally valid and reliable for measuring temporal measures [98, 150] and kinetics, such as peak weight acceptance force, impulse and loading rate [124, 140, 142, 150]. However, other studies suggest that the validity of the device is dependent upon the force outcome measure [123, 149, 150]. Overall, the validity and reliability of pressure insoles appears to be system [128, 149], location [85, 101] and speed dependent [27, 99, 102, 127] (Table 2 of the ESM).

### Application Studies

The aims of the applied use of wearable technology for running gait analysis fell into broad categories of footwear, clothing (e.g. compression socks, insoles), surface (as mentioned in Sect. 3.7.1), intrinsic factors (e.g. sex, FSP), performance (e.g. experience, speed), fatigue, detecting gait parameters (e.g. relationships between gait parameters) and running injuries (Table 5).

**Table 5:** Summary of application of wearable technology

Application	*n*	References
Footwear and clothing	20	[37, 41, 43–45, 58, 61, 62, 64, 65, 70, 71, 109, 112–114, 119, 139, 146, 148]
Surface	16	[21, 35, 46, 53–55, 60, 66, 67, 87, 91, 118, 125, 131, 143, 151]
Intrinsic factors	15	[21, 23, 29, 31, 32, 36, 37, 40, 44, 52, 65, 68, 69, 80, 145]
Performance	17	[30–33, 35, 42, 47, 54, 62, 77, 82, 87, 90, 108, 118, 132, 137]
Fatigue	13	[19–21, 29, 31, 42, 59, 72, 88, 89, 111, 132, 143]
Detecting gait parameters	12	[23, 25, 28, 56, 57, 61, 62, 74, 90, 107, 116, 147]
Running injuries	6	[39, 48–52]

#### Footwear and Clothing

Eighteen studies investigated the effects of footwear on running gait parameters (Table 5). The majority of studies (*n* = 17) investigated different types of footwear on spatiotemporal, kinematics and kinetics, and generally the studies were consistent in evidencing that footwear construction has a substantial influence on some running gait outcome measures obtained by wearable technology, for example, significant differences in tibial acceleration [44, 64, 113, 114], SL [70] and loading parameters [37, 43, 45, 62, 65, 71, 148]. In contrast, other authors found no significant differences between shoe conditions [61, 112, 146]. In terms of clothing, Stickford et al. used wearable technology to examine whether wearing graduated lower-leg compression sleeves during exercise evokes changes in running biomechanics and Lucas-Cuevas et al. analysed the acute differences in stride parameters while running on a treadmill with custom-made and prefabricated insoles [119, 139].

#### Intrinsic Factors

Results of the 15 studies that investigated characteristics of sub-groups or intrinsic factors relating to performance suggested that running patterns were likely individual and task specific (Table 5) [29, 32, 80]. Of all the reviewed studies, five examined differences between male and female individuals [31, 32, 36–38], and three of these studies evidenced significant differences between sexes [32, 36, 37]. There were conflicting findings from the six studies that investigated the effects of FSP on running biomechanics [23, 40, 52, 65, 68, 69]. Key findings argue that no significant differences existed for total maximum force, force–time integral, peak pressure and pressure–time integral, but the total contact area of rear foot strikers was higher than that of non-rear foot strikers [68, 69]. In contrast, other studies demonstrated significant effects of the FSP on tibial acceleration, load rates and plantar pressure at varying plantar regions [23, 40, 52, 65]. Two studies examined morphological differences of the foot and the influence on running gait [44, 145]. Only one study examined the effects of age and anthropometric measures on running gait [31].

#### Performance

Of the applied studies that focused on performance aspects, 12 examined the influence of speed on running biomechanics [30, 31, 35, 42, 47, 54, 62, 77, 87, 118, 132, 137], four investigated the experience of participants [30, 32, 42, 118], one study examined the effects of altitude [82] and another study investigated gradient [90]. Associations of gait metrics with wellness and session perceived exertion was prospectively examined in one study [33] and specifically running kinematics in triathletes was investigated in another study [108].

#### Fatigue

Thirteen studies examined the effects of fatigue on running gait (Table 5). The findings are conflicting regarding if changes in running gait are fatigue induced and if this is dependent on experience level. Some suggest that GCT, flight time, trunk anterior–posterior acceleration, peak impact acceleration swing time, swing velocity and foot strike angles show significant changes with fatigue [42, 59, 89]. In contrast, others indicate no changes in spatiotemporal or FSP throughout the run [42, 88, 132]. Burns et al. suggested that SF changes only with speed and not fatigue [31]. Studies suggest that fatigue-induced changes do occur but may be subject specific [19–21, 111, 143] and dependent on experience/skill level [21, 29, 72] or fatigue state [89].

#### Detecting Gait Parameters

Twelve studies sought to investigate methods that detect or influence running gait outcome measures (Table 5). Studies sought to identify trends [25, 28], examine relationships between running gait parameters [23, 56, 74, 90, 116, 147] or investigate the effects of different methodologies on the outcome measures obtained [57, 61, 62, 107].

#### Running Injuries

Applied articles focusing on running related injuries (*n* = 6) sought to evaluate the effects of ankle taping, bracing and fibular reposition taping on running biomechanics [49], and to examine [52] and compare running gait parameters of injured and non-injured runners [39, 48, 50, 51]. Table 6 provides a summary of the most reported protocol features in the reviewed studies.

**Table 6:** Summary of commonly reported details in reviewed studies

Protocol feature	Most reported
Participants	Young adults [average age of 28.3 (± 7.0) years]
Sample size	Average of 26 (± 27)
Experience	Recreational runner
Environment	Indoor on a treadmill
Run duration/distance	Set distance or duration
Speed/pace	Controlled speed
Gradient	0–1%
Footwear	Standardised shoes
Type of wearable	Inertial measurement unit at 100 Hz
Outcome measures	Ground contact time, stride or step length, stride or step frequency and tibial acceleration
Sensor location	Fixed to the tibia or shoe
Number of wearables	One

### Usability

Only two studies sought to examine the usability, comfort or wearer’s perceptions of the device; both studies reported the wearables to be comfortable to wear and wearers did not feel affected in their movements [21, 125].

## Discussion

This review examined 131 studies that examined the use of wearable technology for running gait analysis. Explicitly, this review reported on: (1) methodologies employed to assess validity and reliability of wearables for running gait assessment; (2) application of wearables to assess running gait; and (3) commonly reported running gait outcomes and findings. This review has demonstrated that the use of wearable technology for running gait assessment is emerging, but further work is required to establish a standardised methodology and the validity or reliability of instrumentation. We have provided a comprehensive overview of wearable technology used for a running gait assessment, and here we provide recommendations for future work.

### Wearable Instrumentation

Wearable accelerometers, gyroscopes, IMUs (combined accelerometer, gyroscope and magnetometer) and pressure insoles were used within the reviewed studies to examine running gait. There was generally a lack of consistency across the reviewed studies for several factors that may impact the accuracy of wearable technology used for a running gait assessment, which included the data acquisition rate, data analysis methods, and location and number of wearables. Our findings show that IMUs are the most used wearables for running gait assessments (closely followed by pressure insoles), but most studies have focused on analysing acceleration data only rather than gyroscope and/or magnetometer data [11, 153]. However, evidence suggests that the use of all sensor data within a single IMU can improve the accuracy of movement quantification, particularly orientation [15, 27, 154–156]. Additionally, IMU accuracy for running gait assessments may have been impacted by the huge variation in sampling frequency and operating range between devices (4–1667 Hz, 2–70 g). For example, Mitschke et al. have shown that sampling frequency and operating range can influence the accuracy of outcome measures from IMUs, particularly when they are too low (e.g. < 100 Hz) to detect movement events [61]. Generally, wearables were deployed within the lower limb, with the tibia as the most common site (IMUs and pressure insoles) and most studies used one or two wearables, which may be because of the cost–benefit approach to the device set-up. For example, using multiple wearable technology inevitably costs more but there is a benefit of using multiple devices (that may be combined IMU and pressure insole systems), as more data acquisition allows for an increased accuracy of outcomes (e.g. gait events and spatiotemporal parameters) [157]. Most studies utilised only one wearable (IMU, accelerometer or gyroscope) to collect biomechanical data. However, it is important to consider the practicality and comfort of numerous wearables during natural running. Further research exploring the feasibility and necessity of utilising multiple wearables is required, or whether this can be condensed into one sensor, as this will enhance understanding of the optimal number and placement of wearables to deliver the most pertinent data while enabling a natural running gait.

A major issue in the approach to wearable instrument application is that only two studies examined the usability of the devices through engagement with end users. Wearable technology design and set-up can influence cost, usability and accuracy of the instruments, which may vary depending on the interests of different end users. Studies often lack considerations for the wearer’s physical, psychological and social preferences regarding the technology [158].

### Outcome Measures

This review has highlighted that there is a need for a comprehensive assessment and reporting of running gait outcomes, which may require combined ‘multi-modal’ (e.g. combination of IMU and pressure insoles, or accelerometer and pressure insole) wearables to examine running gait. The reviewed studies primarily limited their assessments to only the examination of selective spatiotemporal or kinematic outcomes; specifically SF, SL, tibial acceleration and GCT were the most common outcomes reported. Despite numerous studies establishing that running biomechanics cannot be described based on a single parameter [159–162], most studies focused on singular (or a select few) running gait outcomes, for example, GCT [99], SF [31, 117] or tibial acceleration [56, 118, 138]. Examination of selective parameters may explain in part the inconsistencies across study findings regarding the relationship between running biomechanics, performance and injury [161, 163–166]. Furthermore, comprehensive reporting and consistency in the literature is hindered by the lack of consistent terminology for running gait outcomes, for example, vertical oscillation of COM and stance duration have no relation to RRI [14, 163]. The lack of consensus is further impacted by the fact that there are no ‘gold-standard’ algorithms for the detection of running gait outcomes from wearable sensor set-ups, which likely explains the large volume of outcomes reported in the reviewed studies. In order to derive appropriate algorithms and report findings in a consistent manner, examination of multiple running gait outcomes (i.e. spatiotemporal, kinematic, kinetic) may require a combination of IMUs and pressure sensors, which allows for a comprehensive assessment and may improve outcome accuracy (e.g. vertical GRF is most accurate with the use of pressure sensors or multiple IMUs) [97], but the volume of outcomes may create other methodological issues when examining a finite number of individuals. Despite these limitations, it is pertinent to consider whether such idealist methodologies are clinically and practically feasible within a given context.

Outcomes obtained from small cohorts may not accurately represent the population being studied and may lead to poor statistical power or inconsistency across study findings. This was evidenced within the reviewed studies, as studies primarily investigated running gait in small sample sizes (i.e. *n* < 30) of young adults, which limits the generalisability of results. For example, Burns et al. demonstrated that the variability of an elite runner’s SF is linked to both speed and fatigue but not to any other characteristics of the runner [31]. In contrast, Reenalda et al. demonstrated that that changes in SF are dependent upon the individual; however, the authors were unable to perform an analysis at a group level because of their limited sample size (*n* = 3), thus stating that the observed effects of fatigue on running mechanics are confined to the runners analysed only and may not be representative for other runners [20]. The small sample sizes of the reviewed studies are surprising considering there is evidence from walking studies that gait analyses in a natural environment can be conducted on larger scales owing to the advancements in wearable technology [153, 167, 168]. The inclusion of larger sample sizes would facilitate the identification of subgroups of running patterns and the generalisability of the findings into the populations being studied. With the portability and ease of use of wearable technology, future studies should consider monitoring the running gait patterns of larger samples as it will allow for prospective studies and subgroups to be identified. Furthermore, only three studies examined running gait with an average age of over 50 years. However, none of the studies that examined older adults compared outcome measures to younger adults. Burns et al. noted that SF was not related to age; however, their sample only consisted of 20 participants, with an age range of 26–56 years (average age 38.1 ± 6.4 years) [31].

### Test Protocols

Differences among study protocols in running gait testing conditions, and the definition of outcome measures, limited the ability to directly compare outcomes across studies. Nonetheless these protocol differences highlight the versatility of wearables, proving they can provide data on realistic and spontaneous running scenarios. Treadmill running was the most common means to evaluate and quantify running gait. Use of a treadmill has the advantage of providing a standardised and reproducible environment where speed can be easily controlled and the required calibration volume for the optical system is considerably reduced. However, running speed is directly related to cardiovascular factors [169] and biomechanical factors [36, 170], and therefore imposing a set speed through a treadmill, rather than allowing runners to select the speed at which they are comfortable running, may produce alterations in running gait. Indeed, Zamparo et al. and Lussiana and Gindre indicated that self-selected speed related to individual energy-saving strategies [170, 171], and Kong et al. suggested that self-selected speeds may eliminate abnormal kinematic patterns [172]. Similarly, despite the known impact of the gradient on running gait, there were very few reviewed studies that examined this [173–175], but some studies did set the treadmill to 1% to compensate for the known differences between treadmill and over-ground running [176]. However, recent research has suggested that there may be more to consider than just the gradient when attempting to replicate over-ground running on the treadmill [177–179].

Protocols need to carefully consider where running is examined with wearables. Treadmill running may not truly reflect natural running behaviour, as Montgomery et al. demonstrated that non-motorised treadmills generate large reductions in peak tibial acceleration, large to very large increases in SF during running when compared to over-ground and motorised treadmills conditions [46]. Therefore, studies have moved beyond the laboratory to more natural running environments (i.e. indoor or outdoor running tracks, or sports venues), which has largely involved the examination of differences in running gait between different types of running surfaces [55, 67, 118, 180]. For example, when Hong et al. compared plantar loads when running on a treadmill, concrete and natural grass, it was shown that running on a treadmill induced lower peak plantar pressure and longer contact time for the total foot and two toe regions [55]. Additionally, several other reviewed studies suggest that running on natural grass may reduce stress on the musculoskeletal system and alter gait compared with running on a more rigid surface such as concrete or asphalt [66, 67, 151]. Similarly, there may be differences in kinematic and kinetic patterns when running on a treadmill compared with over-ground running [14, 53, 55, 67], which is not considered in running assessment protocols. Research has demonstrated that treadmill running may influence lower limb kinematic patterns, landing patterns and sagittal-plane foot strike angles when compared with over-ground running [166]. The differences exhibited can be attributed to several factors, such as treadmill running being unable to mimic instantaneous speed changes that inherently occur during over-ground running, as well as other environmental factors (i.e. irregular surfaces and gradient) [166, 181]. However, some consider treadmill running can be comparable to over-ground running depending on the outcome measures examined [166, 182], which highlights the need to carefully design protocols around specific running features of interest.

Most reviewed studies examined running over less than 1 min, but there was a lack of protocol consistency as studies varied in the number of steps, distance, number of trials and time of trials that they examined in runners, which made it difficult to generalise findings. Because of potential changes in running biomechanics over long runs, analysing an abundance of steps may be beneficial to gain consistency in outcomes [183]. Few authors have addressed a longitudinal running gait analysis, in terms of over an extended time period (e.g. training season) or over longer distances, using wearable technology [19–21, 28, 29, 31, 50, 82]. However, the studies that examined longer runs assessed running in a more natural environment (i.e. on a running track or outside over-ground) that allowed for greater time and distances to be studied compared with treadmill studies. Examining more and longer runs would potentially help divulge data regarding injury mechanisms and performance measures, thus informing practice by determining typical healthy running patterns as well as atypical gait patterns. Similarly, moving towards more realistic running environments that may be expected for commercial wearables was also reflected in the fact that a third of the reviewed studies allowed participants to wear their own running shoes (with a third requiring standardised shoes and the rest not reporting their footwear) [116, 119, 150]. This may signify a move towards attempting to use wearable technology with any individual running footwear, which would replicate commercial use.

### Validity and Reliability

Despite their widespread use, fewer than 10% of commercially available wearable technology are validated against an accepted ‘gold standard’ [184]. However, our review suggests that validation of research-grade (non-commercial) wearable technology for running gait assessment has been previously performed. Validity was performed by examining outcomes against ‘gold-standard’ reference measures (e.g. 3D motion capture, two-dimensional video capture, force plates, instrumented treadmills or timing gates). However, differences in laboratory references make it difficult to compare the validation of different wearable technology. For example, García-Pinillos et al. used a high-speed video analysis system (1000 Hz) as a laboratory reference [101], whereas the other studies have compared against the Optojump Next^®^ and video cameras [110], which is largely owing to the expense of laboratory references and the need for data capture in a more ‘natural’ setting (i.e. not in a gait laboratory). Photoelectric cell-based systems (i.e. Optojump Next^®^) and video measures were considered as adequate proxy systems given their demonstrated good validity in comparison to force platforms [185, 186], but they may not be the best reference system available. Findings from this review would suggest that outcomes from wearable technology for running gait should be validated against a known and accepted laboratory standard reference, such as 3D motion capture and force plates, to establish validity. Wearable technology was generally found to be valid for examining most running gait outcomes, particularly spatiotemporal measures, compared to laboratory references; however, this appears to be dependent upon the location of the wearable, the system and testing protocol (e.g. speeds) used, as well as the gait characteristics obtained [74, 85, 101, 130]. For example, accelerometers, gyroscopes or IMUs on the foot may provide the most accurate derivations of stride measures [99, 101, 128], but caution should be taken when using wearables located at the thoracic spine, as outcomes obtained from such placement appeared inadequate to predict gait symmetry, peak vertical and resultant GRF [38, 121, 129, 130, 133].

Reliability studies of wearables for running gait are less established, as the majority of studies included in this review used one experimental session, but there were several studies that performed test–retest runs within the same session [99, 103, 104, 110, 130, 134, 136] or two sessions on different days [47, 98, 117, 120, 138, 140, 144, 149]. Results demonstrated that outcomes of GCT, flight time and SF are reliable from a foot or lumbar spine placement [110], while foot-worn IMUs can provide reproducible calculations of stride time and SL [61]. Furthermore, placement on the tibia and lumbar and thoracic spine had excellent reliability for determining vertical GRF from accelerometer data [136].

### Application of Wearables

The reviewed studies of running gait measured with wearables focussed on several key areas of investigation, specifically injury, fatigue, performance, footwear/surface, methods for gait detection and intrinsic group factors. There were a range of differences in running gait outcomes with a group-based analysis of these factors. Despite differences being found, the specific spatiotemporal, kinematic and kinetic measures that could be used to best investigate certain aspects of running gait (e.g. fatigue, footwear) require further investigation. For example, while there were differences in running gait for those with current or previous injuries [48, 50, 52], there were no studies that examined outcomes for the risk of overuse running injuries.

Fatigue state was examined to understand changes in running mechanics with the potential for injury. However, few studies have exploited the benefits of wearable technology to explore real-world long-distance running sessions characterised by progressive fatigue [20, 21, 29, 82, 163]. Examining runners at varying stages or for the duration of a prolonged run in ecologically valid settings will add to the growing body of evidence using wearable technology to better understand the effects of training and fatigue on changes in running biomechanics [14, 19, 20]. These data can then be used to inform the runner of significant atypical changes in their running gait that may increase risk of RRIs. For example, it is well documented that running-related fatigue can affect running kinetics [187], kinematics [19, 188, 189] and certain spatiotemporal parameters [72, 82, 190]. Strohrmann et al. provides support for numerous cases, categorising changes into three groups: (1) changes that occurred for all runners (e.g. decrease of the heel lift); (2) changes that depended on the runner’s skill level (e.g. increase of foot contact duration); and (3) and changes that were highly dependent on the individual, (e.g. increase in shoulder rotation) [21].

Footwear was examined in a variety of studies, which primarily focussed on differences in running behaviour, with a suggestion that this may lead to injury. For example, Butler et al. evidenced that low-arch runners exhibited a reduction in peak tibial internal rotation in motion-controlled shoes compared with cushioned shoes, whereas high-arch runners experienced a lower peak positive acceleration in the cushioned shoe compared with the motion control shoe [44]. Similar to footwear, running surface has also been studied to examine the potential impact on performance and injury. For example, de Ruiter et al. demonstrated differences in running speed and GCT during outdoor over-ground running on flat terrain, and in varying weather conditions [79]. Studies have generally found that the footwear/surface can influence running gait characteristics, which needs to be carefully considered when making performance and injury risk/recovery decisions.

Intrinsic factors of runners may also impact running gait, with studies typically splitting cohorts into groups based on performance measures (amateur, elite), injury status (i.e. previously injured or not), age (young or old) or sex (male, female). The reviewed studies primarily assessed recreational runners, showing differences in running gait at different levels of performance [32]. For example, novice runners exhibit more pronounced changes in running kinematics in response to fatigue compared with elite runners [189]. Furthermore, Strohrmann et al. stratified runners based on their weekly mileage (experience), but did not find differences in mechanics across these groups [21]. However, not all studies have demonstrated differences between pre-determined intrinsic factor groups for certain outcome measures; for example, Burns et al. demonstrated that years of running experience did not significantly affect SF, and nor did sex [31]. There was a lack of sex-based analyses in the reviewed studies, which was surprising considering the established differences in running mechanics between male and female individuals [191, 192]. For example, Moltó et al. observed no significant differences in pelvic tilt or obliquity between the sexes; however, they did find significant differences in the range of pelvic rotation, with female runners presenting a greater range [36]. Queen et al. also evidenced different loading patterns between sexes and significant differences existed for the foot contact area (middle forefoot), with a maximum force at the lateral forefoot dependent on the shoe type [37]. Findings from Clermont et al. support this, highlighting the importance of separating runners into sex-specific subgroups first when classifying runners based on performance in order to better reflect the kinematic differences between sexes, and this is consistent with previous research [32, 193, 194]. This further highlights the need for a comprehensive assessment of running gait outcomes in order to detect characteristics that may be impacted by intrinsic factors, which would aid performance enhancement and reduce injury risk/occurrence [29, 72, 189].

### Practical Implications

This review provides insight into how wearable technology is used for investigating running biomechanics and there is an increasing body of evidence demonstrating its accuracy. Although beyond the scope of this review, with continued and improved use of wearables in runners, biomechanical data may be analysed using advanced techniques, such as machine learning and pattern recognition to enable identifying and tracking running demands without direct supervision. These predictive capabilities would be highly valuable to practitioners to monitor performance and fatigue measures in ecologically valid settings (Table 7).

**Table 7:** Summary of directions for future research using wearable technology

Future research directions	
Test the validity and reliability prior to performing clinical or applied studies Multimodal wearable technology may give more comprehensive assessment of running gait Studies require an appropriate sample size Using wearable technology during natural outdoor running over time would help confirm laboratory findings or expand upon our knowledge Examine effects of age and sex on running gait outcome measures Report outcome measures as comprehensively as possible Investigate the usability, comfort, as well as the wearer’s physical, psychological and social preferences regarding technology	

### Review Limitations

Several limitations of the review must be considered. The search was limited to four databases, albeit integrated by reference lists and hand searches to identify other relevant papers. The use of stringent exclusion criteria may lead to the omission of potentially relevant data. First, articles not published in English pose a language bias regarding article selection. Additionally, sensor modality was restricted to wearable accelerometers, gyroscopes, magnetometers or a combination of those (IMU), or pressure insoles, thus excluding GPS or mobile phone applications, which are common amongst runners [195]. Because of the varying definitions and methods of calculation, studies were also excluded if they focused solely on shock, stiffness or neuromuscular load. We excluded studies that applied interventions as this would influence the gait outcomes and may not be representative of a runner’s typical gait. Finally, because of the size and heterogeneity of the articles included within this review, no meta-analysis or formal quality assessment of each study was performed.

## Conclusions

Wearable technology is rapidly becoming a feasible means to quantify running biomechanics in a more ecologically valid manner, with applications in sports medicine and sports performance. This review highlighted that most studies that have examined running gait using wearable sensors have done so with young adult recreational runners, using one IMU sensor (on shoe or tibia), with participants running on a treadmill and reporting outcomes of GCT, SL, SF and tibial acceleration. While this review comprehensively synthesised a large (*n* = 131) number of previous studies, future studies are needed to determine optimal outcome definitions, sensor site, type of sensor and outcomes of interest for running gait.

### Electronic supplementary material

Below is the link to the electronic supplementary material.

Supplementary file1 (DOCX 121 KB)

Supplementary file2 (DOCX 306 KB)

## Appendix 1 Data extraction form

**Table:**

**Study**
**General information** Title, authors(s), year
**Study characteristics** Study type (i.e. validation, reliability, application)
**Participant characteristics ** Characteristics; age, sex, height, weight, numbers in each group, type (e.g. injured, RFS, recreationally active)
**Wearable Technology** Type of device (i.e. IMU, accelerometer, pressure insole) Model and brand of wearable technology (e.g. Shimmer3, Shimmer Inc.) Number of devices used Location of device(s) Device characteristics; accelerometer range, gyroscope range, sampling frequency, dimensions, weight
**Methodology** Test protocol Environment (e.g. indoor, outdoor), surface type (e.g. road, treadmill), gradient speed(s) Analysed distance/time/steps
**Outcome measures** What was measured using wearable technology? Reference measures or additional tools used (e.g. 3D motion capture, EMG)
**Analysis** Statistical techniques used
**Results** Main findings according to the study author(s)

## Declarations

### Availability of data and material

All data generated or analysed during this study are included in this published article (and its supplementary information files).

## References

[1] Andersen JJ. The state of running 2019. 2021. Available from: https://runrepeat.com/state-of-running. Accessed 27 Jul 2021.

[2] Fokkema T, Hartgens F, Kluitenberg B (2019). Reasons and predictors of discontinuation of running after a running program for novice runners. J Sci Med Sport.

[3] Dingenen B, Barton C, Janssen T, Benoit A, Malliaras P (2018). Test-retest reliability of two-dimensional video analysis during running. Phys Ther Sport.

[4] Ferber R, Macdonald S. Running mechanics and gait analysis. Champaign, IL: Human Kinetics; 2014.

[5] Pipkin A, Kotecki K, Hetzel S, Heiderscheit B (2016). Reliability of a qualitative video analysis for running. J Orthop Sports Phys Ther.

[6] Wang L, Hu W, Tan T (2003). Recent developments in human motion analysis. Pattern Recogn.

[7] Chen KY, Janz KF, Zhu W, Brychta RJ (2012). Redefining the roles of sensors in objective physical activity monitoring. Med Sci Sports Exerc.

[8] Tao W, Liu T, Zheng R, Feng H (2012). Gait analysis using wearable sensors. Sensors (Basel).

[9] Dugan SA, Bhat KP (2005). Biomechanics and analysis of running gait. Phys Med Rehabil Clin N Am.

[10] Higginson BK (2009). Methods of running gait analysis. Curr Sports Med Rep.

[11] Norris M, Anderson R, Kenny IC (2014). Method analysis of accelerometers and gyroscopes in running gait: a systematic review. J Sports Eng Technol.

[12] Mann R, Malisoux L, Urhausen A, Meijer K, Theisen D (2016). Plantar pressure measurements and running-related injury: a systematic review of methods and possible associations. Gait Posture.

[13] Chambon N, Delattre N, Guéguen N, Berton E, Rao G (2015). Shoe drop has opposite influence on running pattern when running overground or on a treadmill. Eur J Appl Physiol.

[14] Willy RW (2018). Innovations and pitfalls in the use of wearable devices in the prevention and rehabilitation of running related injuries. Phys Ther Sport.

[15] Stuart S, Powell D, Marshall SJ, Godfrey A, Stuart S (2021). Chapter 15. Sports medicine: bespoke player management. Digital health.

[16] Chan-Roper M, Hunter I, Myrer JW, Seeley MK (2012). Kinematic changes during a marathon for fast and slow runners. J Sports Sci Med.

[17] Larson P, Higgins E, Kaminski J (2011). Foot strike patterns of recreational and sub-elite runners in a long-distance road race. J Sports Sci.

[18] Degache F, Morin J-Bt, Oehen L (2016). Running mechanics during the world's most challenging mountain ultramarathon. Int J Sports Physiol Perform.

[19] Reenalda J, Maartens E, Buurke JH, Gruber AH (2019). Kinematics and shock attenuation during a prolonged run on the athletic track as measured with inertial magnetic measurement units. Gait Posture.

[20] Reenalda J, Maartens E, Homan L, Buurke JHJ (2016). Continuous three dimensional analysis of running mechanics during a marathon by means of inertial magnetic measurement units to objectify changes in running mechanics. J Biomech.

[21] Strohrmann C, Harms H, Kappeler-Setz C, Troster G (2012). Monitoring kinematic changes with fatigue in running using body-worn sensors. IEEE Transact Inform Technol Biomed.

[22] Page MJ, McKenzie JE, Bossuyt PM (2021). The PRISMA 2020 statement: an updated guideline for reporting systematic reviews. PLOS Med.

[23] Napier C, Fridman L, Blazey P, Tran N, Michie TV, Schneeberg A (2022). Differences in peak impact accelerations among foot strike patterns in recreational runners. Front Sports Act Living.

[24] Day EM, Alcantara RS, McGeehan MA, Grabowski AM, Hahn ME (2021). Low-pass filter cutoff frequency affects sacral-mounted inertial measurement unit estimations of peak vertical ground reaction force and contact time during treadmill running. J Bomech.

[25] Akhlaghi F, Pepper M, Daw J, Potter MJ (1994). In-shoe step-to-step pressure variations. Foot.

[26] van Werkhoven H, Farina KA, Langley MH. Using a soft conformable foot sensor to measure changes in foot strike angle during running. Sports. 2019;7(8). 10.3390/sports7080184 PMC6723362 31362349

[27] McGrath D, Greene B, O'Donovan K, Caulfield B (2012). Gyroscope-based assessment of temporal gait parameters during treadmill walking and running. Sports Eng.

[28] Ahamed NU, Benson LC, Clermont CA, Pohl AJ, Ferber R (2019). New considerations for collecting biomechanical data using wearable sensors: how does inclination influence the number of runs needed to determine a stable running gait pattern?. Sensors.

[29] Clermont CA, Benson LC, Edwards WB, Hettinga BA, Ferber R (2019). New considerations for wearable technology data: changes in running biomechanics during a marathon. J Appl Biomech.

[30] Shun-Ping L, Wen-Hsu S, Fon-Chu K, Kuo TBJ, Jin-Jong C (2014). Impact of center-of-mass acceleration on the performance of ultramarathon runners. J Human Kinet.

[31] Burns GT, Zendler JM, Zernicke RF (2019). Step frequency patterns of elite ultramarathon runners during a 100-km road race. J Appl Physiol.

[32] Clermont CA, Benson LC, Kobsar D, Osis ST, Ferber R (2019). Running patterns for male and female competitive and recreational runners based on accelerometer data. J Sports Sci.

[33] DeJong Lempke AF, Hart JM, Hryvniak DJ, Rodu JS, Hertel J (2022). Prospective running assessments among division I cross-country athletes. Phys Ther Sport.

[34] Encarnacion-Martinez A, Perez-Soriano P, Sanchis-Sanchis R, Garcia-Gallart A, Berenguer-Vidal R (2021). Validity and reliability of an instrumented treadmill with an accelerometry system for assessment of spatio-temporal parameters and impact transmission. Sensors.

[35] Encarnacion-Martinez A, Catalá-Vilaplana I, Berenguer-Vidal R, Sanchis-Sanchis R, Ochoa-Puig B, Pérez-Soriano P (2021). Treadmill and running speed effects on acceleration impacts: curved non-motorized treadmill vs. conventional motorized treadmill. Int J Environ Res Public Health.

[36] Moltó IN, Albiach JP, Amer-Cuenca JJ, Segura-Ortí E, Gabriel W, Martínez-Gramage J (2020). Wearable sensors detect differences between the sexes in lower limb electromyographic activity and pelvis 3D kinematics during running. Sensors.

[37] Queen RM, Abbey AN, Wiegerinck JI, Yoder JC, Nunley JA (2010). Effect of shoe type on plantar pressure: a gender comparison. Gait Posture.

[38] Wundersitz DWT, Gastin PB, Richter C, Robertson SJ, Netto KJ (2015). Validity of a trunk-mounted accelerometer to assess peak accelerations during walking, jogging and running. Eur J Sport Sci.

[39] Fereydounnia S, Shadmehr A, Salemi P, Amiri S (2022). Comparison of ROM, perceived tightness, and kinetic variables during balance, walking, and running tasks in athletes with and without hamstring tightness using sensor insoles. Sport Sci Health.

[40] Kernozek TW, Meardon S, Vannatta CN (2014). In-shoe loading in rearfoot and non-rearfoot strikers during running using minimalist footwear. Int J Sports Med.

[41] Sinclair J, Naemi R, Chockalingam N, Taylor PJ, Shore H (2015). The effects of shoe temperature on the kinetics and kinematics of running. Footwear Sci.

[42] Brahms CM, Zhao Y, Gerhard D, Barden JM (2020). Long-range correlations and stride pattern variability in recreational and elite distance runners during a prolonged run. Gait Posture.

[43] Bräuer S, Kiesewetter P, Milani TL, Mitschke C (2021). The ‘ride’ feeling during running under field conditions—objectified with a single inertial measurement unit. Sensors.

[44] Butler RJ, Hamill J, Davis I (2007). Effect of footwear on high and low arched runners' mechanics during a prolonged run. Gait Posture.

[45] Hennig EM, Milani TL (1995). In-shoe pressure distribution for running in various types of footwear. J Appl Biomech.

[46] Montgomery G, Abt G, Dobson C, Smith T, Ditroilo M (2016). Tibial impacts and muscle activation during walking, jogging and running when performed overground, and on motorised and non-motorised treadmills. Gait Posture.

[47] Kernozek TW, Zimmer KA (2000). Reliability and running speed effects of in-shoe loading measurements during slow treadmill running. Foot Ankle Int.

[48] Meardon SA, Hamill J, Derrick TR (2011). Running injury and stride time variability over a prolonged run. Gait Posture.

[49] Gregory C, Koldenhoven RM, Higgins M, Hertel J (2019). External ankle supports alter running biomechanics: a field-based study using wearable sensors. Physiol Meas.

[50] Koldenhoven RM, Virostek A, DeJong AF, Higgins M, Hertel J (2020). Increased contact time and strength deficits in runners with exercise-related lower leg pain. J Athl Train.

[51] Pla GA, Hollville E, Schutte K, Vanwanseele B (2021). The use of a single trunk-mounted accelerometer to detect changes in center of mass motion linked to lower-leg overuse injuries: a prospective study. Sensors.

[52] Tenforde AS, Hayano T, Jamison ST, Outerleys J, Davis IS (2020). Tibial acceleration measured from wearable sensors is associated with loading rates in injured runners. PM R.

[53] Fu W, Fang Y, Liu DMS, Wang L, Ren S, Liu Y (2015). Surface effects on in-shoe plantar pressure and tibial impact during running. J Sport Health Sci.

[54] Hollis CR, Koldenhoven RM, Resch JE, Hertel J (2021). Running biomechanics as measured by wearbale sensors: effects of speed and surface. Sports Biomech.

[55] Hong Y, Wang L, Li JX, Zhou JH (2012). Comparison of plantar loads during treadmill and overground running. J Sci Med Sport.

[56] Johnson CD, Outerleys J, Davis IS (2021). Relationships between tibial acceleration and ground reaction force measures in the medial-lateral and anterior-posterior planes. J Biomech.

[57] Johnson CD, Outerleys J, Tenforde AS, Davis IS (2020). A comparison of attachment methods of skin mounted inertial measurement units on tibial accelerations. J Biomech.

[58] McNair PJ, Marshall RN (1994). Kinematic and kinetic-parameters associated with running in different shoes. Br J Sports Med.

[59] Meyer F, Falbriard M, Mariani B, Aminian K, Millet GP (2021). Continuous analysis of marathon running using inertial sensors: hitting two walls?. Int J Sports Med.

[60] Milner CE, Hawkins JL, Aubol KG (2020). Tibial acceleration during running is higher in field testing than indoor testing. Med Sci Sports Exerc.

[61] Mitschke C, Kiesewetter P, Milani TL. The effect of the accelerometer operating range on biomechanical parameters: stride length, velocity, and peak tibial acceleration during running. Sensors. 2018;18(1). 10.3390/s18010130 PMC5795747 29303986

[62] Napier C, Willy W, Hannigan BC, McCann R, Menon C (2021). DThe effect of footwear, running speed, and location on the validity of two commercially available inertial measurement units during running. Front Sports Act Living.

[63] Sinclair J, Hobbs SJ, Protheroe L, Edmundson CJ, Greenhalgh A (2013). Determination of gait events using an externally mounted shank accelerometer. J Appl Biomech.

[64] Sinclair J (2017). The influence of minimalist, maximalist and conventional footwear on impact shock attenuation during running. Move Sports Sci.

[65] Sun X, Yang Y, Wang L, Zhang X, Fu W (2018). Do strike patterns or shoe conditions have a predominant influence on foot loading?. J Hum Kinet.

[66] Tessutti V, Ribeiro AP, Trombini-Souza F, Sacco IC (2012). Attenuation of foot pressure during running on four different surfaces: asphalt, concrete, rubber, and natural grass. J Sports Sci.

[67] Wang L, Hong Y, Li JX, Zhou JH (2012). Comparison of plantar loads during running on different overground surfaces. Res Sports Med.

[68] Wei Z, Zhang Z, Jiang J, Zhang Y, Wang L (2019). Comparison of plantar loads among runners with different strike patterns. J Sports Sci.

[69] Wei Z, Li JX, Fu W, Wang L (2020). Plantar load characteristics among runners with different strike patterns during preferred speed. J Exerc Sci Fit.

[70] Wunsch T, Kroll J, Stoggl T, Schwameder H (2017). Effects of a structured midsole on spatio-temporal variables and running economy in overground running. Eur J Sport Sci.

[71] Roca-Dols A, Losa-Iglesias ME, Sánchez-Gómez R (2018). Effect of the cushioning running shoes in ground contact time of phases of gait. J Mech Behav Biomed Mater.

[72] Clermont CA, Pohl AJ, Ferber R (2020). Fatigue-related changes in running gait patterns persist in the days following a marathon race. J Sport Rehabil.

[73] Gholami M, Napier C, Menon C (2020). Estimating lower extremity running gait kinematics with a single accelerometer: a deep learning approach. Sensors.

[74] Glassbrook DJ, Fuller JT, Alderson JA, Doyle TLA (2020). Foot accelerations are larger than tibia accelerations during sprinting when measured with inertial measurement units. J Sports Sci.

[75] Lee JB, Mellifont RB, Burkett BJ (2010). The use of a single inertial sensor to identify stride, step, and stance durations of running gait. J Sci Med Sport.

[76] Lee JB, Sutter KJ, Askew CD, Burkett BJ (2010). Identifying symmetry in running gait using a single inertial sensor. J Sci Med Sport.

[77] Provot T, ChiementinF X, Bolaers F, Murer S (2019). Effect of running speed on temporal and frequency indicators from wearable MEMS accelerometers. Sports Biomech..

[78] Winter SC, Lee JB, Leadbetter RI, Gordon SJ (2016). Validation of a single inertial sensor for measuring running kinematics overground during a prolonged run. J Fit Res.

[79] de Ruiter CJ, Van Oeveren B, Francke A, Zijlstra P, Van Dieen JH (2016). Running speed can be predicted from foot contact time during outdoor over ground running. PLoS One.

[80] DeJong AF, Hertel J (2020). Outdoor running activities captured using wearable sensors in adult competitive runners. Int J Athlet Ther Train.

[81] DeJong AF, Hertel J (2020). Validation of foot-strike assessment using wearable sensors during running. J Athlet Train.

[82] Jeker D, Falbriard M, Vernillo G (2020). Changes in spatio-temporal gait parameters and vertical speed during an extreme mountain ultra-marathon. Eur J Sport Sci.

[83] Koska D, Gaudel J, Hein T, Maiwald C (2018). Validation of an inertial measurement unit for the quantification of rearfoot kinematics during running. Gait Posture.

[84] Shiang TY, Hsieh TY, Lee YS, et al. Determine the foot strike pattern using inertial sensors. J Sensors. 2016.

[85] Zrenner M, Kuderle A, Roth N, Jensen U, Dumler B, Eskofier BM (2020). Does the position of foot-mounted IMU sensors influence the accuracy of spatio-temporal parameters in endurance running?. Sensors.

[86] Blauberger P, Horsch A, Lames M (2021). Detection of ground contact times with inertial sensors in elite 100-m sprints under competitive field conditions. Sensors.

[87] Kozinc Ž, Smajla D, Šarabon N. The reliability of wearable commercial sensors for outdoor assessment of running biomechanics: the effect of surface and running speed. Sports Biomech. 2022;1–14. 10.1080/14763141.2021.2022746 35019817

[88] Farina KA, Needle AR, van Werkhoven H (2021). Continuous tracking of foot strike pattern during a maximal 800-meter run. Sensors.

[89] Prigent G, Apte S, Paraschiv-Ionescu A, Besson C, Gremeaux V, Aminian K (2022). Concurrent evolution of biomechanical and physiological parameters with running-induced acute fatigue. Front Physiol.

[90] Perrotin N, Gardan N, Lesprillier A (2018). Biomechanics of trail running performance: quantification of spatio-temporal parameters by using low cost sensors in ecological conditions. Appl Sci.

[91] Bailey J, Mata T, Mercer JA (2017). Is the relationship between stride length, frequency, and velocity influenced by running on a treadmill or overground?. Int J Exerc Sci.

[92] Chew D-K, Ngoh KJ-H, Gouwanda D, Gopalai AA (2018). Estimating running spatial and temporal parameters using an inertial sensor. Sports Eng.

[93] de Fontenay BP, Roy JS, Dubois B, Bouyer L, Esculier JF (2020). Validating commercial wearable sensors for running gait parameters estimation. IEEE Sens J.

[94] Kim BH, Hong SH, Oh IW, Lee YW, Kee IH, Lee SY (2021). Measurement of ankle joint movements using IMUs during running. Sensors (Basel).

[95] Patoz A, Lussiana T, Breine B, Gindre C, Malatesta D (2021). Estimating effective contact and flight times using a sacral-mounted inertial measurement unit. J Biomech.

[96] Patoz A, Lussiana T, Breine B, Gindre C, Malatesta D (2022). A single sacral-mounted inertial measurement unit to estimate peak vertical ground reaction force, contact time, and flight time in running. Sensors.

[97] Wouda FJ, Giuberti M, Bellusci G (2018). Estimation of vertical ground reaction forces and sagittal knee kinematics during running using three inertial sensors. Front Physiol.

[98] Zhang H, Guo Y, Zanotto D (2020). Accurate ambulatory gait analysis in walking and running using machine learning models. IEEE Trans Neural Syst Rehabil Eng.

[99] Ammann R, Taube W, Wyss T (2016). Accuracy of Partwear Inertial Sensor and Optojump optical measurement system for measuring ground contact time during running. J Strength Cond Res.

[100] de Ruiter CJ, van Dieen JH (2019). Stride and step length obtained with inertial measurement units during maximal sprint acceleration. Sports.

[101] García-Pinillos F, Chicano-Gutiérrez JM, Ruiz-Malagón EJ, Roche-Seruendo LE (2020). Influence of RunScribe™ placement on the accuracy of spatiotemporal gait characteristics during running. Proc Inst Mech Eng Part P J Sports Eng Technol.

[102] Machulik C, Hamacher D, Lindlein K, Zech A, Hollander K (2020). Validation of an inertial measurement unit based magnetic timing gate system during running and sprinting. German J Sports Med.

[103] Nuesch C, Roos E, Pagenstert G, Mundermann A (2017). Measuring joint kinematics of treadmill walking and running: comparison between an inertial sensor based system and a camera-based system. J Biomech.

[104] Setuain I, Lecumberri P, Ahtiainen JP, Mero AA, Häkkinen K, Izquierdo M (2018). Sprint mechanics evaluation using inertial sensor-based technology: a laboratory validation study. Scand J Med Sci Sports.

[105] de Ruiter CJ, Wilmes E, Brouwers SAJ, Jagers EC, van Dieën JH. Concurrent validity of an easy-to-use inertial measurement unit-system to evaluate sagittal plane segment kinematics during overground sprinting at different speeds. Sports Biomech. 2022. 10.1080/14763141.2022.2056076 35353032

[106] Struber L, Ledouit S, Daniel O, Barraud PA, Nougier V (2021). Reliability of human running analysis with low-cost inertial and magnetic sensor arrays. IEEE Sens J.

[107] Florenciano Restoy JL, Solé-Casals J, Borràs-Boix X (2021). IMU-based effects assessment of the use of foot orthoses in the stance phase during running and asymmetry between extremities. Sensors (Basel).

[108] Fraeulin L, Maurer-Grubinger C, Holzgreve F, Groneberg DA, Ohlendorf D (2021). Comparison of joint kinematics in transition running and isolated running in elite triathletes in overground conditions. Sensors.

[109] Huang M, Mo S, Pak-Kwan Chan P, Chan ZYS, Zhang-Lea JH, Cheung RTH. The influence of running shoes on familiarization time for treadmill running biomechanics evaluation. Sports Biomech. 2022. 10.1080/14763141.2022.2046144 35232315

[110] Gindre C, Lussiana T, Hebert-Losier K, Morin J-B (2016). Reliability and validity of the Myotest^®^ for measuring running stride kinematics. J Sports Sci.

[111] Mizrahi J, Verbitsky O, Isakov E, Daily D (2000). Effect of fatigue on leg kinematics and impact acceleration in long distance running. Hum Move Sci.

[112] Sinclair J, Dillon S (2016). The influence of energy boost and springblade footwear on the kinetics and kinematics of running. Hum Move.

[113] Sinclair J, Franks C, Goodwin JF, Naemi R, Chockalingam N (2014). Influence of footwear designed to boost energy return on the kinetics and kinematics of running compared to conventional running shoes. Comp Exerc Physiol.

[114] Sinclair J, Rooney E, Naemi R, Atkins S, Chockalingam N. Effects of footwear variations on three-dimensional kinematics and tibial accelerations of specific movements in American football. J Mech Med Biol. 2017;17(2).

[115] Sinclair J, Sant B (2017). The effects of cross-fit footwear on the kinetics and kinematics of running. Footwear Sci.

[116] Giandolini M, Poupard T, Gimenez P (2014). A simple field method to identify foot strike pattern during running. J Biomech.

[117] Rowlands AV, Stone MR, Eston RG (2007). Influence of speed and step frequency during walking and running on motion sensor output. Med Sci Sports Exerc.

[118] Boey H, Aeles J, Schutte K, Vanwanseele B (2017). The effect of three surface conditions, speed and running experience on vertical acceleration of the tibia during running. Sports Biomech.

[119] Lucas-Cuevas AG, Camacho-García A, Llinares R, Priego Quesada JI, Llana-Belloch S, Pérez-Soriano P (2017). Influence of custom-made and prefabricated insoles before and after an intense run. PLoS ONE.

[120] Gouttebarge V, Wolfard R, Griek N, de Ruiter CJ, Boschman JS, van Dieen JH (2015). Reproducibility and validity of the Myotest for measuring step frequency and ground contact time in recreational runners. J Hum Kinet.

[121] Nedergaard NJ, Verheul J, Drust B (2018). The feasibility of predicting ground reaction forces during running from a trunk accelerometry driven mass-spring-damper model. Peer J.

[122] Sakamoto K, Tsujioka C, Sasaki M, Miyashita T, Kitano M, Kudo S (2021). Validity and reproducibility of foot motion analysis using a stretch strain sensor. Gait Posture.

[123] Seeley MK, Evans-Pickett A, Collins GQ (2020). Predicting vertical ground reaction force during running using novel piezoresponsive sensors and accelerometry. J Sports Sci.

[124] Stöggl T, Martiner A (2017). Validation of Moticon's OpenGo sensor insoles during gait, jumps, balance and cross-country skiing specific imitation movements. J Sports Sci.

[125] Tao H, Joyce L, Kozak B, Luiken J, Wendt N (2019). Spatiotemporal comparison of overground and treadmill running with pressure sensor insoles in Division I Collegiate runners. Int J Sports Phys Ther.

[126] Hennig EM, Nilani TL (1995). In-shoe pressure distribution for running in various types of footwear. J Appl Biomech.

[127] Fadillioglu C, Stetter BJ, Ringhof S, Krafft FC, Sell S, Stein T (2020). Automated gait event detection for a variety of locomotion tasks using a novel gyroscope-based algorithm. Gait Posture.

[128] García-Pinillos F, Latorre-Román P, Soto-Hermoso VM (2019). Agreement between the spatiotemporal gait parameters from two different wearable devices and high-speed video analysis. PLoS ONE.

[129] Alexander JP, Hopkinson TL, Wundersitz DW, Serpell BG, Mara JK, Ball NB (2016). Validity of a wearable accelerometer device to measure average acceleration values during high-speed running. J Strength Cond Res.

[130] Edwards S, White S, Humphreys S, Robergs R, O'Dwyer N (2019). Caution using data from triaxial accelerometers housed in player tracking units during running. J Sports Sci.

[131] Garcia MC, Gust G, Bazett-Jones DM (2021). Tibial acceleration and shock attenuation while running over different surfaces in a trail environment. J Sci Med Sport.

[132] Howe CCF, Swann N, Spendiff O, Kosciuk A, Pummell EKL, Moir HJ (2021). Performance determinants, running energetics and spatiotemporal gait parameters during a treadmill ultramarathon. Eur J Appl Physiol.

[133] Kenneally-Dabrowski CJB, Serpell BG, Spratford W (2018). Are accelerometers a valid tool for measuring overground sprinting symmetry?. Int J Sports Sci Coach.

[134] Navalta JW, Montes J, Bodell NG (2019). Reliability of trail walking and running tasks using the Stryd power meter. Int J Sports Med.

[135] Nijs A, Beek PJ, Roerdink M (2021). Reliability and validity of running cadence and stance time derived from instrumented wireless earbuds. Sensors.

[136] Raper DP, Witchalls J, Philips EJ, Knight E, Drewlls MK, Waddington G (2018). Use of a tibial accelerometer to measure ground reaction force in running: a reliability and validity comparison with force plates. J Sci Med Sport.

[137] Sheerin KR, Besier TF, Reid D (2020). The influence of running velocity on resultant tibial acceleration in runners. Sports Biomech.

[138] Sheerin KR (2018). The one-week and six-month reliability and variability of three-dimensional tibial acceleration in runners. Sports Biomech.

[139] Stickford AS, Chapman RF, Johnston JD, Stager JM (2015). Lower-leg compression, running mechanics, and economy in trained distance runners. Int J Sports Physiol Perform.

[140] Burns GT, Deneweth Zendler J, Zernicke RF (2019). Validation of a wireless shoe insole for ground reaction force measurement. J Sports Sci.

[141] Chumanov ES, Remy CD, Thelen DG (2010). Computational techniques for using insole pressure sensors to analyse three-dimensional joint kinetics. Comput Methods Biomech Biomed Eng.

[142] Cramer LA, Wimmer MA, Malloy P, O'Keefe  JA, Knowlton CB, Ferrigno C (2022). Validity and reliability of the Insole3 instrumented shoe insole for ground reaction force measurement during walking and running. Sensors.

[143] García-Pérez JA, Pérez-Soriano P, Llana S, Martínez-Nova A, Sánchez-Zuriaga D (2013). Effect of overground vs treadmill running on plantar pressure: influence of fatigue. Gait Posture.

[144] Mann R, Malisoux L, Brunner R (2014). Reliability and validity of pressure and temporal parameters recorded using a pressure-sensitive insole during running. Gait Posture.

[145] Mei Q, Fernandez J, Fu W, Feng N, Gu Y (2015). A comparative biomechanical analysis of habitually unshod and shod runners based on a foot morphological difference. Hum Move Sci.

[146] Mei Q, Graham M, Gu Y (2014). Biomechanical analysis of the plantar and upper pressure with different sports shoes. Int J Biomed Eng Technol.

[147] Musgjerd T, Anason J, Rutherford D, Kernozek TW (2021). Effect of increasing running cadence on peak impact force in an outdoor environment. Int J Sports Phys Ther.

[148] Orendurff MS, Rohr ES, Segal AD, Medley JW, Green JR, Kadel NJ (2008). Regional foot pressure during running, cutting, jumping, and landing. Am J Sports Med.

[149] Renner KE, Williams DSB, Queen RM (2019). The reliability and validity of the Loadsol(^®^) under various walking and running conditions. Sensors (Basel).

[150] Seiberl W, Jensen E, Merker J, Leitel M, Schwirtz A (2018). Accuracy and precision of Loadsol^®^ insole force-sensors for the quantification of ground reaction force-based biomechanical running parameters. Eur J Sport Sci.

[151] Tessutti V, Trombini-Souza F, Ribeiro AP, Nunes AL, Sacco Ide C (2008). In-shoe plantar pressure distribution during running on natural grass and asphalt in recreational runners. J Sci Med Sport.

[152] Kozinc Ž, Smajla D, Šarabon N. The reliability of wearable commercial sensors for outdoor assessment of running biomechanics: the effect of surface and running speed. Sports Biomech. 2022;1–14. 10.1080/14763141.2021.2022746 35019817

[153] Benson LC, Clermont CA, Bošnjak E, Ferber R (2018). The use of wearable devices for walking and running gait analysis outside of the lab: a systematic review. Gait Posture.

[154] Sabatini AM (2011). Estimating three-dimensional orientation of human body parts by inertial/magnetic sensing. Sensors.

[155] El-Gohary M, McNames J (2012). Shoulder and elbow joint angle tracking with inertial sensors. IEEE Trans Biomed Eng.

[156] El-Gohary M, McNames J (2015). Human joint angle estimation with inertial sensors and validation with a robot arm. IEEE Trans Biomed Eng.

[157] Storm FA, Buckley CJ, Mazzà C (2016). Gait event detection in laboratory and real life settings: accuracy of ankle and waist sensor based methods. Gait Posture.

[158] Godfrey A, Hetherington V, Shum H, Bonato P, Lovell NH, Stuart S (2018). From A to Z: wearable technology explained. Maturitas.

[159] Black MI, Black MI, Handsaker JC, Allen SJ, Forrester SE, Folland JP (2018). Is there an optimal speed for economical running?. Int J Sports Physiol Perform.

[160] Folland JP, Allen SJ, Black MI, Handsaker JC, Forrester SE (2017). Running technique is an important component of running economy and performance. Med Sci Sports Exerc.

[161] van Oeveren BT, e Ruiter CJ, Beek PJ, van Dieën JH. The biomechanics of running and running styles: a synthesis. Sports Biomech. 2021;1–39. 10.1080/14763141.2021.1873411 33663325

[162] Williams KR, Cavanagh PR (1987). Relationship between distance running mechanics, running economy, and performance. J Appl Physiol.

[163] Brindle RA, Taylor JB, Rajek C, Weisbrod A, Ford KR (2020). Association between temporal spatial parameters and overuse injury history in runners: a systematic review and meta-analysis. Sports Med.

[164] Ceyssens L, Vanelderen R, Barton C, Malliaras P, Dingenen B (2019). Biomechanical risk factors associated with running-related injuries: a systematic review. Sports Med.

[165] Mousavi SH, Hijmans JM, Rajabi R, Diercks R, Zwerver J, van der Worp H (2019). Kinematic risk factors for lower limb tendinopathy in distance runners: a systematic review and meta-analysis. Gait Posture.

[166] Van Hooren B, Fuller JT, Buckley JD (2020). Is motorized treadmill running biomechanically comparable to overground running? A systematic review and meta-analysis of cross-over studies. Sports Med.

[167] Tudor-Locke C, Brashear MM, Katzmarzyk PT, Johnson WD (2012). Peak stepping cadence in free-living adults: 2005–2006 NHANES. J Phys Act Health.

[168] Tudor-Locke C, Camhi SM, Leonardi C (2011). Patterns of adult stepping cadence in the 2005–2006 NHANES. Prev Med.

[169] Liu SH, Lin CB, Chen Y, Huang TS, Hsu CY (2019). An EMG patch for the real-time monitoring of muscle-fatigue conditions during exercise. Sensors (Basel).

[170] Lussiana T, Gindre C (2015). Feel your stride and find your preferred running speed. Biol Open.

[171] Zamparo P, Perini R, Peano C, di Prampero PE (2001). The self selected speed of running in recreational long distance runners. Int J Sports Med.

[172] Kong PW, Candelaria NG, Tomaka J (2009). Perception of self-selected running speed is influenced by the treadmill but not footwear. Sports Biomech.

[173] Giandolini M, Horvais N, Rossi J, Millet GY, Samozino P, Morin JB (2016). Foot strike pattern differently affects the axial and transverse components of shock acceleration and attenuation in downhill trail running. J Biomech.

[174] Giandolini M, Pavailler S, Samozino P, Morin JB, Horvais N (2015). Foot strike pattern and impact continuous measurements during a trail running race: proof of concept in a world-class athlete. Footwear Sci.

[175] Vernillo G, Giandolini M, Edwards WB (2017). Biomechanics and physiology of uphill and downhill running. Sports Med.

[176] Jones AM, Doust JH (1996). A 1% treadmill grade most accurately reflects the energetic cost of outdoor running. J Sports Sci.

[177] Gidley AD, Lankford DE, Bailey JP (2020). The construction of common treadmills significantly affects biomechanical and metabolic variables. J Sports Sci.

[178] Smith JAH, McKerrow AD, Kohn TA (2017). Metabolic cost of running is greater on a treadmill with a stiffer running platform. J Sports Sci.

[179] Pind R, Mooses K, Suvi S (2019). Better economy on indoor track compared to treadmill running with 1% inclination. Res Q Exerc Sport.

[180] Schütte KH, Aeles J, De Beéck TO, van der Zwaard BC, Venter R, Vanwanseele B (2016). Surface effects on dynamic stability and loading during outdoor running using wireless trunk accelerometry. Gait Posture.

[181] Dixon SJ, Collop AC, Batt ME (2000). Surface effects on ground reaction forces and lower extremity kinematics in running. Med Sci Sports Exerc.

[182] Riley PO, Dicharry J, Franz J, Della Croce U, Wilder RP, Kerrigan DC (2008). A kinematics and kinetic comparison of overground and treadmill running. Med Sci Sports Exerc.

[183] Koldenhoven RM, Hertel J (2018). Validation of a wearable sensor for measuring running biomechanics. Digit Biomark.

[184] Peake JM, Kerr G, Sullivan JP (2018). A critical review of consumer wearables, mobile applications, and equipment for providing biofeedback, monitoring stress, and sleep in physically active populations. Front Physiol..

[185] Castagna C, Ganzetti M, Ditroilo M, Giovannelli M, Rocchetti sA, Manzi V (2013). Concurrent validity of vertical jump performance assessment systems. J Strength Cond Res.

[186] Glatthorn JF, Gouge S, Nussbaumer S, Stauffacher S, Impellizzeri FM, Maffiuletti NA (2011). Validity and reliability of Optojump photoelectric cells for estimating vertical jump height. J Strength Cond Res.

[187] Paquette MR, Melcher DA (2017). Impact of a long run on injury-related biomechanics with relation to weekly mileage in trained male runners. J Appl Biomech.

[188] Dierks TA, Davis IS, Hamill J (2010). The effects of running in an exerted state on lower extremity kinematics and joint timing. J Biomech.

[189] Maas E, De Bie J, Vanfleteren R, Hoogkamer W, Vanwanseele B (2018). Novice runners show greater changes in kinematics with fatigue compared with competitive runners. Sports Biomech.

[190] Schütte KH, Maas EA, Exadaktylos V, Berckmans D, Venter RE, Vanwanseele B (2015). Wireless tri-axial trunk accelerometry detects deviations in dynamic center of mass motion due to running-induced fatigue. PLoS One.

[191] Ferber R, Davis IM, Williams DS (2003). Gender differences in lower extremity mechanics during running. Clin Biomech (Bristol, Avon).

[192] Phinyomark A, Hettinga BA, Osis ST, Ferber R (2014). Gender and age-related differences in bilateral lower extremity mechanics during treadmill running. PLoS ONE.

[193] Clermont CA, Osis ST, Phinyomark A, Ferber R (2017). Kinematic gait patterns in competitive and recreational runners. J Appl Biomech.

[194] Clermont CA, Phinyomark A, Osis ST, Ferber R (2019). Classification of higher- and lower-mileage runners based on running kinematics. J Sport Health Sci.

[195] Clermont CA, Duffett-Leger L, Hettinga BA, Ferber R (2020). Runners' perspectives on 'smart' wearable technology and its use for preventing injury. Int J Hum Comput Interact.

[196] Davidson P, Virekunnas H, Sharma D, Piché R, Cronin N (2019). Continuous analysis of running mechanics by means of an integrated INS/GPS device.. Sensors (Basel).

[197] Gholami M, Rezaei A, Cuthbert TJ, Napier C, Menon C (2020). Lower body kinematics monitoring in running using fabric-based wearable sensors and deep convolutional neural networks. Sensors (Basel).

[198] Goss DL, Gross MT (2013). A comparison of negative joint work and vertical ground reaction force loading rates in Chi runners and rearfoot-striking runners. J Orthop Sports Phys Ther.

[199] Matijevich ES, Branscombe LM, Scott LR, Zelik KE (2019). Ground reaction force metrics are not strongly correlated with tibial bone load when running across speeds and slopes: Implications for science, sport and wearable tech. PLoS One.

[200] Simoni L, Pancani S, Vannetti F, Macchi C, Pasquini G (2020). Relationship between lower limb kinematics and upper trunk acceleration in recreational runners. J Healthc Eng.

[201] Sinclair J, Goodwin JF, Richards J, Shore H (2016). The influence of minimalist and maximalist footwear on the kinetics and kinematics of running. Footwear Sci.

[202] Sinclair J, Taylor PJ, Hobbs SJ (2014). Kinematic regulation of time and frequency domain components of accelerations measured at the tibia during heel-toe running. Hum Move.

[203] Singh P, Esposito M, Barrons Z, Clermont CA, Wannop J, Stefanyshyn D (2021). Measuring gait velocity and stride length with an ultrawide bandwidth local positioning system and an inertial measurement unittoe running. Sensors (Basel).

[204] Lee Y-S, Ho CS, Shih Y, Chang SY, Róbert FJ, Shiang TY (2015). Assessment of walking, running, and jumping movement features by using the inertial measurement unit. Gait Posture.

[205] Papi E, Spulber I, Kotti M, Georgiou P, McGregor AH (2015). Smart sensing system for combined activity classification and estimation of knee range of motion. IEEE Sens J.

[206] Adams D, Pozzi F, Willy RW, Carrol A, Zeni J (2018). Altering cadence or vertical oscillation during running: effects on running related injury factors. Int J Sports Phys Ther.

[207] Argunsah Bayram H, Yalcin B (2021). The influence of biofeedback on physiological and kinematic variables of treadmill running. Int J Perform Anal Sport.

[208] Chan PPK, Chan ZYS, Au IPH, Lam BMF, Lam WK, Cheung RTH (2021). Biomechanical effects following footstrike pattern modification using wearable sensors. J Sci Med Sport.

[209] Cheung RTH, An WW, Au IPH (2017). Measurement agreement between a newly developed sensing insole and traditional laboratory-based method for footstrike pattern detection in runners. PLoS ONE.

[210] Cheung RTH, An WW, Au IPH, Zhang JH, Chan ZYS, MacPhail AJ (2018). Control of impact loading during distracted running before and after gait retraining in runners. J Sports Sci.

[211] Cheung RTH, Zhang JH, Chan ZYS (2019). Shoe-mounted accelerometers should be used with caution in gait retraining. Scand J Med Sci Sports.

[212] Clarke TE, Cooper LB, Hamill CL, Clark DE (1985). The effect of varied stride rate upon shank deceleration in running. J Sports Sci.

[213] Creaby MW, Franettovich Smith MM (2016). Retraining running gait to reduce tibial loads with clinician or accelerometry guided feedback. J Sci Med Sport.

[214] Crowell HP, Davis IS (2011). Gait retraining to reduce lower extremity loading in runners. Clin Biomech.

[215] Crowell HP, Milner CE, Hamill J, Davis IS (2010). Reducing impact loading during running with the use of real-time feedback. J Orthopaed Sports Phys Ther.

[216] Fuller JT, Thewlis D, Tsiros MD, Brown NAT, Buckley JD (2017). Six-week transition to minimalist shoes improves running economy and time-trial performance. J Sci Med Sport.

[217] Fuller JT, Thewlis D, Tsiros MD, Brown NAT, Hamill J, Buckley JD (2019). Longer-term effects of minimalist shoes on running performance, strength and bone density: a 20-week follow-up study. Eur J Sport Sci.

[218] Gantz AM, Derrick TR (2018). Kinematics and metabolic cost of running on an irregular treadmill surface. J Sports Sci.

[219] Giandolini M, Horvais N, Farges Y, Samozino P, Morin JB (2013). Impact reduction through long-term intervention in recreational runners: midfoot strike pattern versus low-drop/low-heel height footwear. Eur J Appl Physiol.

[220] Gotoda N, Matsuura K, Otsuka S, Tanaka T, Yano Y (2011). Remote coaching system for runner's form with wearable wireless sensor. Int J Mob Learn Org.

[221] Kernozek TW, Vannatta CN, Gheidi N, Kraus S, Aminaka N (2016). Plantar loading changes with alterations in foot strike patterns during a single session in habitual rear foot strike female runners. Phys Ther Sport.

[222] Meinert I, Brown N, Alt W (2016). Effect of footwear modifications on oscillations at the Achilles tendon during running on a treadmill and over ground: a cross-sectional study. Plos One.

[223] Mercer JA, Bezodis NE, Russell M, Mercer A, Mercer D (2005). Kinetic consequences of constraining running behavior. J Sports Sci Med.

[224] Phanpho C, Rao S, Moffat M (2019). Immediate effect of visual, auditory and combined feedback on foot strike pattern. Gait Posture.

[225] Sheerin KR, Reid D, Taylor D, Besier TF (2020). The effectiveness of real-time haptic feedback gait retraining for reducing resultant tibial acceleration with runners. Phys Ther Sport.

[226] Van den Berghe P, Gosseries P, Gerlo J, Lenoir M, Leman M, De Clercq D (2020). Change-point detection of peak tibial acceleration in overground running retraining. Sensors (Basel).

[227] Willy RW, Buchenic L, Rogacki K, Ackerman J, Schmidt A, Willson JD (2016). In-field gait retraining and mobile monitoring to address running biomechanics associated with tibial stress fracture. Scand J Med Sci Sports.

[228] Zhang JH, Chan ZY, Au IP, An WW, Shull PB, Cheung RT (2019). Transfer learning effects of biofeedback running retraining in untrained conditions. Med Sci Sports Exerc.

[229] Macadam P, Cronin JB, Uthoff AM, et al. Thigh loaded wearable resistance increases sagittal plane rotational work of the thigh resulting in slower 50-m sprint times [published correction appears in Sports Biomech. 2021 Feb 22;:1]. Sports Biomech. 2020;1–12. 10.1080/14763141.2020.1762720 32460633

[230] Macadam P, Nuell S, Cronin JB, et al. Load effects of thigh wearable resistance on angular and linear kinematics and kinetics during non-motorised treadmill sprint-running [published correction appears in Eur J Sport Sci. 2021 Mar 29;:1]. Eur J Sport Sci. 2021;21(4):531–38. 10.1080/17461391.2020.1764629 32357805

[231] Mercer JA, Chona C (2015). Stride length–velocity relationship during running with body weight support. J Sport Health Sci.

[232] Moran MF, Rickert BJ, Greer BK (2017). Tibial acceleration and spatiotemporal mechanics in distance runners during reduced-body-weight conditions. J Sport Rehabil.

[233] Ueberschär O, Fleckenstein D, Wüstenfeld JC, Warschun F, Falz R, Wolfarth B (2019). Running on the hypogravity treadmill AlterG^®^ does not reduce the magnitude of peak tibial impact accelerations. Sports Orthopaed Traumatol.

[234] Wundersitz DW, Netto KJ, Aisbett B, Gastin PB (2013). Validity of an upper-body-mounted accelerometer to measure peak vertical and resultant force during running and change-of-direction tasks. Sports Biomech.

[235] Chapman RF, Laymon AS, Wilhite DP, McKenzie JM, Tanner DA, Stager JM (2012). Ground contact time as an indicator of metabolic cost in elite distance runners. Med Sci Sports Exerc.

[236] Hardin EC, Hamill J (2002). The influence of midsole cushioning on mechanical and hematological responses during a prolonged downhill run. Res Q Exerc Sport.

[237] Kaiqiang W, Xingyang L (2002). Wearable pressure sensor for athletes’ full-range motion signal monitoring. Mater Res Express.

[238] McGregor SJ, Busa MA, Yaggie JA, Bollt EM (2002). High resolution MEMS accelerometers to estimate VO_2_ and compare running mechanics between highly trained inter-collegiate and untrained runners. PLoS One.

[239] Meamarbashi A (2013). Quantification of exercise performance intensity during walking and running by three-dimensional accelerometry. Sports Technol.

[240] Leduc C, Tee J, Lacome M (2020). Convergent validity, reliability, and sensitivity of a running test to monitor neuromuscular fatigue. Int J Sports Physiol Perf.

[241] Olcina G, Perez-Sousa MÁ, Escobar-Alvarez JA, Timón R (2019). Effects of cycling on subsequent running performance, stride length, and muscle oxygen saturation in triathletes. Sports (Basel).

[242] Schutte KH, Sackey S, Venter R, Vanwanseele B (2018). Energy cost of running instability evaluated with wearable trunk accelerometry. J Appl Physiol.

[243] Aubry RL, Power GA, Burr JF (2018). An assessment of running power as a training metric for elite and recreational runners. J Strength Cond Res.

[244] Austin CL, Hokanson JF, McGinnis PM, Patrick S (2018). The relationship between running power and running economy in well-trained distance runners. Sports.

[245] Cerezuela-Espejo V, Hernández-Belmonte A, Courel-Ibáñez J, Conesa-Ros E, Martínez-Cava A, Pallarés JG (2020). Running power meters and theoretical models based on laws of physics: effects of environments and running conditions. Physiol Behav.

[246] Encarnación-Martínez A, Sanchis-Sanchis R, Pérez-Soriano P, García-Gallart A. Relationship between muscular extensibility, strength and stability and the transmission of impacts during fatigued running [published online ahead of print, 2020 Aug 24]. Sports Biomech. 2020;1–17. 10.1080/14763141.2020.1797863 32835623

[247] Hoenig T, Hamacher D, Braumann KM, Zech A, Hollander K (2019). Analysis of running stability during 5000 m running. Eur J Sport Sci.

[248] Melo T, Carpes D, Vieira KM (2020). Correlation between running asymmetry, mechanical efficiency, and performance during a 10 km run. Biomech.

[249] Schutte KH, Seerden S, Venter R, Vanwanseele B (2018). Influence of outdoor running fatigue and medial tibial stress syndrome on accelerometer-based loading and stability. Gait Posture.

[250] Blackah N, Bradshaw E, Kemp J, Shoushtarian M (2013). The effect of exercise-induced muscle damage on shock dissipation during treadmill running. Asian J Exerc Sports Sci.

[251] Butler RJ, Davis IM, Laughton CM, Hughes M (2003). Dual-function foot orthosis: effect on shock and control of rearfoot motion. Foot Ankle Int.

[252] Clansey AC, Hanlon M, Wallace ES, Lake MJ (2012). Effects of fatigue on running mechanics associated with tibial stress fracture risk. Medi Sci Sports Exerc.

[253] Derrick TR, Dereu D, McLean SP (2002). Impacts and kinematic adjustments during an exhaustive run. Med Sci Sports Exerc.

[254] Derrick TR, Hamill J, Caldwell GE (1998). Energy absorption of impacts during running at various stride lengths. Med Sci Sports Exerc.

[255] Deriaz O, Najafi B, Ballabeni P (2010). Proximal tibia volumetric bone mineral density is correlated to the magnitude of local acceleration in male long-distance runners. J Appl Physiol.

[256] Dixon SJ, Stiles VH (2003). Impact absorption of tennis shoe-surface combinations. Sports Eng.

[257] Garcia-Perez JA, Pérez-Soriano P, Llana Belloch S, Lucas-Cuevas AG, Sánchez-Zuriaga D (2014). Effects of treadmill running and fatigue on impact acceleration in distance running. Sports Biomech.

[258] Greenhalgh A, Sinclair J, Leat A, Chockalingam N (2012). Influence of footwear choice, velocity and surfaces on tibial accelerations experienced by field hockey participants during running. Footwear Sci.

[259] Horvais N, Samozino P, Chiementin X, Jean-Benoit JB, Giandolini M (2019). Cushioning perception is associated with both tibia acceleration peak and vibration magnitude in heel-toe running. Footwear Sci.

[260] Imbach F, Candau R, Chailan R, Perrey S (2020). Validity of the Stryd power meter in measuring running parameters at submaximal speeds. Sports.

[261] Johnson CD, Outerleys J, Jamison ST, Tenforde AS, Ruder M, Davis IS (2020). Comparison of tibial shock during treadmill and real-world running. Med Sci Sports Exerc.

[262] Lam WK, Liebenberg J, Woo J (2018). Do running speed and shoe cushioning influence impact loading and tibial shock in basketball players?. Peer J.

[263] Laughton CA, Davis IM, Hamill J (2003). Effect of strike pattern and orthotic intervention on tibial shock during running. J Appl Biomech.

[264] Lindsay TR, Yaggie JA, McGregor SJ (2014). Contributions of lower extremity kinematics to trunk accelerations during moderate treadmill running. J Neuroeng Rehabil.

[265] Macdermid PW, Fink PW, Stannard SR (2017). Shock attenuation, spatio-temporal and physiological parameter comparisons between land treadmill and water treadmill running. J Sport Health Sci.

[266] Mercer JA, Bates BT, Dufek JS, Hreljac A (2003). Characteristics of shock attenuation during fatigued running. J Sports Sci.

[267] Mercer JA, Devita P, Derrick TR, Bates BT (2003). Individual effects of stride length and frequency on shock attenuation during running. Med Sci Sports Exerc.

[268] Mercer JA, Vance J, Hreljac A, Hamill J (2002). Relationship between shock attenuation and stride length during running at different velocities. Eur J Appl Physiol.

[269] Milner CE, Ferber R, Pollard CD, Hamill J, Davis IS (2006). Biomechanical factors associated with tibial stress fracture in female runners. Med Sci Sports Exerc.

[270] Milner CE, Hamill J, Davis I (2007). Are knee mechanics during early stance related to tibial stress fracture in runners?. Clin Biomech (Bristol, Avon).

[271] Mizrahi J, Voloshin A, Russek D, Verbitsky O, Isakov E (1997). The influence of fatigue on EMG and impact acceleration in running. Basic Appl Myol.

[272] Ruder M, Jamison ST, Tenforde A, Mulloy F, Davis IS (2019). Relationship of foot strike pattern and landing impacts during a marathon. Med Sci Sports Exerc.

[273] Thompson M, Seegmiller J, McGowan CP (2016). Impact accelerations of barefoot and shod running. Int J Sports Med.

[274] Cher PH, Worringham CJ, Stewart IB (2017). Human runners exhibit a least variable gait speed. J Sports Sci.

[275] Meyer C, Mohr M, Falbriard M, Nigg SR, Nigg BM (2018). Influence of footwear comfort on the variability of running kinematics. Footwear Sci.

[276] M, Chiementin X. Does exhaustion modify acceleration running signature? [published online ahead of print, 2021 Nov 3]. Sports Biomech. 2021;1–11. 10.1080/14763141.2021.1974930 34730472

[277] Rabuffetti M, Scalera GM, Ferrarin M (2019). Effects of gait strategy and speed on regularity of locomotion assessed in healthy subjects using a multi-sensor method. Sensors.

[278] Akenhead R, Marques JB, Paul DJ (2017). Accelerometer load: a new way to measure fatigue during repeated sprint training?. Sci Med Football.

[279] Backes A, Skejø SD, Gette P (2020). Predicting cumulative load during running using field-based measures. Scand J Med Sci Sports.

[280] Rowlands AV, Stiles VH (2012). Accelerometer counts and raw acceleration output in relation to mechanical loading. J Biomech.

[281] Willwacher S, Fischer KM, Brueggemann P (2017). The potential of foot mounted 3D accelerometers to predict lower extremity loading in running. Footwear Sci.

[282] Garcia-Byrne F, Wycherley TP, Bishop C, Schwerdt S, Porter J, Buckley JD (2020). Accelerometer detected lateral sway during a submaximal running test correlates with endurance exercise performance in elite Australian male cricket players. J Sci Med Sport.

[283] Nagano Y, Sasaki S, Higashihara A, Ichikawa H (2016). Gender differences in trunk acceleration and related posture during shuttle run cutting. Int Biomech.

[284] Shull PB, Xu J, Yu B, Zhu X (2017). Magneto-Gyro wearable sensor algorithm for trunk sway estimation during walking and running gait. IEEE Sens J.

[285] Fong DTP, Chan Y, Chu VW, Lam AH, Yung PS (2021). Using a single uniaxial gyroscope to detect lateral ankle sprain hazard. IEEE Sens J.

[286] Havens KL, Cohen SC, Pratt KA, Sigward SM (2018). Accelerations from wearable accelerometers reflect knee loading during running after anterior cruciate ligament reconstruction. Clin Biomech.

[287] Li R, Jumet B, Ren H, Song W, Tse ZTH (2020). An inertial measurement unit tracking system for body movement in comparison with optical tracking. Proc Inst Mech Eng H.

[288] Nagahara R, Kameda M, Neville J, Morin JB (2020). Inertial measurement unit-based hip flexion test as an indicator of sprint performance. J Sports Sci.

[289] Ahamed NU, Kobsar D, Benson L (2019). Using wearable sensors to classify subject-specific running biomechanical gait patterns based on changes in environmental weather conditions. PLoS One.

[290] Ahamed NU, Kobsar D, Benson LC, Clermont CA, Osis ST, Ferber R (2019). Subject-specific and group-based running pattern classification using a single wearable sensor. J Biomech.

[291] Apte S, Meyer F, Gremeaux V, Dadashi F, Aminian K (2020). A sensor fusion approach to the estimation of instantaneous velocity using single wearable sensor during sprint. Front Bioeng Biotechnol.

[292] Aubol KG, Milner CE (2020). Foot contact identification using a single triaxial accelerometer during running. J Biomech.

[293] Benson LC, Clermont CA, Ferber R (2020). New considerations for collecting biomechanical data using wearable sensors: the effect of different running environments. Front Bioeng Biotechnol.

[294] Benson LC, Clermont CA, Osis ST, Kobsar D, Ferber R (2018). Classifying running speed conditions using a single wearable sensor: optimal segmentation and feature extraction methods. J Biomech.

[295] Benson LC, Clermont CA, Watari R, Exley T, Ferber R (2018). Automated accelerometer-based gait event detection during multiple running conditions.. Sensors.

[296] Bergamini E, Picerno P, Pillet H, Natta F, Thoreux P, Camomilla V (2012). Estimation of temporal parameters during sprint running using a trunk-mounted inertial measurement unit. J Biomech.

[297] Billing DC, Nagarajah R, Hayes J, Baker JD (2006). Predicting ground reaction forces in running using micro-sensors and neural networks. Sports Eng.

[298] Cooper G, Sheret I, McMillan L (2009). Inertial sensor-based knee flexion/extension angle estimation. J Biomech.

[299] Derie R, Robberechts P, Van den Berghe P (2020). Tibial acceleration-based prediction of maximal vertical loading rate during overground running: a machine learning approach. Front Bioeng Biotechnol.

[300] Dorschky E, Nitschke M, Seifer AK, van den Bogert AJ, Eskofier BM (2019). Estimation of gait kinematics and kinetics from inertial sensor data using optimal control of musculoskeletal models. J Biomech.

[301] Falbriard M, Meyer F, Mariani B, Millet GP, Aminian K (2018). Accurate estimation of running temporal parameters using foot-worn inertial sensors. Front Physiol.

[302] Falbriard M, Meyer F, Mariani B, Millet GP, Aminian K (2020). Drift-free foot orientation estimation in running using wearable IMU. Front Bioeng Biotechnol.

[303] Gurchiek RD, McGinnis RS, Needle AR, McBride JM, van Werkhoven H (2018). An adaptive filtering algorithm to estimate sprint velocity using a single inertial sensor. Sports Eng.

[304] Hernandez V, Dadkhah D, Babakeshizadeh V, Kuli D (2021). Lower body kinematics estimation from wearable sensors for walking and running: a deep learning approach. Gait Posture.

[305] Huang YC, Chen YR, WU HY, Huang YJ (2019). Wearable sensor for measurement of gait walking and running motion. Sens Mater.

[306] Johnson WR, Mian A, Robinson MA, Verheul J, Lloyd DG, Alderson JA (2021). Multidimensional ground reaction forces and moments from wearable sensor accelerations via deep learning. IEEE Trans Biomed Eng.

[307] Khandelwal S, Wickstrom N (2017). Evaluation of the performance of accelerometer-based gait event detection algorithms in different real-world scenarios using the MAREA gait database. Gait Posture.

[308] Kim J, Bae MN, Lee KB, Hong SG (2020). Gait event detection algorithm based on smart insoles. ETRI J.

[309] LeBlanc J, Hernandez EM, McGinnis RS, Gurchiek RD (2021). Continuous estimation of ground reaction force during long distance running within a fatigue monitoring framework: A Kalman filter-based model-data fusion approach. J Biomech.

[310] Li F, Liu G, Liu J, Chen X, Ma X (2016). 3D Tracking via Shoe Sensing. Sensors (Basel).

[311] Liu Q, Liu S, Cheung VCK (2020). Classification of runners' performance levels with concurrent prediction of biomechanical parameters using data from inertial measurement units. J Biomech.

[312] Mannini A, Sabatini AM (2012). Gait phase detection and discrimination between walking-jogging activities using hidden Markov models applied to foot motion data from a gyroscope. Gait Posture.

[313] Matijevich ES, Scott LR, Volgyesi P, Derry KH, Zelik KE (2020). Combining wearable sensor signals, machine learning and biomechanics to estimate tibial bone force and damage during running. Hum Mov Sci.

[314] Mitschke C, Zaumseil F, Milani TL (2017). The influence of inertial sensor sampling frequency on the accuracy of measurement parameters in rearfoot running. Comput Methods Biomech Biomed Eng.

[315] Mo L, Zeng L. Running gait pattern recognition based on cross-correlation analysis of single acceleration sensor. Math Biosci Eng. 2019;16(6):6242–56. 10.3934/mbe.2019311 31698560

[316] Moore SR, Kranzinger C, Fritz J, Stӧggl T, Krӧll J, Schwameder H (2020). Foot strike angle prediction and pattern classification using loadsolTM wearable sensors: a comparison of machine learning techniques. Sensors (Basel).

[317] Neville JG, Rowlands D, Lee J, James DA (2016). A model for comparing over-ground running speed and accelerometer derived step rate in elite level athletes. IEEE Sens J.

[318] Neugebauer JM, Collins KH, Hawkins DA (2014). Ground reaction force estimates from ActiGraph GT3X+Hip accelerations. PloS One.

[319] Nüesch C, Roos E, Egloff C, Pagenstert G, Mündermann A (2019). The effect of different running shoes on treadmill running mechanics and muscle activity assessed using statistical parametric mapping (SPM). Gait Posture.

[320] Parshad RD, McGregor SJ, Busa MA, Skufca JD, Bollt E (2012). A statistical approach to the use of control entropy identifies differences in constraints of gait in highly trained versus untrained runners. Math Biosci Eng.

[321] Pogson M, Verheul J, Robinson MA, Vanrenterghem J, Lisboa P (2020). A neural network method to predict task- and step-specific ground reaction force magnitudes from trunk accelerations during running activities. Med Eng Phys.

[322] Potter MV, Ojeda LV, Perkins NC, Cain SM (2019). Effect of IMU design on IMU-derived stride metrics for running. Sensors (Basel).

[323] Rapp E, Shin S, Thomsen W, Ferber R, Halilaj E (2021). Estimation of kinematics from inertial measurement units using a combined deep learning and optimization framework. J Biomech.

[324] Robberechts P, Derie R, Van den Berghe P (2021). Predicting gait events from tibial acceleration in rearfoot running: a structured machine learning approach. Gait Posture.

[325] Stetter BJ, Krafft FC, Ringhof S, Stein T, Sell S (2020). A machine learning and wearable sensor based approach to estimate external knee flexion and adduction moments during various locomotion tasks. Front Bioeng Biotechnol.

[326] Stetter BJ, Ringhof S, Krafft FC, Sell S, Stein T (2019). Estimation of knee joint forces in sport movements using wearable sensors and machine learning. Sensors (Basel).

[327] Sui J, Chang T (2021). IMU based deep stride length estimation with self-supervised learning. IEEE Sens J.

[328] Tan HX, Aung NN, Tian J, Chua MCH, Yang YO (2019). Time series classification using a modified LSTM approach from accelerometer-based data: a comparative study for gait cycle detection. Gait Posture.

[329] van Oeveren BT, de Ruiter CJ, Beek PJ, Rispens SM, van Dieën JH (2018). An adaptive, real-time cadence algorithm for unconstrained sensor placement. Med Eng Phys.

[330] Yang S, Mohr C, Li Q (2011). Ambulatory running speed estimation using an inertial sensor. Gait Posture.

[331] Zrenner M, Gradl S, Jensen U, Ullrich M, Eskofier BM (2018). Comparison of different algorithms for calculating velocity and stride length in running using inertial measurement units. Sensors.

[332] Nazarahari M, Khandan A, Khan A, Rouhani H (2022). Foot angular kinematics measured with inertial measurement units: A reliable criterion for real-time gait event detection. Sensors.

[333] Einicke GA, Sabti HA, Thiel DV (2018). Maximum-entropy-rate selection of features for classifying changes in knee and ankle dynamics during running. IEEE J Biomed Health Inform.

[334] McGregor SJ, Busa MA, Skufca J, Yaggie JA, Bollt EM (2009). Control entropy identifies differential changes in complexity of walking and running gait patterns with increasing speed in highly trained runners. Chaos.

[335] De la Fuente C, Henríquez H, Andrade DC, Yañez A. Running footwear with custom insoles for pressure distribution are appropriate to diminish impacts after shin splints. Asian J Sports Med. 2019;10(3).

[336] Woodward MI, Cunningham JL (1993). Skeletal accelerations measured during different exercises. Proc Inst Mech Eng H.

[337] Wu G, Ladin Z (1996). The study of kinematic transients in locomotion using the integrated kinematic sensor. IEEE Trans Rehabil Eng.

[338] Yingling VR, Yack HJ, White SC (1996). The effect of rearfoot motion on attenuation of the impulse wave at impact during running. J Appl Biomech.

[339] Asmussen MJ, Kaltenbach C, Hashlamoun K, Shen H, Federico S, Nigg BM (2019). Force measurements during running on different instrumented treadmills. J Biomech.

[340] Bigelow EM, Elvin NG, Elvin AA, Arnoczky SP (2013). Peak impact accelerations during track and treadmill running. J Appl Biomech.

[341] Di Paolo S, Lopomo NF, Della Villa F (2013). Rehabilitation and return to sport assessment after anterior cruciate ligament injury: quantifying joint kinematics during complex high-speed tasks through wearable sensors. Sensors.

[342] Hennig EM, Lafortune MA (1991). Relationships between ground reaction force and tibial bone acceleration parameters. Int J Sport Biomech.

[343] Linkis JE, Bonne TC, Bejder J, Rasmussen EK, Breenfeldt Andersen A, Nordsborg NB (2021). Reliability and validity of the SHFT running power meter. Sensors (Basel).

[344] Lucas-Cuevas AG, Encarnación-Martínez A, Camacho-García A, Llana-Belloch S, Pérez-Soriano P (2017). The location of the tibial accelerometer does influence impact acceleration parameters during running. J Sports Sci.

[345] Rowlands AV, Schuna JM, Stiles VH, Tudor-Locke C (2014). Cadence, peak vertical acceleration, and peak loading rate during ambulatory activities: implications for activity prescription for bone health. J Phys Act Health.

[346] Santos-Lozano A, Torres-Luque G, Marín PJ, Ruiz JR, Lucia A, Garatachea N (2012). Intermonitor variability of GT3X accelerometer. In J Sports Med.

[347] Willis SJ, Gellaerts J, Mariani B, Basset P, Borrani F, Millet GP (2019). Level versus uphill economy and mechanical responses in elite ultratrail runners. Int J Sports Physiol Perform.

[348] Zhang H, Zanotto D, Agrawal SK (2017). Estimating CoP trajectories and kinematic gait parameters in walking and running using instrumented insoles. IEEE Robot Autom Lett.

[349] Waite N, Goetschius J, Lauver JD (2021). Effect of grade and surface type on peak tibial acceleration in trained distance runners. J Appl Biomech.

[350] van den Tillaar R, Nagahara R, Gleadhill S, Jiménez-Reyes P (2021). Step-to-step kinematic validation between an Inertial Measurement Unit (IMU) 3D system, a combined Laser+IMU system and force plates during a 50 M sprint in a cohort of sprinters. Sensors (Basel).

[351] Li M, Torah R, Nunes-Matos H (2020). Integration and testing of a three-axis accelerometer in a woven e-textile sleeve for wearable movement monitoring. Sensors (Switzerland).

[352] Liu S, Zhang J, Zhang Y, Zhu R (2020). A wearable motion capture device able to detect dynamic motion of human limbs. Nat Commun..

[353] Low JH, Chee PS, Lim EH, Ganesan V (2020). Design of a wireless smart insole using stretchable microfluidic sensor for gait monitoring. Smart Mater Struct.

[354] Provot T, Chiementin X, Oudin E, Bolaers F, Murer S (2017). Validation of a high sampling rate inertial measurement unit for acceleration during running. Sensors (Basel).

[355] Tan H, Wilson AM, Lowe J (2008). Measurement of stride parameters using a wearable GPS and inertial measurement unit. J Biomech.

[356] Yuan Q, Chen IM (2012). Human velocity and dynamic behavior tracking method for inertial capture system. Sens Actuators A Phys.

[357] Yuan Q, Chen IM (2014). Localization and velocity tracking of human via 3 IMU sensors. Sens Actuators A Phys.

[358] Lin Z, Wu IZ, Zhang B, Wang YC, Guo H, Liu G, Chen C, Chen Y, Yang J, Wang ZL (2019). A triboelectric nanogenerator-based smart insole for multifunctional gait monitoring. Adv Mater Technol.

[359] Liu Q, Williamson J, Li K, Mohrman W, Lv Q, Dick RP, Shang L (2017). Gazelle: energy-efficient wearable analysis for running. IEEE Transact Mob Comput.

[360] Park J, Kim SJ, Na Y, Kim Y, Kim J (2019). Development of a bendable outsole biaxial ground reaction force measurement system. Sensors (Basel).

[361] Xinan L, Hongyuan X, Cheung JT (2016). Gait-force model and inertial measurement unit-based measurements: a new approach for gait analysis and balance monitoring. J Exerc Sci Fit.

[362] Armitage M, Beato M, McErlain-Naylor SA (2021). Inter-unit reliability of IMU step metrics using IMeasureU Blue Trident inertial measurement units for running-based team sport tasks. J Sports Sci.

[363] Colapietro M, Fraser JJ, Resch JE, Hertel J (2020). Running mechanics during 1600 meter track runs in young adults with and without chronic ankle instability. Phys Ther Sport.

[364] Hughes T, Jones RK, Starbuck C, Sergeant JC, Callaghan MJ (2019). The value of tibial mounted inertial measurement units to quantify running kinetics in elite football (soccer) players: a reliability and agreement study using a research orientated and a clinically orientated system. J Electromyogr Kinesiol.

[365] Kiernan D, Hawkins DA, Manoukian MAC (2018). Accelerometer-based prediction of running injury in National Collegiate Athletic Association track athletes. J Biomech.

[366] Le Bris R, Billat V, Auvinet B, Chaleil D, Hamard L, Barrey E (2006). Effect of fatigue on stride pattern continuously measured by an accelerometric gait recorder in middle distance runners. J Sports Med Phys Fit.

[367] Ueberschär O, Fleckenstein D, Warschun F, Kränzler S, Walter N, Hoppe MW (2019). Measuring biomechanical loads and asymmetries in junior elite long-distance runners through triaxial inertial sensors. Sports Orthopaed Traumatol.

[368] Young F, Coulby G, Watson I, Downs C, Stuart S, Godfrey A (2020). Just find it: the Mymo approach to recommend running shoes. IEEE Access.

[369] Jidovtseff B, Rodriguez de la Cruz C, Croisier JL, Maquet D, Bury T, Deflandre D (2019). Influence of fatigue on stride biomechanical parameters measured by an accelerometer. Sci Sports.

[370] Andersen C, Nielsen M. Reliability and validity of Garmin Forerunner 735XT for measuring running dynamics in-field. 2017.

[371] Deflandre D, Miny K, Scwartz C, Dardenne N, Leclerc AF, Bury T (2018). Myotest efficiency in the mechanical analysis of the stride. Gazzetta Med Ital Arch Sci Mediche..

[372] Lafortune MA, Hennig EM (1991). Contribution of angular motion and gravity to tibial acceleration. Med Sci Sports Exerc.

