# Supplementary material for: Wearables for Running Gait Analysis: A Systematic Review
Source: Sports Med. 2022 Oct 15;53(1):241–68. doi: 10.1007/s40279-022-01760-6 (PMC9807497; doi:10.1007/s40279-022-01760-6)
Supplement: Supplementary file 1 — Supplementary file1 (DOCX 121 KB) [file 40279_2022_1760_MOESM1_ESM.docx]

**Wearables for Running Gait Analysis: A Systematic Review**

Rachel Mason^1^, Liam T Pearson^1^, Gillian Barry^1^, Fraser Young^3^, Oisin Lennon^2^, Alan Godfrey^3^ & Samuel Stuart^1,4^*

1. *Department of Sport, Exercise and Rehabilitation, Northumbria University, UK.*
2. *DANU Sports Ltd., Ireland*
3. *Department of Computer and Information Sciences, Northumbria University, UK*
4. *Northumbria Healthcare NHS foundation trust, UK*

**Corresponding Author:* [*sam.stuart@northumbria.ac.uk*](mailto:sam.stuart@northumbria.ac.uk)

**Supplementary Table 1** Reasons for why papers were excluded from the review

| Exclusion Reason: |  |
| --- | --- |
| Does not use wearable technology to assess running gait | [201, 202] [203]* [204-208] |
| Movement classification | [209, 210] |
| Gait retraining | [211-213] [214]* [215-221] [222]* [223-233] |
| Altered weight conditions | [234-238] |
| Change of direction | [239] |
| Estimation of physiological parameters | [240-247] |
| Running power/stability/economy | [185, 248-254] |
| Tibial shock/impact absorption/vibration/stiffness | [178, 255-264] [265]* [266-278] |
| Gait variability/regularity | [195, 279-282] |
| Load | [283-286] |
| Trunk sway | [287-289] |
| Not focused on running gait measures | [290-293] |
| Algorithm development/machine learning/statistical modelling | [100, 294-337] |
| Control entropy | [338, 339] |
| Focus on raw signals | [340-343] |
| Communication/brief report/technical note/letter | [344-355] |
| Case study | [356-362] |
| No/unclear protocol | [363-366] |
| Inclusion of < 18-year-olds | [367]* [368-373] |
| Paper not written in English | [374] |
| No access to paper | [375]* [376, 377] |
| * denotes that the reference was additionally sourced | |
