# Supplementary material for: Wearables for Running Gait Analysis: A Systematic Review
Source: Sports Med. 2022 Oct 15;53(1):241–68. doi: 10.1007/s40279-022-01760-6 (PMC9807497; doi:10.1007/s40279-022-01760-6)
Supplement: Supplementary file 2 — Supplementary file2 (DOCX 306 KB) [file 40279_2022_1760_MOESM2_ESM.docx]

**Wearables for Running Gait Analysis: A Systematic Review**

Rachel Mason^1^, Liam T Pearson^1^, Gillian Barry^1^, Fraser Young^3^, Oisin Lennon^2^, Alan Godfrey^3^ & Samuel Stuart^1,4^*

1. *Department of Sport, Exercise and Rehabilitation, Northumbria University, UK.*
2. *DANU Sports Ltd., Ireland*
3. *Department of Computer and Information Sciences, Northumbria University, UK*
4. *Northumbria Healthcare NHS foundation trust, UK*

**Corresponding Author:* [*sam.stuart@northumbria.ac.uk*](mailto:sam.stuart@northumbria.ac.uk)

**Supplementary Table 2** Extracted information for all included references

| Reference | Aim | Participants  Age (years)  Height (cm)  Weight (kg) | Inclusion Criteria | Wearable Technology | Protocol | Reference Measures | Outcomes | Main Findings |
| --- | --- | --- | --- | --- | --- | --- | --- | --- |
| *Validity and/or reliability* | |  |  |  |  |  |  |  |
| *Inertial Measurement Units* | |  |  |  |  |  |  |  |
| Ammann, Taube and Wyss (2016)  [99] | To validate the detection of GCT during running in two differently working systems: a small inertial measurement sensor, PARTwear, and the optical measurement system, Optojump, placed on the track. | 12 (7M, 5F)  **Age:** 25.3 ± 3.2  **Height:** 174.4 ± 7.9  **Weight:** 64.8 ± 10.2 | Healthy  High-level running athletes | **IMU (3D Accelerometer, 3D Gyroscope, 3D Magnetometer):** PARTwear  **SF:** 1000Hz  **Accelerometer Range:** 16g  **Size:** 3.8 x 3.7 x 0.8cm  **Weight:** 13g  **Number:** 2  **Location:** Shoelaces | **Environment:** Indoor (Track)  **Speed**: Self-selected (max. sprint, intense, training speed)  **Distance/Time**: 3 (sets) x 4 (runs) x 1 step (30m from start of 40m run)  **Shoe:** Own | High Speed Video Camera System (MarathonUltra). Optojump | GCT | Compared to the high-speed camera, the PartWear and the Optojump systems underestimated GCT by -1.3 ± 6.1% and -16.5 ± 6.7% (p < 0.05), respectively.  The ICCs between PartWear and high-speed camera and between optojump and high-speed camera were 0.984 and 0.853 (p < .001), respectively. Despite the constant systematic underestimation of GCT, analyses indicated that PartWear successfully recorded GCT over a wide range of speeds. |
| Blauberger, Horsch and Lames (2021)  [86] | To validate the detection of GCTs for elite sprinters in the field with shoe-mounted IMUs | 5 (3M, 2F)  **Age:** 22.6 ± 2.7  **Weight:** 69.6 ± 11.5 | Elite national sprinters | **IMU (3D Accelerometer, 3D Gyroscope):** Physilog5, Gait Up SA  **SF:** 512Hz  **Accelerometer Range:** 16g  **Gyroscope Range:** 2000°/s  **Size:** 4.75 x 2.65 x 1.0cm  **Weight:** 11g  **Number:** 2  **Location:** Shoe (above ankle) | **Environment:** Outdoor (Track)  **Speed:** Self-selected (Max. effort)  **Distance/Time:** 34 x 50m & 100m (50 steps from each run, total 1140 steps analysed) | Photoelectric bars (Optogait, Microgate) | GCT | 97.08% steps were detected correctly. R elative time difference between IMU- and Optogait-GCT was 3.55 ± 6.16 ms. RMSE of GCT was 7.97 ms. Error analyses showed that GCT at the beginning and the end of the sprint was classified with smaller errors. |
| Chew et al. (2018)  [92] | Proposes a new gait event identification method, which uses acceleration of the foot to determine FS and FO. Also aims to demonstrate that spatial parameters, can be estimated accurately using the method. | 10 (M)  **Age:** 25.5 ± 3.8  **Height:** 174.4 ± 19.5  **Weight:** 65.5 ± 15.2 |  | **IMU:** Opal, APDM Inc  **SF:** 128Hz  **Accelerometer Range:** 6g  **Gyroscope Range:** 2000°/s  **Size:** 3.65 x 3.61 x 1.34cm  **Weight:** 550g  **Number:** 1  **Location:** Shoe (right foot, 3rd metatarsal) | **Environment:** Indoor (Treadmill)  **Speed:** Controlled (8, 9, 10 and 11 km/hr)  **Distance/Time:** 2 (sessions) x 2 (speeds, speeds different each session) x 3 mins. (Additional 4 x 3 mins walking) | Motion capture (Qualisys, 128Hz) | GCT  Stride length  Stride time  FT  Gait events  Speed | Information derived from a shoe-mounted IMU can be used to estimate the timing of foot-strike and foot-off, the duration of stride, stance phase and swing phase, stride length and running speed. Average temporal differences between the IMU and force plate are between - 2.60 and 3.04 ms for foot-strike, and - 4.34 and 16.28 ms for foot-off. No statistical difference was found in the duration of stride, stance phase and swing phase. Stride length and speed were estimated with average errors between 14.07 and 45.97 cm and between 0.08 and 0.41 m/s, respectively. |
| Day et al. (2021)  [24] | Investigate the effects of varying low-pass filter cutoff frequency on the correlation of IMU-derived peak vGRF and GCT to gold-standard lab-based measurements | 30 (9M, 21F)  **Weight:** M: 63.6 ± 6.7, F: 54.0 ± 5.3 | National Collegiate Athletics Association Division 1 cross country runners | **IMU (3D Accelerometer):** IMeasureU  **SF:** 500Hz  **Accelerometer Range:** 16g  **Number:** 1  **Location:** Lower back (Sacrum) | **Environment:** Indoor (Treadmill)  **Speed:** Controlled (M: 3.8, 4.1, & 5.4 m/s, F: 3.8 & 4.9 m/s)  **Distance/Time:** 2 or 3 (speeds) x 10s (20-30 steps) | Instrumented Treadmill (Treadmetrix, 1000Hz) | GCT  vGRF | Correlations between IMU-derived peak vGRF and GCT and gold standard values measured from the instrumented treadmill varied from very weak to moderate. For peak GRFv, the 10 Hz lowpass  filter cutoff performed best (r = 0.638), while for GCT the 5 Hz cut-off performed best (r = 0.656). IMU-derived estimates of force and contact time are influenced by the lowpass filter cutoff frequency |
| DeJong and Hertel (2020)  [93] | Assess the validity of five wearable sensors (Moov Now, MilestonePod, RunScribe, TgForce and Zoi) to measure GRF related metrics, step rate, FSP, and vertical displacement of the COM during running | 32 (19M, 13F)  **Age:** 27.0 ± 5.5  **Height:** 174.4 ± 8.5  **Weight:** 69.1 ± 11.4 | Healthy  Physically active  Aged 18-45 years  **Exclusion:** Lower extremity musculoskeletal injury or surgery < 2 years prior  Neurological disorder | **IMU:** Moov Now - Moov, MilestonePod - Milestone Sports, RunScribe -RunScribe Labs, TgForce, -Runteq, Zoi - Kelsec Systems  **Weight:** 15.1, 13.0, 15.0, 12.0, 10.0g  **Number:** 1 or 2  **Location:** Chest, Tibia, Ankle, Shoe | **Environment:** Indoor (Treadmill)  **Distance/Time:** Averaged over 60s | 3D Motion Capture (9-camera VICON Nexus, 200Hz)  Instrumented Treadmill (Bertec, 1000Hz) | Moov: SF  Impact.  Milestone Pod: SF  FSP  RunScribe: SF  FSP  Shock  Impact G’s  Braking G's  Zol: SF  Impact  Braking  Bouncing  TgForce: SF  Max vertical oscillation  Max sagittal acceleration  Max vector acceleration.  **Additional:**  SF  FSP  FSA  AVLR  IVLR  Peak braking force | SF measurement provided by all wearable sensors was valid (all r>0.96 and p<0.001). Only Zoi provided valid vertical displacement of the COM (r=0.81, p<0.001); only TgForce provided meaningful estimates of instantaneous vertical loading rate (r=0.76, p<0.001); only MilestonePod could discriminate between a RFS, MFS, FFS patterns during running (p<0.001). None of the wearable sensors was valid for estimating peak braking force. |
| DeJong and Hertel (2020)  [81] | Assess the relationship between foot-strike categorization and FSA during running to validate the sensor-derived foot-strike outcome. | 20 (8M, 12F)  **Age:**  **M:** 20.0 ± 2.0, **F:** 20.0 ± 2.0  **Height:**  **M:** 176.5 ± 5.5, **F:** 166.8 ± 5.1  **Weight:**  **M:** 66.8 ± 7.2, **F:** 57.9 ± 7.8 | Healthy  Cross-country athletes, participating invarsity cross-country practices  Free of any lower extremity musculoskeletal injuries | **IMU (3D Accelerometer, Gyroscope):** RunScribe Plus, RunScribe Labs  **SF:** 200Hz  **Number:** 2  **Location:** Shoe (Dorsum) | **Environment:**  Indoor (Treadmill)  **Speed:** Controlled; (2.68, 3.60 m/s)  **Distance/Time:** 2 (speeds) x 3 (trials) x 20 steps, (10 left, 10 right (30s))  **Shoe:** Own | 3D Motion Capture (12 cameras, Vicon Motion System Inc, Model 1.8.5, 250Hz)  Instrumented Treadmill (Bertec Corp, Model 1.0.1, 1000Hz)  Fsa detection (MotionMonitor software, v 9.32) | FSP  **Additional:**  GRF  FSA | A strong, inverse correlation between FSPs and FSA was present (r = -0.86, p <.001). The sensors demonstrated 78% accuracy (RFS = 72.5%, MFS = 55.3%, FFS = 95.4%). Results support the concurrent validity of the sensor-derived FSP. |
| de Ruiter and van Dieen (2019)  [100] | Proposes a simple method to estimate step length during maximal velocity sprints including the acceleration phase. | 16 (13M, 3F)  **Age:** 20.0 ± 3.1  **Height:** 183.0 ± 8.0  **Weight:** 74.1 ± 9.2 | Healthy  Track and field athletes, trained 4-6x/week, performed sprint exercises as part of their regular training schedule | **IMU (3D Accelerometer, 3D Gyroscope, 3D Magnetometer):** MPU-9150, Invensense  **SF:** 500Hz  **Accelerometer Range:** 16g  **Gyroscope Range:** 2000°/s  **Size:** 3.5 x 2.5 x 1.0cm  **Weight:** 11g  **Number:** 2  **Location:** Shoe (Instep) | **Environment:** Indoor (Track)  **Speed:** Self-selected (max. effort)  **Distance/Time:** 2 x first 20 strides of 100% effort 60m sprints (Protocol: 60m sprint @ 60%, 80%, 100%, 60%, 80%, and 100% effort) | Video analysis (10 cameras, Fujifilm XP60 Full-HD, 30Hz)  Custom-made mechanical timing gates equipped with IMUs | SL  Stride length  ST  Stride velocity | The comparison of estimated SLs to reference measurements showed no significant difference (p > 0.05) and acceptable agreement (RMSE = 8.0 cm, bias ± LoA = -0.15 ±16 cm). 30-m and 60-m times in combination with IMU-derived step timing suffice to obtain good estimates of stride and SL during maximal sprint acceleration. |
| de Ruiter et al. (2016)  [79] | Establish the relationships between speed and contact time at different days during short (125m) stretches, run outdoors on tarmac at different (but constant) speeds. Investigate how accurate speed and small changes thereof can be predicted from previous established relations between speed and contact time. | 14 (8M, 6F)  **Age:** 25.0 ± 7.0  **Height:** 182.0 ± 9.0  **Weight:** 71.0 ± 9.1 | Healthy  Recreationally active in various sports | **IMU (3D Accelerometer, 3D Gyroscope):** MPU-9150, Invensense  **SF:** 500Hz  **Number:** 2  **Location:** Shoe (Instep) | **Environment:** Outdoor (Concrete)  **Speed:** Self-selected (40, 50, 20, 80, 50 and again 40% of their self-estimated maximum running intensity)  **Distance/Time:** 2 (sessions) x 4km (analysed as 32 x 125m), calibration runs 12 x 125m intervals (no stops)  **Shoe:** Own | GPS (Garmin Forerunner 620 sports watch, 1Hz to measure speed) | GCT  Speed | Strong individual relations between running speed and CT during outdoor over ground running on flat terrain. The GCT-speed relations were highly reproducible within individuals despite varying weather conditions. During 4km runs, speed estimates based on GCT and GPS were found to be comparable. Estimate speed from GCT measured with inertial sensors on the feet can predict (changes in) running speed quite accurately and may be used instead of or in addition to GPS. |
| de Ruiter et al. (2022)  [105] | Investigate the concurrent validity of IMUs and high-speed video for sagittal plane kinematics during overground sprinting | 16 (10M, 6F)  **Age:** 21.9 ± 2.8  **Height:** 178.0 ± 7.0  **Weight:** 70.1 ± 11.8 | Sprint exercises part of their regular training routine  Free from injury  Abstained from heavy exercise, alcohol and caffeine 24 h before the experiment | **IMU (3D Accelerometer, 3D Gyroscope, 3D Magnetometer):** MPU-9150, Invensense  **SF:** 500Hz  **Size:** 3.5 x 2.5 x 1.0cm  **Weight:** 11g  **Number:** 9  **Location:** Upper back (Th4-Th5), lower back (sacrum), thigh **(**iliotibial tract), tibia, shoe (Instep) | **Environment:** Outdoor (Track or artificial turf)  **Speed:** Self-selected (85, 90, 95 and 100% Max. effort)  **Distance/Time:** 7 (85, 90, 95 and 100% of maximal effort, and maximal fatiguing sprints numbers 1, 3, and 5) x 60m  **Shoe:** Own | 2D Video Analysis (iPhone and 3 x Casio Exilim EX-ZR1000 HS, shutter speed 1/250, frame rate of 240 Hz)  Photoelectric bars (Optogait, Microgate) | GCT  SF  SL  FT  Hip kinematics  Knee kinematics  Trunk kinematics | Coefficients of multiple correlation with video were >0.99 for angles and angular velocities of the thigh and shank but low for the pelvis and trunk (0.13–0.66). For the limb segment angles absolute biases (LoA) were ≤2.9° (≤7.7°) and for angular velocities the values were ≤57°.s−1(≤93°.s−1). |
| García-Pinillos et al. (2020)  [101] | To examine the influence of RunScribe location (i.e., lace shoe vs heel shoe) on the accuracy of spatiotemporal gait characteristics during running by comparing data with a high-speed video analysis system at 1000 Hz | 49 (44M, 5F)  **Age:** 26.0 ± 8.0  **Height:** 174.0 ± 7.0 **Weight:** 71.0 ± 10.0 | Healthy  Physically active and recreationally trained endurance runners (2–3 sessions per week, 10km trial <50min)  > 18 years  Free of injury | **IMU (3D Accelerometer, 3D Gyroscope, 3D Magnetometer)**: RunScribe – ScribeLab  **SF:** 500Hz  **Size:** 3.5 x 2.5 x 7.5cm  **Weight:** 15g  **Number:** 2  **Location:** Shoe (right shoelaces, left heel of shoe) | **Environment:** Indoor (Treadmill)  **Speed:** Self-selected; (Comfortable)  **Gradient:** 0%  **Distance/Time:** 1 x 3 min (3 min total; Mean values were calculated for spatiotemporal parameters from the 3 min recorded, and a 30-s window from 1:30 to 2:00 min was analyzed. Also, 60 s at the beginning and 60s at the end)  **Shoe:** Own | High-speed video analysis (Imaging Source DFK, 33UX174; The Imaging Source Europe GmbH, 1000Hz) | GCT  SF  SL  Step Time  FT | RunScribe is a valid system to measure spatiotemporal parameters during running on a treadmill according to a high-speed video analysis. The location of the RunScribe system plays an important role on the accuracy of spatiotemporal parameters. The shoelace placement showed smaller systematic bias, random errors, and narrower limits of agreement for GCT, FT, and SL, whereas the heel shoe placement was slightly more accurate for the SF. |
| García-Pinillos et al. (2019)  [128] | To evaluate the concurrent validity of two different inertial measurement units for measuring spatiotemporal parameters during running on a treadmill, by comparing data with a high-speed video analysis at 1000 Hz. | 49 (44M, 5F)  **Age:** 26.0 ± 8.0  **Height:** 174.0 ± 7.0  **Weight:** 71.0 ± 10.0 | Healthy  Physically active and recreationally trained endurance runners (10km trial < 50min)  > 18 years  Free of injury | **IMU (Stryd: 3D Accelerometer, 3D Gyroscope. RunScribe: 3D Accelerometer, 3D Gyroscope, 3D magnetometer)**: Stryd Powermeter – Stryd Inc,, RunScribe- ScribeLabs  **SF:** RunScribe: 500Hz  **Weight:** Stryd -9.1g  RunScribe- 15g  **Number:** 2  **Location:** Shoelaces (right) | **Environment:** Indoor (Treadmill)  **Speed:** Self-selected (comfortable)  **Gradient:** 0%  **Distance/Time:** 3 mins (and a 30 s window, from 1:30 to 2:00 min, was analysed)  **Shoe:** Own | High-speed video analysis (Imaging Source DFK, 33UX174; The Imaging Source Europe GmbH, 1000Hz) | GCT  SF  SL  Step Time  FT | Stryd™ system underestimated GCT (5.2%, p < 0.001) and overestimated FT (15.1%, p < 0.001) compared to video analysis; whereas the RunScribe™ system underestimated GCT (2.3%, p = 0.009). No significant differences were observed in SF and SL between the wearable devices and video analysis, the RunScribe™ seems to be a more accurate system for measuring temporal parameters and SL than the Stryd™ system. |
| Gholami, Napier and Menon (2020)  [73] | To investigate the performance of a single shoe-mounted accelerometer to monitor lower extremity running kinematics in the sagittal plane and reduce the degrees of freedom of the IMU. | 10 (M)  **Age:** 27.0 ± 4.0  **Height**: 177.0 ± 7.0  **Weight:** 72.0 ± 7.0 | Healthy | **IMU (3D Accelerometer):** Xsens, Xsen MTw  **SF:** 100Hz  **Number:** 1  **Location:** Shoe | **Environment:** Indoor (Treadmill)  **Speed:** Controlled (8, 9, 10, 11, and 12 km/hr)  **Distance/Time:** 3 (trials) x 5 (speeds) x 60s | 3D Motion Capture (6 camera Vicon System, 100Hz) | Acceleration  Peak hip flexion/extension angle  Peak knee flexion angle during stance phase  Peak ankle plantar/dorsiflexion angle  Ankle plantar/dorsiflexion angle at initial contact | CNN model predictions deviated from the reference angles with a RMSE <3.5° and 6.5° in intra- and inter-participant scenarios, respectively. Intra- participant: R^2^ for hip, knee, and ankle angles was > 0.97, while the RMSE and NRMSE were < 3.4° and 4.6%, respectively. Mean difference between estimated and reference angles for all joints < 1°, MAE was up to 2.4°. Inter-participants: error of the estimated angles < 6.5°, with average R^2^ values of 0.84, 0.93, and 0.73 for hip, knee, and ankle, respectively. NRMSE <11% for hip, knee and ankle. |
| Kim et al. (2021)  [94] | To validate the ability of available IMUs to accurately measure the ankle joint angles by comparing the ankle joint angles measured using a wearable device with those obtained using a motion capture system during running | 10 (M)  **Age:** 30.2 ± 5.3  **Height:** 171.0 ± 15.3  **Weight**: 73.6 ± 12.4 | Recreational runners  **Exclusion:** History of ankle surgery or nervous system damage  Lower limb injury < 3 months prior | **IMU:** BNO55, Adafruit  **SF:** 100Hz  **Number:** 2  **Location:** Foot (Instep, right), Tibia | **Environment:** Indoor (Overground)  **Speed:** Controlled (2.68m/s)  **Distance/Time:** 5 trials | 3D Motion Capture (8 cameras, Vicon, 100Hz) | Ankle kinematics:  Sagittal  Frontal | Day-to-day reliability was excellent (0.974 and 0.900 for sagittal and frontal plane, respectively), and the validity was good in both sagittal (r = 0.821, p < 0.001) and frontal (r = 0.835, p < 0.001) planes for ankle joints |
| Koska et al. (2018)  [83] | To examine the validity of kinematic variables obtained from a single, shoe-mounted IMU using an opto-electronic motion analysis reference system. | 51 (36M, 15F)  **Age:** 33.9 ± 8.2  **Height:** 177.9 ± 7.6  **Weight**: 70.9 ± 10.1 | Healthy  Recreational runners | **IMU (3D Accelerometer, 3D Gyroscope)** Aims, Xybermind  **SF:** 400Hz  **Accelerometer Range:** 16g  **Gyroscope Range:** 2000°/s  **Size:** 6.0 x 1.85 x 0.5cm  **Weight:** 6g  **Number:** 1  **Location:** Shoe (Heel, right) | **Environment:** Indoor (Treadmill)  **Speed:** Controlled (10, 12, 15 km/hr)  **Distance/Time:** 3 x 3 mins  **Shoe:** Standardised (Nike Air Pegasus) | 3D Motion capture (14 cameras, Qualisys, 400Hz) | Sagittal ROM  Eversion ROM  Maximum eversion velocity  Rearfoot angles **Additional:**  Stride time  FSA  Heel strike  Heel off | Substantial systematic bias and large random disagreement are present in IMU data compared to the reference motion analysis, which increased at higher running speeds (characterized by substantial inter-individual differences in the error distribution). Discrepancies may partly be explained by differences in foot strike behaviour, resulting in varying degrees of vibration impact acting on the IMU. Relative disagreement was larger for frontal plane than sagittal plane variables. Shoe-mounted IMUs do not represent a valid method for obtaining kinematic foot variables. |
| Lee, Mellifont and Burkett (2010)  [75] | Determine the level of agreement between an inertial sensor and infrared camera-based estimates of stride, step, and stance durations across a range of running speeds | 10 (6M, 4F)  **Age:** 30.0 ± 8.0  **Height:** 174.3 ± 5.7  **Weight**: 67.7 ± 9.5 | Healthy  National standard runners | **IMU (3D Accelerometer):** KXM52 – 1050 Kionix, MiniTraqua Version 1 -Cooperative Research Centre for Microtechnology  **SF:** 100Hz  **Number:** 1  **Location:** Lower back (sacrum) | **Environment:** Indoor (Treadmill)  **Speed:** Controlled (10-12, 13-15, 16-19 km/hr)  **Gradient:** 1%  **Distance/Time:** 3 (days) x (3 (Speeds) x 5min)  **Shoe: -** | 3D Motion Capture (6 Infrared Cameras, Proreflex MCU 500 Hz, Qualisys Medical AB)  Force plates (Bertec Force Plate model 4080, Bertec Corporation) | Step time  Stride time  Stance time  Temporal gait events | Stride data showed agreement at < 0.02 s for most limits. Agreement for most of the data were < 0.020 s, coupled with small bias and systematic error, and very large to nearly perfect correlations (average r = 0.91) demonstrate strong agreement between both methods. The largest differences between both capture methods were for stance. A single inertial sensor positioned at the sacrum is suitable for identifying stride, step, and stance duration of running gait with suitable levels of agreement when compared to an infrared based system. Changes in a narrow range of running velocities did not influence the levels of agreement between the two measures. |
| Lee et al. (2010)  [76] | Validate a new technique (a sacral mounted single inertial sensor could identify the (COM) vertical acceleration) against a criterion measure and to determine the influence of changes in running intensity on COM vertical acceleration and the symmetry of COM vertical acceleration between left and right steps. | 10 (6M, 4F)  **Age:** 30.0 ± 8.0  **Height:** 174.3 ± 5.7  **Weight**: 67.7 ± 9.5 | Healthy  National standard runners | **IMU (3D Accelerometer):** KXM52 – 1050 Kionix, MiniTraqua Version 1 -Cooperative Research Centre for Microtechnology  **SF:** 100Hz  **Size:** 5.2 x 3.3 x 1.0cm  **Weight:** 21g  **Number:** 1  **Location:** Lower back (sacrum) | **Environment:** Indoor (Treadmill)  **Speed:** Controlled and self-selected (1 km/hr below self-selected pace, self-selected pace, 1 km/hr above**)**  **Gradient:** 1%  **Distance/Time:** 3 x 15s (8 complete strides, collected during final 1 min of each 5min run) | 3D Motion Capture (6 Infrared Cameras, Proreflex MCU 500 Hz, Qualisys Medical AB)  Force plates (Bertec Force Plate model 4080, Bertec Corporation) | Vertical COM | Near perfect correlations (r = 0.96) of COM vertical acceleration measured by both methods. Small typical error of estimate of 1.84 (95%CI;1.30–3.27) m/s2, and a low mean bias of 0.02 (±0.03). Running velocity increased the COM vertical acceleration tended to decrease. Greatest magnitude of COM vertical acceleration was at the slowest running pace and was significantly greater than that at the self-selected pace (p = 0.049) and fastest running pace (p = 0.017). A single inertial sensor can be used as a valid means of measuring COM vertical acceleration. |
| Machulik et al. (2020)  [102] | Evaluate the validity of the Humotion SmarTracks Integrated System (HSTI) for assessing spatio-temporal running gait parameters such as running speed, SL, and SF during jogging in a self-selected speed and fast sprinting on a 60 m tartan track. | 28 (18M, 10F)  **Age:** 28.2 ± 3.8  **Height:** 175.5 ± 9.5 **Weight:** 70.6 ± 10.7 | Healthy  Free of injury (> 6 months) and disease | **IMU (3D Accelerometer, 3D Gyroscope, 3D Magnetometer)**  **SF:** 400Hz  **Accelerometer Range**: 16g  **Gyroscope Range:** 2000°/s  **Size:** 13.0 x 3.2 x 0.9cm  **Weight:** 31g  **Number:** 1  **Location:** Lower back (L3-L5) | **Environment:** Outdoor (Track)  **Speed:** Self-selected (jogging & sprinting)  **Distance/Time:** 6 x 60m (2x 0-20m, 2x 20-40m, 2 x 40-60m) | Photoelectric cell system (Opto Jump Next, Microgate, 1000Hz). | SF  SL  Speed | High measurement agreement for running speed, SL and SF for both velocities (ICCs 0.745-0.996). High random errors and increased systematic and random errors for SL and SF at fast running velocities. Systematic increase of bias (systematic error) with higher SL values. |
| McGrath et al. (2012)  [27] | Compare the performance of a body-worn wireless gyroscope-based gait analysis application to a marker-based motion capture system for the detection of heel-strike and toe-off and subsequent calculation of gait parameters during walking and running | 5 (4M, 1F)  **Age:** 26-32 | Healthy | **IMU (3D Gyroscope):** Shimmer, Shimmer Inc  **SF:** 102.4 Hz  **Number:** 2  **Location:** Tibia | **Environment:** Indoor (Treadmill)  **Speed:** Controlled (8, 12 km/hr (Additional walking 2, 4 km/hr))  **Distance/Time:** 2 (trials) x 2 (speeds) x 20s (Additional 4 walking trials)  **Shoe:** Own | 3D Motion Analysis (CODA, 200Hz) | Stride time  Swing time  Stance time  Heel-strike  Toe-off | Adaptive gyroscope algorithm can calculate stride time with similarly high accuracy. Stride time indicate that there was <1 ms of true error and <1.6 percentage error between each comparison across all speeds. Poor to moderate agreement for stance and swing times. At 12 km/hr, comparisons that included the Adaptive gyroscope algorithm resulted in higher levels of agreement (ICCs: 0.83, 0.69) than the reference algorithms (0.60) for stride time, while at 8 km/hr all comparisons yielded moderate levels of agreement. |
| Nuesch et al. (2017)  [103] | To compare joint kinematics measured by the inertial sensor system RehaGait® with those of an optoelectronic system (Vicon®) for treadmill walking and running. Additionally, the test re-test repeatability of kinematic waveforms and discrete parameters for the RehaGait® was investigated | 20 (8M, 12F)  **Age:** 27.4 ± 8.3  **Height:** 175.0 ± 8.0  **Weight:** 66.5 ± 12.5 | Healthy  **Exclusion:** Lower leg injuries < 6 months prior | **IMU (3D Accelerometer, 3D Gyroscope, and 3D Magnetometer)**: RehaGait® system (Version 2.0.7.2), Hasomed GmbH  **SF:** 400Hz  **Accelerometer Range:** 16g  **Gyroscope Range:** 2000°/s  **Number:** 7  **Location:** Lower back (L4), Thigh (lateral middle), Tibia (lateral lower third), Shoe (lateral heel) (Bilaterally) | **Environment:** Indoor (Treadmill)  **Speed:** Self-selected  **Distance/Time:** 2 x 20 strides (Additionally done for walking)  **Shoe:** Own | 3D Motion Capture (6 camera, Vicon MX, 200Hz)  Instrumented treadmill with embedded plantar pressure plate (h/p/cosmos, Zebris FDM-T; 7168 sensors, 120Hz) | Sagittal plane kinematics of the ankle, knee and hip joints | Joint angles measured by the 2 systems were highly correlated, but only after offset correction. Coefficient of multiple correlation between the knee kinematics measured with the RehaGait system and the reference system was very good, while moderate for the ankle kinematics and weak for the hip kinematics. RMSE of the calculated ranges of motion in the three joints ranged from 4° to 9°, while the RMSE of the other discrete parameters ranged from 13° to 36°. Repeatability: coefficient of multiple correlation was very good for the ankle (0.937) and excellent for the knee and hip joint (>0.984). The RMSE of the waveforms measured by the two systems/models was between 3° and 7° for running. |
| Patoz et al. (2021)  [95] | To propose a method that used data recorded by a single sacral-mounted IMU to estimate vGRF, GCT and FT | 100 (74M, 26F)  **Age:** M: 30.0 ± 8.0  F: 30.0 ± 7.0  **Height:** M: 180.0 ± 6.0  F: 169.0 ± 5.0  **Weight:** M: 71.0 ± 7.0  F: 61.0 ± 6.0 | Healthy  Recreational runners  Free of lower-extremity injury >1 month | **IMU**: Movesense  **SF:** 208Hz  **Accelerometer Range:** 8g  **Weight:** 9.4g  **Number:** 1  **Location:** Lower back (sacrum) | **Environment:** Indoor (Treadmill)  **Speed:** Controlled (9, 11, 13km/hr)  **Distance/Time:** 3 (speeds) x 10 strides | 3D Motion Capture (Vicon, 200Hz)  Instrumented Treadmill (Arsalis T150–FMT-MED) | GCT  FT  vGRF | Comparison between GSM and IMU method depicted RMSE ≤22 ms (≤14%) for effective GCT and effective FT along with small systematic biases (≤20 ms) for each speed. |
| Patoz et al. (2022)  [96] | To propose a method that uses the vertical acceleration recorded using a sacral-mounted IMU to estimate effective GCT and effective FT and to compare these estimations to those from gold standard methods | 100 (73M, 27F)  **Age:** M: 30.0 ± 8.0  F: 29.0 ± 7.0  **Height:** M: 180.0 ± 6.0  F: 169.0 ± 5.0  **Weight:** M: 71.0 ± 7.0  F: 61.0 ± 6.0 | Healthy  Recreational runners  Free of lower-extremity injury >1 month | **IMU**: Movesense, Suunto  **SF:** 208Hz  **Accelerometer Range:** 8g  **Weight:** 9.4g  **Number:** 1  **Location:** Lower back (sacrum) | **Environment:** Indoor (Treadmill)  **Speed:** Controlled (9, 11, 13km/hr)  **Distance/Time:** 3 (speeds) x 10 strides | 3D Motion Capture (Vicon, 200Hz)  Instrumented Treadmill (Arsalis T150–FMT-MED) | GCT (effective)  FT (effective)  vGRF | RMSE of 0.15 BW (6%) was obtained for peak vGRF, max while a RMSE of 20ms was reported for both GCT and FT (8% and 18%, respectively). Small systematic biases of 0.07 BW for peak vGRF and 13ms for GCT and FT, yet RMSEs were smaller than the smallest real differences [peak vGRF: 0.28 BW (11%), GCT: 32ms (13%), and FT: 32ms (30%)], indicating no clinically important difference between 3D motion capture and IMU. |
| Seutain et al. (2018)  [104] | Examine the validity of MIMUs compared to force plate platform recordings when evaluating force – velocity relationship of the sprint mechanics and to determine whether the data provided by a MIMU placed at the lumbar spine could reliably assess this sprint mechanics related variables | 16 (8M, 8F)  **Age:**  M: 31.5 ± 6.3  F: 26.1 ± 4.4  **Height:**  M: 177.0 ± 7.0  F: 166.3 ± 7.4  **Weight:**  M: 78.3 ± 13.0  F: 59.8 ± 8.0 | Healthy  Recreational runners  Minimum training frequency of 2 x/week and met at least one of the following criteria: 10 km < 55 min, or 13.1 mile <1hr 50min | **IMU (3D Accelerometer, Gyroscope, Magnetometer)**: MTx, 3DOF Human Orientation Tracker, Xsens Technologies  **SF:** 120Hz  **Number**: 1  **Location:** Lower back (L4-L5) | **Environment:** Indoor (Overground)  **Speed:** Self-selected (max. sprint)  **Distance/Time:**  4 x 20m | Force plates (Custom build force transducers, Raute Precision 1000Hz)  Photocell gates (3, Newtest) | GRF  Velocity  Force  Decrease in ratio of forces  Applied horizontal force loss  Maximal power | Extremely large to very large correlation levels between MIMU sensor-based sprint mechanics variables and force plate recordings (velocity force plate: 8.61 ± 0.85, MIMU 8.4 ± 0.69. Force 383 ± 110, 391 ± 103. Power 873 ± 246, 799 ± 241. Horizontal force loss -44.6 ± 12.7, 46.2 ± 10.7), ranging from 0.88 to 0.94. Decrease in ratio of forces showed weak to moderate correlations (r = 0.45). Step-averaged force values measured with both systems were highly correlated (r=0.88). Very large to extremely large retest correlation coefficients for inter-trial reliability of MIMU measurements (r = 0.72 - 0.96). |
| Shiang et al. (2017)  [84] | To determine the FSP at different speeds by using IMUs and investigate the similarity of the signals between IMU and motion analysis system to examine the validity of sensor | 6 (M)  **Age:** 25.4 ± 1.7  **Height:** 175.0 ± 5.2 **Weight:** 72.4 ± 5.7 | Healthy  Free of injury (>6months) and cardiovascular diseases | **IMU (3D Accelerometer, 3D Gyroscope)**: CXL25GP3 – Crossbow, SEN-09412/09425- SparkFun  **SF:** 200Hz  **Accelerometer Range:** 25g  **Number:** 2  **Location:** Shoe (dorsal) | **Environment:** Indoor (Treadmill)  **Speed:** Controlled (7, 10 and 13 km/hr)  **Distance/Time:** 2 (FSPs) x 3 (speeds) x 15s (60s trial) | 3D Motion Capture (10 camera system, Vicon MX 13+, 200Hz). Treadmill (MAG-7310, Magtonic, Taiwan) with 4 load cells (Delta transducer, India), analog data collected by an acquisition system (MP150, 1000Hz) | Stride index  **Additional:**  Sagittal plane kinematics  Pitch angle | Results showed that both the Vicon system and IMUs exhibited highly correlated changes between different FSPs in the sagittal plane (𝑟 = 0.98). The analysis of strike index and sensitivity in identifying landing strategies showed that the sensitivity reached its optimal value when the strike index was 0.115. |
| Struber et al. (2021)  [106] | To validate S-Move reliability through comparison of its repeatability with a commonly used optoelectronic device for angular kinematic stride and an instrumented treadmill for spatiotemporal parameters | 30 (19M, 11F)  **Age:** 26.6 ± 7.8  **Height:** 174.0 ± 9.0  **Weight:** 68.4 ± 14.0 | Recreational  **Exclusion:** Pain and/or lower leg injuries within 6 months prior | **IMU (3D Accelerometer, 3D Gyroscope, 3D magnetometer)**: S-Move, TIMC-IMAG  **Accelerometer Range:** 4g  **Gyroscope Range:** 1000°/s  **Number:** 5  **Location:** Sacrum, thigh, tibia | **Environment:** Indoor (Treadmill)  **Speed:** Self-selected  **Distance/Time:** 3 (sessions) x 2 minutes, (Additional walking: 3 (sessions) x 2 minutes)  **Shoe:** Own | Optoelectronic system (Codamotion)  Instrumented Treadmill (Zebris FDM-TDS-3i, Zebris Medical GmbH) | SF  SL  Stride length  Step duration  Stride duration  Speed  Hip adduction  Hip flexion  Hip rotation  Pelvic tilt  Pelvic obliquity  Knee varus  Knee flexion  Knee rotation | S-move system- absolute and relative RMSE was 6.4° (min: 2.1°; max: 10.9°) and 2.8° (min: 1.0°; max: 4.5°) respectively. Reproducibility was 2% for temporal parameters and 5-6% for spatial parameters. |
| van Werkhoven, Farina and Langley (2019)  [26] | To validate the use of the BioStampRC in determining FSP against traditional video analysis techniques using a high-speed video camera. To determine whether these sensors could accurately detect changes FSA, and differences in FSA during running, and whether the sensors could detect different FSPs | 12 (M/F)  **Age**: 18 - 45  **Height:** 176.0 ± 12.0 **Weight:** 75.2 ± 23.2 | Healthy  Free of injury (>3 months) and disease | **IMU (3D Accelerometer, 3D Gyroscope):** BioStampRC - mc10  **SF:** 250Hz  **Accelerometer Range:** 16g  **Gyroscope Range:** 2000°/s  **Size:** 6.6 x 3.4 x 0.45cm  **Weight:** 7g  **Number:** 1  **Location:** Foot (dorsal side, right) | **Environment:** Indoor (Treadmill)  **Speed:** Self-selected  **Distance/Time**: 3 (FSPs) x 10 right foot strikes (60s trial) | High speed camera (STC-MBS241U3V, Sentech Technologies, 120 frames per second, resolution of 800 x 600) | FSA  FSP (foot angular velocity, resultant acceleration, % stride) | Sensor was able to accurately (92.2% success) distinguish between RFS and NRFS (compared to 2D video analysis) using an angular velocity cut-off value of 0°/s. Strong and significant correlation between FSA determined by the sensor and from videography analysis (r = 0.868, p < 0.001), although the sensor underestimated the FSA (bias/offset of 11.2°). |
| Winter et al. (2016)  [78] | To validate acceleration data from a single inertial sensor containing a tri-axial accelerometer, whilst running overground during a prolonged run against a motion analysis system. Determine whether specific events in the running gait cycle (heel strike and toe off) were identified consistently by both data collection methods | 10 (6M, 4F)  **Age:** 27.5 ± 9.5  **Height:** 175.8 ± 8.1  **Weight:** 69.5 ± 11.8 | Healthy  Recreational runners  Ran > 30 km/week  Free of injury  No lower extremity abnormalities that affected their gait | **IMU (3D Accelerometer)**  **SF:** 100Hz  **Accelerometer Range:** 8g  **Size:** 5.2 x 3.3 x 1.0cm  **Weight:** 21g  **Number:** 1  **Location:** Lower back | **Environment:** Indoor (Overground)  **Speed:** Self-selected  **Distance/Time:** 5 x 20m | 3D Motion Capture (12 cameras, NEXUS v1.8, Vicon Motion Systems Ltd, 100Hz) | Heel-strike  Toe-off  Acceleration | 3D acceleration data collected from a single inertial sensor in over ground running during a prolonged run is highly correlated with simultaneous data collected using a motion analysis system. Specific events of heel strike and toe- off in the gait cycle are clearly identifiable from the inertial sensor in the anteroposterior and craniocaudal acceleration data. |
| Wouda et al. (2018)  [97] | To examine the validity of a method to estimate sagittal knee joint angles and vertical ground reaction forces during running using an ambulatory minimal body-worn sensor setup | 8 (M)  **Age:** 25.1 ± 5.2  **Height**: 183.7 ± 4.5  **Weight**: 77.7 ± 9.4 | Healthy  Experienced runners  Free of injury | **IMU**  **SF:** 240Hz  **Number:** 3  **Location:** Waist, Tibia (bilaterally) | **Environment:** Indoor (Treadmill)  **Speed:** Controlled (10, 12, 14 km/hr)  **Distance/Time:** 3 (speeds) x 3 min | 3D Motion Capture (6 cameras, MX-13, Vicon, 100Hz)  Instrumented Treadmill (S-Mill, ForceLink, 1000Hz)  IMUs (Xsens MVN Link inertial motion capture system, 240Hz, 17 devices (shoulders, upper arms, lower arms, hands, upper legs, lower legs, feet, head, sternum, and pelvis (bilaterally))) | Knee flexion/extension  PTA  vGRF (Peak vGRF and loading rate) | Estimated kinematics and kinetics of most subjects show excellent agreement (p > 0.99) with the reference, for single subject training. Knee flexion/extension angles are estimated with a mean RMSE <5◦. GRFs are estimated with a mean RMSE < 0.27 BW. Peak vGRF, loading rate and maximal knee flexion during stance were compared, however, no significant differences were found. With multiple subject training the accuracy of estimating discrete and continuous outcomes decreases, however, good agreement (p > 0.9) is still achieved for 7 of the 8 different subjects. |
| Wundersitz et al. (2015)  [38] | To compare peak acceleration data from an accelerometer contained within a wearable tracking device with a criterion measure of acceleration, derived from a motion analysis system, while walking, jogging and running. Also investigated the effect different filtering cut-off frequencies have on accelerometer accuracy, agreement, precision and relative error | 39 (28M, 11F)  **Age:** 24.2 ± 2.5  **Height:** 179.0 ± 9.0  **Weight:** 71.6 ± 12 |  | **IMU (3D Accelerometer)** MinimaxX s4, Catapult Innovations  **SF:** 100Hz  **Size:** 8.8 x 5.0 x 1.9cm  **Weight:** 67g  **Number:** 1  **Location:** Upper back (harness) | **Environment:** Indoor (Treadmill)  **Speed:** Controlled (3.3 (jogging), 5.0 (female), 5.9 (male) m/s)  **Distance/Time:** 2 (speeds) x 10 footstrikes. (Additional 10 footstrikes walking) | 3D Motion Capture (12 camera, Raptor-E, Motion Analysis Corporation, 200Hz)  Instrumented treadmill (Quinton Q65, Quinton Inc.) | Peak resultant acceleration | Peak raw accelerometer acceleration significantly overestimated peak motion analysis acceleration. The lower the cut-off frequency, the smaller the relative error found, with the higher frequency cut-offs typically resulting in significant overestimations (20 Hz and 15 Hz) and the lower frequency cut-off (6 Hz) resulting in significant underestimation of motion analysis accelerations. 10 Hz cut-off frequency displayed the best accuracy with the motion analysis system. As the magnitude of acceleration recorded increased, the validity of the accelerometer decreased. |
| Zrenner et al. (2020)  [85] | Evaluate whether the sensor position of IMUs mounted to running shoes has an impact on the accuracy of different spatiotemporal parameters | 29 (23M, 6F)  **Age:** 24.9 ± 2.4 | Healthy  Recreational runners | **IMU (3D Accelerometer, 3D Gyroscope):** miPod  **SF:** 200Hz  **Accelerometer Range:** 16g  **Gyroscope Range:** 2000°/s  **Number:** 8  **Location:** Shoe (cavity of the shoe, lateral ankle, heel, instep (bilaterally)) | **Environment:** Indoor (Overground)  **Speed:** Controlled (2-6 m/s)  **Distance/Time:** 50 trials: (20 (trials) x 2 (speeds)) + (30 (trials) x 2 (speeds)) (Overall: 2426 strides)  **Shoe:** Standardised (Adidas Response Cushion 21) | 3D Motion Capture (16 camera, Vicon Motion Systems, 200Hz) | GCT  Stride length  Stride time  Stride velocity  Sole angle  Eversion | IMUs measure different raw data depending on their position on the foot and that the accuracy of the spatiotemporal parameters depends on the sensor position (especially for the accelerometer signals). Recommend integrating IMU sensors in a cavity in the sole of a running shoe under the foot’s arch, because the raw data of this sensor position is best suitable for the reconstruction of the foot trajectory during a stride. |
| *Accelerometers* |  |  |  |  |  |  |  |  |
| Alexander et al. (2016)  [129] | Determine the validity of an accelerometer to measure average acceleration values during high-speed running. | 13 (M)  **Age:** 23.9 ± 2.2  **Height:** 186.6 ± 8.4  **Weight:** 102.5 ± 12.2 | Healthy.  Professional rugby union players in the Super Rugby competition. | **3D Accelerometer:**  SPI-HPU, GPSports  **SF:** 100Hz  **Size:** 7.4 x 4.2 x 1.6cm  **Weight:** 16g  **Number:** 1  **Location:** Upper back | **Environment:** Indoor (Track)  **Speed**: Self-selected (max. sprint)  **Distance/Time**: 3 x 20m (40m trial, 0-10m and 10-20m analysed) | Dual-beam timing gates (Swift)  Multiple stationary machine vision cameras (Allied Vision Technologies) | Average acceleration Average velocity | Accelerometer could not measure average acceleration values during high-speed running, significantly overestimated average acceleration values during both 0–10 m and 10–20 m, regardless of the data filtering technique (p < 0.001). Body mass significantly affected all accelerometer variables (p < 0.10, partial n2 = 0.091–0.219). |
| Edwards et al. (2019)  [130] | To determine whether trunk-mounted accelerometers were a valid and reliable method to estimate thoracic segment or centre of gravity (COG) acceleration or vGRF, and the whether the elasticised harness contributes to the overestimation of acceleration. | 10 (M)  **Age:** 21.0 ± 2.0  **Height:** 181.0 ± 5.0  **Weight:** 81.8 ± 11.1 | Healthy  Male  Rugby union players (University’s rugby team) | **3D Accelerometer:** SPI-HPU, GPSports  **SF:** 1000Hz  **Accelerometer Range:** 16g  **Size:** 7.4 x 4.2 x 1.6cm  **Weight:** 66g  **Number:** 1 or 2  **Location:** Upper back (T1 to T6 vertebrae (in pouch of harness)) | **Environment:** Indoor (Overground)  **Speed:** Controlled; (3.3, 5.0, 6.7 m/s)  **Distance/Time:** 3 x 10-foot strikes (5 foot strikes each foot, at 3 different speeds, approx. 10 run-up strides) | 2 wireless Trigno electromyography devices with inbuilt 3D accelerometers with their electromyography function disabled (Delsys, 148Hz, 14G).  3D motion capture (10 Oqus 300+ camera system 300Hz)  Force plates (2 multichannel Kistler, Type 9281CA and 9821EA, 1200Hz) | vGRF  Peak vertical acceleration  Time from IC to peak vertical acceleration | Accelerometers demonstrated poor reliability (ICC:0.0–0.67), high variability (CV%:14–33%) and change in mean (41–160%),and were not valid to estimate vertical acceleration of the COG and thoracic segment nor vGRF. |
| Encarnación-Martínez et al. (2021)  [34] | Investigate the validity and reliability of a new device integrated directly into the treadmill, compared to a traditional acceleration impact system | 30 (20M, 10F)  **Age:** M: 27.2 ± 7.5, F: 24.4 ± 6.1  **Height:** M: 175.6 ± 5.1, F: 161.3 ± 4.3  **Weight:** M: 73.3 ± 8.0, F: 55.8 ± 4.0 | Healthy  RFS  Physically active (to run a minimum of 2x/week in the last year, do 2 h and 30 min a week of moderate-intensity, or 1 h and 15 min a week of vigorous-intensity aerobic- physical activity)  Free of injury (> 6 months) and disease  Not be taking medication that hinders stability during the running  **Exclusion:** Significant illness, injury, or surgery within 6 months  Overweight or obese | **3D Accelerometer**: AccAthl, Blautic  **SF:** 415Hz  **Size:** 4.0 x 2.2 x 1.2cm  **Number:** 2  **Location:** Tibia (distal anteromedial) | **Environment:** Indoor (Treadmill)  **Speed:** Controlled (10km/hr)  **Gradient:** 0%  **Distance/Time:** 2 (sessions) x 10s (of last minute of 5)  **Shoe:** Own | Accelerometery system implemented in the treadmill | SF  SL  Step time  PTA  Peak impact asymmetry | AccAthl demonstrated excellent reliability for measuring spatiotemporal parameters like step length (ICC = 0.88), step time (ICC = 0.89), and step frequency (ICC = 0.91). Peak acceleration impact variables for the left (ICC = 0.88) and right leg (ICC = 0.90), and peak impact asymmetry (ICC = 0.75). |
| Gindre et al. (2016)  [110] | To assess the reliability and validity of the Myotest® accelerometer-based system for measuring running stride kinematics. | 20 (M)  **Age:** 31.6 ± 9.2  **Height:** 178.0 ± 5.4  **Weight:** 72.5 ± 9.8 | Healthy  Habitual Runners | **Accelerometer** Myotest -Myotest SA  **SF:** 500Hz  **Accelerometer Range:** 16g  **Number:** 1  **Location:** Waist (level with the navel, fixed onto a neoprene belt) | **Environment:** Indoor (Overground (Flat cement))  **Speed:** Controlled (12, 15, 18 and 21 km/hr)  **Distance/Time:** 8 x 60m | High Speed Video Analysis (2 x Casio High Speed EXILIM EX-FH25®, CASIO Europe GmbH, 300Hz)  Light based optical technology (Optojump Next, Microgate, 1000Hz)  Timing gate system (Racetime2, MicroGate, Timing and Sport) | GCT  SF  FT | Myotest®-derived parameters were highly reproducible between trials at all speeds (ICC: 0.886 to 0.974). Compared to the reference measures, the mean GCT from the Myotest® were 34% shorter and FTs were 64% longer. Only SF was comparable between systems and demonstrated high between-system correlation (ICC ≥ 0.857). |
| Gouttebarge et al. (2015)  [120] | To assess the reproducibility (test-retest reliability and agreement) and concurrent validity of the Myotest for measuring SF and GCT in recreational runners. | 14 (11M, 3F)  **Age:** 45.0 ± 14.0 **Height:** 181.0 ± 7.0  **Weight:** 77.0 ± 11.0 | Healthy  Recreational runners  Free of injury (>1 month)  Ran weekly for > 1 month  > 18 years | **3D Accelerometer**: Myotest Run - Myotest SA  **SF:** 200-500Hz  **Size:** 5.42 x 10.25 x 1.7cm  **Weight:** 59g  **Number:** 1  **Location:** Waist (Velcro waistband around the iliac crest, ventral side) | **Environment:** Outdoor (Track)  **Speed:** Controlled (10, 12, 14 km/hr)  **Distance/Time:**  2 (different days) x (3 x 400m)  **Shoe:** Own | Foot-mounted 3D accelerometer (+6 g; 1000 Hz, MMA7361L, Freescale Semiconductor, attached at each shoe of the participant by using the shoelace) | GCT  SF | SF, test-retest reliability (ICCs > 0.75) and agreement of the Myotest were considered as good at all running speeds. GCT, test-retest reliability was moderate at a speed of 14 km/h and poor at speeds of 10 and 12 km/h (ICC < 0.50). Concurrent validity of the Myotest with the foot-mounted accelerometer at all 3 running speeds was found to be good for SF (ICCs > 0.75) and moderate for GCT (0.50 < ICCs < 0.75). |
| Kenneally-Dabrowski, Serpell and Spratford (2018)  [133] | To validate a method that used accelerometery data to determine running symmetry during maximal over ground sprinting for abled bodied athletes | 13 (M)  **Age:** 23.8 ± 2.4  **Height:** 186.6 ± 8.4  **Weight:** 102.5 ± 12.2 | Healthy  Professional rugby union players  Free of injury | **3D Accelerometer**: GPSports  **SF:** 100Hz  **Accelerometer Range:** 16g  **Number:** 1  **Location:** Upper back (between scapulae, against the spine, housed in vest) | **Environment:** Indoor (Track)  **Speed:** Self-selected (max. sprint)  **Distance/Time:** 3 x 40m | Multiple stationary machine vision cameras (Allied Vision Technologies, 1000Hz)  Force plates (8 x (600m x 900mm), Kistler, 1000Hz, located between 25 and 32.2m) | Symmetry index **Additional:**  Stride time  GRF | Trivial relationship (r=0.088, p=0.616) and poor agreement (bias=0.189, p=0.000) between accelerometer and forceplates for calculating running symmetry. Stride time exhibited a small relationship (rs=-0.177, p=0.310) and significant bias (bias=-0.026, p=0.000), yet showed smaller LoA. Both methods for measuring sprinting asymmetry using accelerometer data had poor internal validity. However, stride time showed the best capacity to calculate running symmetry during maximal effort sprints. |
| Navalta et al. (2019)  [134] | To provide reliability data for outdoor tasks as measured by the Stryd Power Meter, which is a footpod monitor. | 20 (12M, 8F)  **Age**: 22.2 ± 5.8  **Height:** 174.6 ± 9.9  **Weight:** 71.2 ± 14.4 | Healthy  Free of disease | **3D Accelerometer**: Stryd Power Meter, Boulder  **Weight:** 10g  **Number:** 1  **Location:** Shoelaces (right) | **Environment:** Outdoor (Trail)  **Speed:** Self-selected  **Distance/Time:** 2 x 5 min (Additional 2 x 5 min walks)  **Shoe:** Own | - | GCT  Vertical oscillation  Average elapsed power  Maximal power  Average elapsed form power  Average elapsed leg spring | Measures during trail running that returned a CV less than 10 %, met the ICC threshold of 0.70, and displayed good to excellent 95 % CI included pace, average elapsed power, average elapsed form power, average elapsed leg spring, and vertical oscillation. |
| Nedergaard et al. (2018)  [121] | Explored the feasibility of using measured trunk accelerometery to estimate the MSD-model parameters required to predict resultant GRF during running. | 20 (M)  **Age:** 22.0 ± 4.0  **Height:** 178.0 ± 8.0 **Weight:** 76.0 ± 11.0 | Healthy  Male  Participated in a running related sport >1x/week | **3D Accelerometer**: KXP94 - Kionex (embedded within a GPS device (MinimaxX S4, Catapult Innovations))  **SF:** 100Hz  **Accelerometer Range:** 13g  **Size:** 8.8 x 5.0 x 1.9cm  **Weight:** 67g  **Number:** 1  **Location**: Upper back (Dorsal part of the upper trunk between the scapulae within a small pocket of a tight fitted elastic vest) | **Environment:** Indoor (Overground)  **Speed:** Controlled (2, 3, 4, 5 m/s)  **Distance/Time:** 4 (footstrikes) x 4 (Speeds) | Force plate (9287C, Kistler Instruments Ltd., 3000Hz, 0.9 x 0.6 m2) | Resultant trunk acceleration **Additional:**  GRF | Despite a good match between the measured trunk accelerometery and the mass spring damper-model's upper mass acceleration (median RMSE between 0.16 and 0.22 g), poor GRF predictions (median RMSE between 6.68 and 12.77 N/kg) were observed. Mass spring damper-model was able to replicate the measured GRF with high accuracy (median RMSE between 0.45 and 0.59 N/kg) across running speeds from GRF parameter. The model parameters from measured trunk accelerometery under- or overestimated the GRF parameter obtained from measured GRF, and generally demonstrated larger within parameter variations. |
| Nijs, Beek and Roerdink (2021)  [135] | To assess test–retest reliability, face validity and concurrent validity for cadence and stance time in running using instrumented earbuds equipped with accelerometers | 14 (6M, 8F)  **Age:** 36.0 ± 13.0  **Weight:** 71.4 ± 14.1 | Healthy  Recreational runners | **3D Accelerometer**: LISDW12, STMicroelectronics  **SF:** 800Hz  **Number:** 1  **Location**: Ear (left) | **Environment:** Indoor (Treadmill)  **Speed:** Controlled & self-selected (7 km/h, 10 km/h, 13 km/h, and the max. speed (< 16 km/h))  **Distance/Time:** 2 (intra-session) x 4 (speeds) x 60s (Additional 2-minute trial with head-movements) | Instrumented Treadmill (Motek, 500Hz) | SF  Stance time | Within-Method Reliability: Speed Condition: ICC showed good-to-excellent agreement for the earbud (ICC > 0.817). Face Validity: Difference over Speed Conditions: Both methods, cadence increased significantly with speed (earbud: F(1.04, 8.35) = 11.16, p = 0.009; force plate: F(1.04, 8.34) = 10.97, p = 0.01), with significant post-hoc differences between 13 km/h and the other two speed conditions (p < 0.017). Between-methods agreement for cadence was excellent for all speeds and instructed head movements. For stance time, agreement was good-to-excellent for all conditions, except 13 km/h and shaking the head. |
| Raper et al. (2018)  [136] | To determine the validity of a commercially available tibial mounted accelerometer as a calculation of GRF compared to the reference standard force plates, and determine its absolute and relative reliability for use in running at a constant velocity | 10 (4M, 6F)  **Age:**  M: 27.0 ± 5.5.  F: 26.8 ± 3.1  **Height:**  M: 177.5 ± 4.7.  F: 164.5 ± 2.9  **Weight:**  M: 66.0 ± 5.3.  F: 54.8 ± 3.2 | Healthy  Elite/Sub-elite (Triathlon or a sport where running predominates)  **Exclusion:** Lower limb soft tissue injuries < 3 months prior  Lower limb surgery < 12 months | **3D Accelerometer** ViPerform v5, DorsaVi  **SF:** x=100, z =20Hz  **Number:** 1  **Location:** Tibia (medial border) | **Environment:** Indoor (Track)  **Speed:** Self-selected (slow, medium, fast)  **Distance/Time:** 10 x 50m (15m acceleration, 20m distances for constant speed and 15m deceleration) | High-speed video (10 Prosilicia high-speed cameras, Allied Vision Technologies, 200Hz)  Force plate (8, Kistler Instrument Group, 1000Hz) | Vertical peak GRF **Additional:**  GRF  Braking force  Impulse | Accuracy of the ViPerform was calculated to be 83.96%. Reliability of the ViPerform v5 was excellent (ICC =0.877; 95% CI = 0.825 – 0.917) in calculating the same vGRF in a repeated trial. ViPerform does not calculate absolute values of vGRF similar to those measured by a force plate. Provides a valid and reliable calculation of an athlete’s lower limb load at constant velocity. |
| Rowlands, Stone and Eston (2007)  [117] | To determine the relationships of triaxial accelerometry, uniaxial accelerometry, and pedometry with speed and SF across a range of walking and running speeds. | 9 (M)  **Age:** 23.1 ± 3.4  **Height**: 177.9 ± 5.6  **Weight:** 72.5 ± 8.1 | Healthy  Trained short- and middle-distance runners | **1D Accelerometer:** Actigraph- GT1M  **3D Accelerometer:** RT3 –StayHealthy  **SF:** 1D - 30Hz  **Accelerometer Range:**  1D - 2.13g  3D - 0.05-2.0g  **Number:** 1D- 2. 3D- 2  **Location:** Hips | **Environment:** Indoor (Treadmill)  **Speed:** Controlled (8, 10, 12, 14, 16, 18, 20, 22, 24, and 26 km/hr)  **Distance/Time:** 2 sessions (test-retest): 20 steps at 10 speeds. (6 (speeds) x 60s and 4 (speeds) x 30s). (Additional walking 3 (speeds) x 60s) | SF was calculated as the mean of the two visual counts | SF | Increasing underestimation of activity by the ActiGraph as speed increases is related to frequency-dependent filtering and assessment of acceleration in the vertical plane only. RT3 vector magnitude was strongly related to speed, reflecting the predominance of horizontal acceleration at higher speeds. High-intensity activity is underestimated by the ActiGraph, even after correction for frequency-dependent filtering, but not by the RT3. Pedometer output is highly correlated with SF |
| Sheerin et al. (2017)  [138] | To determine between-session reliability and variability of resultant tibial acceleration at four running speeds (at one-week and six-month intervals). To investigate differences in the natural variability in tibial acceleration over a longer period of time (6-months), the smallest worthwhile change in tibial acceleration and how this related to measurement error | 14 (M)  **Age:** 33.6 ± 11.6  **Height:** 177.0 ± 5.0 **Weight:** 75.6 ± 9.5  **Retest:**  8 (M)  **Age:** 37.4 ± 5.8  **Height:** 175.0 ± 5.0  **Weight:** 72.3 ± 6.0 | Healthy  Free of injury | **3D Accelerometer**:  IMeasureU  **SF:** 1000Hz  **Accelerometer Range:** 16g  **Weight:** 12g  **Number:** 2  **Location:** Tibia (distal anteromedial) | **Environment:** Indoor (Treadmill)  **Speed:** Controlled (2.7, 3.0, 3.3, 3.7 m/s)]  **Distance/Time:** 4 x 50s (2 min trials)  **Shoe:** Standardised (Asics Kudrow) for tests. Own running shoes for training. | Instrumented treadmill (Bertec) | PTA | While runners demonstrated marginally lower reliability and higher variability over six months, compared to one week, in all cases the measures of reliability and variability were of ‘good’ to ‘moderate’ reliability, and ‘small’ to ‘moderate’ variability using magnitude-based inferences. ICC ranged from 0.90 to 0.96 for the baseline to one-week comparison, and 0.89–0.95 for the baseline to six-month comparison, which indicated excellent agreement and low measurement variability between testing sessions. |
| Sinclair et al. (2013)  [63] | To determine the efficacy of a method for the determination of gait events using an accelerometer mounted to the distal tibia, measuring axial accelerations. | 16 (11M, 5F)  **Age:** 29.4 ± 5.7  **Height:** 173.0 ± 4.9  **Weight:** 67.8 ± 10.7 | Healthy  RFS | **Accelerometer:** ACL300, Biometrics  **SF:** 1000Hz  **Weight:** 9g  **Number**: 1  **Location:** Tibia (distal anteriomedial aspect, longitudinal axis 0.08 m above the medial malleolus) | **Environment:** Indoor (Overground)  **Speed:** Controlled (4 m/s)  **Distance/Time:** 10 footstrikes | Force plate (Model 9281CA, Kistler, 1000Hz)  Timing gates (SmartSpeed Ltd). | Heel-strike  Toe-off  Stance time | Absolute and the average error between the heel strike, toe-off and stance times calculated using the accelerometer signal method compared with the force platform were all < 12 ms. Strong correlations (r = .96) observed between duration of stance obtained using the 2 different methods. The error values compare favourably to other alternative methods of predicting gait events. Shank-mounted accelerometers can be used to detect gait events accurately and reliably. |
| Sinclair and Sant (2017)  [115] | To determine whether specific gait events could be accurately and consistently identified using an accelerometer mounted to the distal tibia, measuring axial accelerations, by contrasting the predicted events to those detected using force data. | 13 (M)  **Age:** 27.8 ± 7.0  **Height:** 177.0 ± 11.0  **Weight:** 76.2 ± 7.0 | Healthy  Free of injury | **Accelerometer:**  ACL300, Biometrics  **SF:** 1000Hz  **Number:** 1  **Location:** Tibia (distal anteriomedial aspect, longitudinal axis 0.08 m above the medial malleolus) | **Environment:** Indoor (Overground)  **Speed:** Controlled (4 m/s)  **Distance/Time:** 3 (footwear types) x 5 (trials) x right footstrikes  **Shoe:** Standardised (conventional (New Balance 1260 v2), minimalist (Vibram five-fingers, ELX) and cross-fit (Reebok Cross-Fit CR) | 3D Motion Capture (8 camera, Qualisys Track Manager, 250Hz, Visual 3D, C-Motion)  Force plate (Kistler, 1000Hz).  Timing gates (Newtest). | PTA  Tibial acceleration slope  **Additional:**  Stance Time  FSA  Lower extremity kinematics  IVLR | Instantaneous loading rate and peak tibial acceleration were significantly larger in the barefoot (274.19 BW/s and 5.72 g) and minimalist (302.91 BW/s and 5.31 g) conditions in relation to the conventional (112.37 BW/s and 4.55 g) footwear. Ankle angle at footstrike was significantly more plantarflexed in the barefoot (-8.70°) and minimalist (-.92°) conditions compared to the conventional (5.09°) and cross-fit (2.16°) conditions. |
| *Pressure Insoles* |  |  |  |  |  |  |  |  |
| Burns, Zendler and Zernicke (2019)  [140] | Assessed the validity and reliability of a new wireless in-shoe system (Novel Loadsol/Pedoped) for field-based ground reaction force measurement in hopping, walking, and running. | **Validity:** 20 (11M, 9F)  **Reliability:** 10 (5M, 5F)  **Age:**  **Validity:** 23.0 ± 3.2.  **Reliability:** 23.0 ± 2.5  **Weight:**  **Validity:** 68.2 ± 11.4  **Reliability:** 64.2 ± 12.4 | Healthy  Self-reported runners  Engaging in ≥ 30 minutes of running ≥ 3 times per week Free of lower limb injury or use of orthotics. | **Pressure Insole:** Novel Loadsol insole, Novel Gmbh  **SF**: 100Hz  **Number:** 2  **Location**: Insole | **Environment:** Indoor (Treadmill)  **Speed:** Controlled (10km/hr)  **Distance/Time:** 1 or 2 (days) x 200 consecutive left and right steps of a 5 min run (>1 day apart)  **Shoes:** Own | Instrumented treadmill (h/p/cosmos Quasar, 100Hz) | GCT  Peak vGRF  Total impulse | Insoles demonstrated good-to excellent agreement with the treadmill across all measures (ICCs: 0.88–0.96) and were reliable across sessions (ICCs within 0.00–0.03). |
| Chumanov, Remy and Thelen (2010)  [141] | Evaluated the feasibility of using insole pressure sensors together with whole body dynamics to analyse joint kinetics while running | 8 (4M, 4F)  **Age:** 25.3 ± 3.5  **Height:** 173.5 ± 5.5  **Weight:** 68.8 ± 6.9 | - | **Pressure Insole:** Novel Inc  **SF:** 100Hz  **Number:** 2  **Location**: Insole | **Environment:** Indoor (Overground)  **Speed:** Self-selected; Preferred and fast (Additional walking trials; slow, preferred, fast)  **Distance/Time:** 3 (trials) x 2 (speeds). (Additional 3 (trials) x 3 (speeds) walking trials)  **Shoes:** Own | 3D motion capture (Motion Analysis, 8 cameras, 200Hz)  Force plates (AMTI, 2000Hz) | vGRF  CoP | Insole tracking algorithm generated estimates for the vGRF and COP trajectories that were within one SD of forceplate measures. RMS differences in vGRF were < 10% of peak vertical force (80–130 N) for running. Medio-lateral COP RMS errors during mid-stance (between 10 and 80% of stance phase) were <8 mm during walking and running. Anterio-posterior COP errors were <12 mm between 10 and 80% of stance phase. The largest error in the estimated COP occurred during heel contact and prior to toe-off. |
| Cramer et al. (2022)  [142] | Evaluate whether the Insole3 is a valid and reliable alternative to force plates for measuring vGRF | 11 (8M, 3F)  **Age:** 33.1 ± 16.7  **Height:** 174.0 ± 8.9  **Weight:** 74.2 ± 14.6 | Healthy  Recreational runners  Pain-free, functional in daily activities without the need of assistance, and absent of history of surgery on the ankles, knees, and hips | **Pressure Insole:** Insole3, OpenGo, Moticon ReGo  **SF:** 100Hz  **Number:** 2  **Location**: Insole | **Environment:** Indoor (Overground)  **Speed:** Controlled (3.3 - 3.7m/s)  (Additional: Walking: 0.8 - 1.4 m/s)  **Distance/Time:** 2 (sessions) x 5 footstrikes on each foot.  (Additional 2 (sessions) x 2 (speeds) x 5 footstrikes walking)  **Shoes:** Standardised (Adidas low-cup VRX, model DB3176) | 2D Video Analysis  3D Motion Capture (12 camera, Qualysis, 300Hz)  2 x Force Plates (Bertec, 3000Hz) | Peak vGRF  Impulse | Excellent agreement during running for the single vGRF peak (ICC = 0.942) and impulse (ICC = 0.940). Peak force during running was split between over- and underestimations and had a relatively small mean bias (% bias = 0.9%). Reliability was excellent (ICC > 0.970) |
| Mann et al. (2014)  [144] | To devise a valid definition of FSP by comparing the strike index using the Runalyser to the actual FSA. Secondly, to validate temporal parameters by comparing Runalyser data to that acquired using an instrumented treadmill measuring GRFs. The third aim was to test the reliability of the output of the Runalyser using a test–retest approach. | 31 (24M, 7F)  **Age:** 30.3 ± 7.4  **Height:** 178.0 ± 10.0  **Weight:** 74.1 ± 12.1 | Healthy  Free of injury  Accustomed to running on a treadmill | **Pressure Insole:** Runalyser, TNO  **Size:** 0.3cm  **Number**: 2  **Location**: Insole | **Environment:** Indoor (Treadmill)  **Speed:** Self-selected  **Gradient**: 0%  **Distance/Time:**  Strike-index validation: 3 (FSP) x 60s.  Temporal parameter validation: 2 min  Reliability: 2 (days) x 2 min + (3 (FSP) x 60s)  **Shoe:** Standardised | Motion analysis (3 CX1 cameras, CODAmotion, 1000Hz)  Instrumented treadmill (Mercury LT med, HP Cosmos, 1000Hz) | Stride time  Strike-index  FT  Stance time  Initial contact  Toe-off  Duty factor  **Additional:**  FSA  GRF | High agreement (overall mean difference 1.2%) was found between stance time, flight time, stride time and duty factor as determined by the Runalyser and a force-measuring treadmill. Measurements of the two devices were highly correlated (R > 0.80) and not significantly different. Test–retest ICCs for all parameters were > 0.94. Significant differences (p < 0.05) between FFS, RFS and habitual running were detected regarding SI, stance time and stride time. Strike index and FSA had a strong relationship (R2 = 0.89). Runalyser was able to discriminate different running styles. |
| Renner, Williams and Queen (2019)  [149] | Aims to determine if the loadsol®—a single sensor wireless insole—is a valid and reliable method of assessing force. To determine the between-day reliability as well as the validity of the loadsol® compared to an instrumented treadmill during walking and running at various speeds and inclines | 100Hz: 30 (17M, 13F). 200Hz: 10 (6M, 4F)  **Age:**  M: 20.9 ± 2.4,  F: 21.4 ± 3.0  **Height:**  M: 179.0 ± 7.0.  F: 169.0 ± 5.3  **Weight:**  M: 73.5 ± 13.7.  F: 64.6 ± 7.9 | Healthy  Recreationally active -  exercising at least 3x/week for > 30 min  Aged 18-30 years  Treadmill experience | **Pressure Insole:** Loadsol, Novel GmbH  **SF:** 100, 200Hz  **Number:** 2  **Location:** Insole | **Environment:** Indoor (Treadmill)  **Speed:** Controlled (3.0 and 3.5 m/s (Walking 1.3 m/s))  **Gradient:** 0%, 10% Incline, 10% Decline  **Distance/Time:**  100Hz: 2 sessions x (2 (speeds) x 3 (gradients) x 30s (60s trials)). Additional 3 walking trials.  200Hz: 2 (speeds) x 3 (gradients) x 30s (60s trials). Additional 3 walking trials.  **Shoe:** Standardised (Nike Air Pegasus) | Force plates within a fore–aft split belt, instrumented treadmill (2 force plates, Compact Tandem Force-Sensing Treadmill, Model: DBCEEWI, AMT, 1440Hz) | Peak weight acceptance force  Impulse  Loading rate  **Additional:**  vGRF | ICCs ranged from 0.61 to 0.97 indicating a good to excellent association between the loadsol® and force plates. The 200 Hz validity results indicated excellent validity for peak load, impulse, and loading rates across all of the conditions. For between-day reliability, the loadsol ICC values range from 0.90 to 0.99 for peak weight acceptance, 0.86–0.98 for impulse, and 0.72–0.99 for loading rate. Peak force and impulse were all classified as having excellent reliability while loading rate ranges from good to excellent. There is a significant bias in the peak load between the force plate and loadsol®. The loadsol is underestimating the force plate for the peak force and impulse measurements. |
|  |  |  |  |  |  |  |  |  |
| Seeley et al. (2020)  [123] | To test the accuracy of a new sensing technology, different from IMUs, in predicting important characteristics of vGRF during running at three different speeds. | 31 (17M, 14F)  **Age:** 23.0 ± 3.0  **Height:** 174.4 ± 8.0 **Weight:** 68.0 ± 10.0 | Healthy  Recreational runners  Shoe sizes: M: US 9.5, 10.5, or 11.5, F: 7, 8, or 9 for female  Aged 18-30 years  Free of injury >6 months  No history of lower-extremity surgery | **Pressure Insole**  **3D Accelerometer:** Bosch Sensortec  **SF:** Pressure sensor – 1029Hz  Accelerometer – 16Hz  **Accelerometer Range:** 16g  **Number:** Pressure sensor – 8  Accelerometer – 1  **Location:** Insole (toe, ball, arch, heel (bilaterally)) Accelerometer: Shoe | **Environment:** Indoor (Treadmill)  **Speed:** Controlled (2.68, 3.13, 3.58 m/s)  **Distance/Time:** 3 (speeds) x 4 min  **Shoe:** Standardised | High-speed video (Vicon 250Hz)  Instrumented force treadmill (AMTI, 100Hz) | GCT  Impact peak vGRF  Active peak vGRF  Impulse  Average impact peak load rate  Maximum instantaneous impact  Peak load rate | Percentage error of the resulting predictions varied depending upon the predicted vGRF characteristic. Percentage error was relatively low for predicted vGRF impulse (2–7%), active peak vGRF (3–7%), and GCT (3–6%), but relatively high for predicted vGRF load rates (22–29%). |
| Seiberl et al. (2018)  [150] | To quantify the systematic and random error of this new wearable and wireless sensor technology (loadsol® force measurement insoles) over an extended period of use as compared to a gold standard device in a laboratory setting. | 10 (6M, 4F)  **Age:** 21.8 ± 0.8  **Height:** 174.9 ± 9.4  **Weight:** 69.4 ± 13.7 | Healthy  No neural or orthopaedic disorders  Can run pain-free | **Pressure Insole:** Loadsol, Novel GmbH  **SF:** 100Hz  **Number:** 2  **Location:** Insole | **Environment:** Indoor (Overground)  **Speed:** Self-selected  **Distance/Time:** 80 steps (4 (sets) x 2 (FSPs) x 5 (trials (5 left and 5 right steps))  **Shoes:** No shoes (Wore the insoles under tight-fitted socks without shoes) | Force plates (AMTI, 1000Hz) | GCT  Initial contact  Toe-off  Active peak force  Time to active peak  Positive and negative force rate | Significant differences (p < .05) in GCT, peak force, and force rate, while there was no difference in parameters impulse, time to peak, and negative force rate. Mean bias of GCT, impulse, peak force, and time to peak ranged between 0.6% and 3.4%, demonstrating high accuracy of loadsol® devices for these parameters. For these same parameters, the LoA analysis showed that 95% of all measurement differences between insole and force plate measurements were less than 12%, demonstrating high precision of the sensors. Highly dynamic behaviour of GRF, is not yet sufficiently resolved by the insole devices. |
| Stöggl and Martiner (2017)  [124] | Experimental validation of the OpenGo sensor insole system compared to PedarX sensor insole and AMTI force-plate systems | 16 (14M, 2F)  **Age:** 31.0 ± 10.0  **Height:** 180.0 ± 8.0 **Weight:** 77.0 ± 11.0 | Healthy  Sport science students | **Pressure Insole:** OpenGo, Moticon GmbH. PedarX Mobile System, Novel GmbH (Reference)  **SF:** OpenGo - 50Hz  Pedar 50Hz  **Accelerometer Range:**  OpenGo - 8g  **Number**: 4  **Location:** Insole | **Environment:** Indoor (Overground)  **Speed:** Self-selected  **Distance/Time:** 4 x 15m (Additional 8 x 15m walking)  **Shoe:** Standardised (Adidas Supernova) | Force plate (AMTI BP600900, 1000Hz) | GCT  FT  Swing time  GRF  Impulse | By combining pressure and acceleration, the OpenGo GCT was in high accordance with the reference systems, particularly AMTI. Larger discrepancies were observed when comparing running data of OpenGo and PedarX. Force impulse with OpenGo was generally lower, the difference increased with increasing speed and was particularly low for running. Time to the first force peak with OpenGo was significantly longer for running. OpenGo sensor insoles, accurate detection of cycle characteristics during gait can be provided. |
| Zhang, Guo, Zanotto (2020)  [98] | Support vector regression models can be used to extract accurate estimates of fundamental gait parameters (i.e., stride length, velocity, and foot clearance), from custom-engineered instrumented insoles (SportSole) during walking and running tasks | 14 (M)  **Age**: 23.1 ± 4.0  **Height**: 178.0 ± 5.0  **Weight:** 75.4 ± 11.5 | Healthy  Free of injury and disease | **Pressure Insole (containing IMU)**: SportSole Insole: IEE Inc.,  **IMU:** Yost Labs Inc.  **SF:** 500Hz  **Size:** 0.4cm  **Number**: 2  **Location:** Insole (IMU placed along the midline of the foot) | **Environment:** Indoor (Treadmill)  **Speed:** Controlled (85%, 115%, preferred)  **Distance/Time:** 2 (sessions) x 2 (speeds) x 3 (minutes) (Additional 2 x 2 x 3 minutes walking)  **Shoe:** Own | 3D Motion Capture (8 camera, Vicon, Vero v2.2, 300Hz)  Force Plates (Bertec ITC-11-20L, 900Hz) | Stride length  Gait events  Stride velocity  Foot clearance | SVR models yielded excellent ICC in all the gait parameters analyzed. MAE% in stride length, velocity, and foot clearance obtained with SVR models were 2.59% ±0 .64%, 2.91%± 0.85%, and 5.13% ±1.52% for running, respectively. Both subject specific and SVR models could improve accuracy while also showing better interrater and test-retest reliability than linear models. |
| *Gyroscopes* |  |  |  |  |  |  |  |  |
| Fadillioglu et al. (2020)  [127] | To investigate whether a gait event detection algorithm for various locomotion tasks would provide comparable estimation accuracies as existing, task-specific algorithms. | 13 (M)  **Age:** 26.1 ± 2.9  **Height:** 178.7 ± 5.5  **Weight:** 78.4 ± 5.9 | Healthy  Free of injury | **Uni-axial Gyroscope:** ADXRS652, Analog Device Inc  **SF:** 1500Hz  **Gyroscope Range:** 250°/s  **Number:** 1  **Location:** Tibia | **Environment:** Indoor (Overground)  **Speed:** Controlled and self-selected; (Fast running was set at 150 % of moderate speed)  **Distance/Time:** 3 (trials) x 2 (speeds) (Additional: walking straight, 90° walking turns to the left and right and 45° and 90° running cuts to the left and right) | Force plates (2, BP600900 AMTI, 1000Hz)  Light barriers (TAG Heuer) | Initial contact  Toe-off  Mid-swing  **Additional:**  GRF | Moderate running: Absolute mean error IC 10 ± 4 ms; TO 26 ± 20 ms. Relative absolute mean error IC 3.35 ± 1.38 %; TO 8.0 ± 4.8 %. Fast running: Absolute mean error IC 13 ± 6 ms; TO 23 ± 23 ms. Relative absolute mean error IC 5.5 ± 2.7 %; TO 9.43 ± 8.76 %. The proposed algorithm is capable of detecting gait events by means of a single gyroscope located on the shank. |
| *Combination of validity/reliability and/or application* | | |  |  |  |  |  |  |
| *Inertial Measurement Units* | |  |  |  |  |  |  |  |
| Kozinc, Smajla, Šarabon (2022)  [87] | To investigate the reliability of running biomechanics assessment with a wearable commercial sensor and to assess the differences between surface and running speed | 24 (14M, 10F)  **Age:** 28.1 ± 4.7  **Height:** 174.6 ± 7.4 **Weight:** 66.7 ± 8.4 | Healthy  Free of injury (>6 months),  neurological and non-communicable chronic diseases  Ran > 10km/week | **IMU (3D Accelerometer, 3D Gyroscope):** RunScribe, Scribe Labs Inc.  **SF:** 200Hz  **Accelerometer Range:** 16g  **Gyroscope Range:** 2000°/s  **Number:** 2  **Location:** Shoe (shoelaces) | **Environment:** Outdoor (Asphalt, grass, concrete)  **Speed:** Controlled (Estimated max. 5km or 13.1 mile pace)  **Distance/Time:** 2 (sessions) x 3 (surfaces) x 3 (trials) x 200m  **Shoe:** Own |  | GCT  SF  Stride length  FSP  FT  Pronation excursion  Peak pronation velocity  Peak vGRF,  Vertical GRF rate, Horizontal GRF rate  Impact g  Braking g  Power  Shock | Intra-session relative reliability was excellent for all variables when running at 5-km pace (ICC asphalt: 0.90–0.99; macadam: 0.94–1.00; grass: 0.92–1.00). For 21-km pace on the asphalt, most variables also showed excellent relative reliability (ICC > 0.88). Relative inter-session reliability was high or excellent for the majority of the outcomes (ICC ≤ 0.85). Stride length decreased during running on asphalt at 21-km pace compared to 5-km pace (p < 0.001; d = 0.43–0.45). |
| Mitschke, Kiesewetter, Milani (2018)  [61] | To systematically investigate the influence of operating range on the accuracy of stride length, running velocity, and on PTA | 21 (M)  **Age:** 24.4 ± 4.2  **Height:** 178.2 ± 4.0 **Weight:** 74.1 ± 6.5 | Healthy  RFS  Free of injury for >6 months  UK size 8 | **IMU (2D Accelerometer, 2D Gyroscope):** ADXL278 – Analog Devices, IDG-650 - InvenSense  **1D Accelerometer:** ADXL78- Analog Devices.  **SF:** 1000Hz  **Accelerometer Range:** 70g  **Gyroscope Range:** 2000°/s  **Number:** 2  **Location:** IMU: Shoe (heel, right), 1D accelerometer: Tibia | **Environment:** Indoor (Track)  **Speed:** Self-selected  **Distance/Time:** 15 (3 footwear conditions, 5 trials) x 15m  **Shoe:** Standardised (Adidas AdiStar, Puma Speed 600, Asics Gel Nimbus 12) | Timing gates | Stride length  Velocity  PTA | Operating range influenced the outcomes of all investigated parameters, which were not influenced by tested footwear conditions. The lower operating ranges were associated with an underestimation error for all biomechanical parameters, which increased noticeably with a decreasing operating range. Accelerometers with a minimum operating range of ± 32 g should be used to avoid inaccurate measurements |
| Napier et al. (2021)  [62] | To assess the validity of two commercially available IMUs during running. Secondary aims were to determine the effect of footwear, running speed, and IMU location on PPA | 20 (11M, 9F)  **Age:** 35.9 ± 8.3 | Healthy  RFS  Free of musculoskeletal and neurological pain  Running > 3 months  Shoe size: M: 8–12 US,  F: 6–10 US | **IMU:** RunScribe, Scribe Labs Inc.  Plantiga, Plantiga Technologies  IMeasureU, Blue Thunder  **SF:** RunScribe & Plantiga: 500Hz  IMeasureU: 100Hz  **Number:** 4  **Location:** IMeasureU: Tibia,RunScirbe & IMeasureU: Shoe (shoelaces), Plantiga: Insole | **Environment:** Indoor (Treadmill)  **Speed:** Controlled & self-selected (Preferred speed, +10%, -10% of preferred speed)  **Distance/Time**: 3 (shoe types) + 2 (additional speeds) x 30 steps (right foot)  **Shoe:** Standardised shoes (Neutral: New Balance 880v9, Minimalist: Merrell Trail Glove 5, Maximalist: New Balance Fresh Foam More v1). | Instrumented Treadmill (Bertect Corporation, 2000Hz) | PPA  **Additional:**  AVLR | AVLR had a high positive association with PPA (IMeasureU-Tibia) in the neutral and maximalist (r = 0.70–0.72; p < 0.001) shoes and in all running speed  conditions (r = 0.71–0.83; p < 0.001), but low positive association in the minimalist (r = 0.47; p < 0.05) footwear condition. The relationship between AVLR and  PPA (Plantiga) was high in the minimalist (r = 0.75; p < 0.001) condition and moderate in the neutral (r = 0.50; p < 0.05) and maximalist (r = 0.57; p < 0.01) footwear. RunScribe metrics demonstrated low to moderate positive associations (r = 0.40–0.62; p < 0.05) with AVLR across most footwear and speed conditions. The commercially available Plantiga IMU is comparable to a tibia-mounted IMU when acting as a surrogate for AVLR but results vary between different levels of footwear and running speeds |
| *Accelerometers* |  |  |  |  |  |  |  |  |
| Giandolini et al. (2014)  [116] | To validate a simple method using continuous measurements to identify the running pattern in situ. | Validation: 14 (11M, 3F)  Application: 34 (19M, 15F)  **Age:**  Validation: 24.6 ± 10.3. Application: 44.1 ± 8.9  **Height:**  Validation: 177.0 ± 6.0. Application: 173.0 ± 8.0  **Weight:**  Validation: 68.1 ± 8.9. Application: 67.7 ± 9.5 | Healthy  Experienced (Ultra-trail runners) | **1D Accelerometer**: ADXL150, Analog Device  **SF:** 1000Hz  **Accelerometer Range:** 5g  **Number:** 2  **Location:** Shoe (right, heel & 5th metatarsal | **Environment:** Indoor (Treadmill)  **Speed:** Controlled and self-selected (10, 12, 14 (F), 16 (M) km/hr, max. aerobic speed, preferred speed)  **Distance/Time:** Validation: 10 x 15s/10 consecutive steps (2min trials). Application: 5 x 15s/10 consecutive strides (2min trials)  **Shoe:** Validation: Standardised (Salomon XRMissionCS), Application: Own | 2D Video Analysis (Basler scA640-120gc, Basler AG, 120Hz; Simi Motion 2D Software, Simi Reality Motion Systems GmbH) | Time between heel and metatarsal peak accelerations  **Additional:**  FSP  FSA | Significant correlations were observed between time between heel and metatarsal peak accelerations and foot-treadmill contact angle for 14 out of 15 conditions. Overall correlation coefficient was r=0.916 (P<0.0001, n=288). Time between heel and metatarsal peak accelerations method is highly reliable for a wide range of speeds and slopes, and for all types of foot strike except for extreme forefoot strike during which the heel rarely or never strikes the ground, and for different footwear and states of fatigue. |
| *Pressure Insoles* |  |  |  |  |  |  |  |  |
| Akhlaghi et al. (1994)  [25] | Identify trends in normal step-to-step PP patterns obtained using the Gaitscan system | 17 (8M, 9F)  **Age:** 19 - 40 | Healthy  No obvious foot deformity | **Pressure Insole**: GaitScan  **SF:** 250Hz  **Size:** 1.0 x 1.0 x 0.28cm  **Number**: 2  **Location:** Insole | **Environment:** Indoor (Overground)  **Speed:** Self-selected  **Distance/Time:** 2 (sets) x 5 (runs) x 14 steps (7 left, 7 right)  **Shoe:** Own (Footwear was required to conform to the following criteria: 1) Low heels, not greater than 3 cm. 2) Adjustable laces to secure the shoe on the foot. 3) A round toe-box. 4) Adequate depth and width in which to fit the insole. 5) Everyday comfortable footwear, with no excessive wear of the soles) |  | PP | Variations of 73% in PP at the first and fifth metatarsal heads, for one subject of 10 runs and a 66% variation within each run. Similar variations up to 100% were seen with the other subjects. |
| Henning and Milani (1995)  [45] | Assess the influence of several commercially available running shoes on foot pressures in a large group of subjects during over ground running. To examine the suitability of using eight discrete pressure sensors, positioned under selected anatomical structures of the foot, to estimate the total load experienced by the foot during running. | 22  **Age:** 29.4 ± 6.8  **Weight:** 71.7 ± 6.0 | Healthy  Experienced Runners Normal foot structure  No history of injury | **Pressure Insole**: Halm PD-16, Halm GmBH  **SF:** 1000Hz  **Size:** 0.4 x 0.4 x 0.2cm  **Number**: 8  **Location:** Insole (Medial and lateral heel and midfoot; the first, third, and fifth metatarsal heads; and the hallux) | **Environment:** Indoor (Overground)  **Speed:** Controlled (3.3m/s)  **Distance/Time:** 5 (trials) x 19 (shoes) x 1 (footstrike)  **Shoe:** Standardised | Force plates (Kistler (Type 928 1 B, 1000Hz))  Entran accelerometer (Type EGAX-F-25, Tibia, 1.5g, 1000Hz) | PP  Relative impulses  Relative loads  **Additional:**  GRF  Peak acceleration | Early lateral loading of the rearfoot was followed by increasing medial forefoot loads. In the later phase of pushoff the load was almost entirely carried by the first metatarsal head and the hallux. Substantial differences in plantar foot pressures and relative loads among shoe models indicated that footwear construction has a substantial influence on the loading behaviour of the foot during ground contact. Chosen sensor locations under the foot were found to be adequate to estimate the vertical ground reaction force |
| Kernozek and Zimmer (2000)  [47] | To assess the reliability of Pedar system during slow treadmill running at two different speeds and examine how in-shoe loading parameters change with increasing running speed | 17  **Age:** 24.6 ± 4.7 | Healthy  Free from any gait abnormalities or lower extremity pathologies that could affect running ability | **Pressure Insole**: Pedar, Novel GmbH  **SF:** 150Hz  **Number:** 2  **Location:** Insole | **Environment:** Indoor (Treadmill)  **Speed:** Controlled (2.24 and 3.13 m/s)  **Distance/Time:** 2 (days) x 2 (speeds) x 8 consecutive footfalls (20s)  **Shoe:** Own |  | PP  Contact area  Maximum force  Force-time impulse  PP-time impulse | Reliability ranged from 0.84-0.99 for plantar loading measurements at both running speeds examined between the days tested. The lateral forefoot region was the least reliable region of the plantar surface analysed while the heel, mid forefoot and central forefoot were more reliable. Running speed did not seem to alter the reliability of the plantar loading parameters at the speeds tested. All plantar loading variables increased (PP, PP time impulse, peak force, and force time impulse) with the exception of contact area when treadmill running speed was increased. Control of running speed is essential in obtaining reproducible data. |
| *Application* |  |  |  |  |  |  |  |  |
| *Inertial Measurement Units* |  |  |  |  |  |  |  |  |
| Ahamed et al. (2019)  [28] | Determine the number of runs needed to establish a stable running pattern during level, downhill, and uphill conditions for both univariate and multivariate analyses of running biomechanical data collected using a single wearable IMU device | 35 (10M, 25F)  **Age:**  M 54.8 ± 9.6  F 47.6 ± 11.1  **Height:**  M 174.9 ± 5.8  F 47.6 ± 11.1  **Weight:**  M 80.5 ± 7.8  F 64.7 ± 8.2 | Healthy  Recreational runners  Registered for a marathon training program managed by local running group  Free of injury and neuromuscular diseases | **IMU (3D Accelerometer, Magnetometer, Gyroscope)**: Lumo Run, Lumo BodyTech.  **SF:** 100Hz  **Number:** 1  **Location**: Lower back (posterior aspect. Attached to the back of the shorts or to a running belt near the individual’s COM) | **Environment:** Outdoor  **Speed:** Self-selected  **Gradient:** Mixed condition: (the entire run and irrespective of elevation); level: (+2%); uphill: (+3% to +15%); downhill: (-3% to -15%)  **Distance/Time**: 7 (runs) x 9000 m (from km 1 to 10). Averaged data for each 10-strides  **Shoe:** Own | GPS (Garmin Vivoactive HR, Garmin, measuring velocity and SF) | GCT  SF  Vertical Oscillation  Pelvic Rotation  Pelvic Drop  Braking  **Additional:**  Speed  Distance | 2–4 runs needed to define a stable running pattern for univariate, and 4–5 days were necessary for multivariate analysis to reach stability irrespective of elevation. Pearson’s correlation coefficients showed excellent correlations (r = 0.98 to 1.0) comparing the training and testing data within the same elevation condition and good to very good correlations (r = 0.63–0.88) when comparing training and testing data from differing elevation conditions. |
| Bailey, Mata and Mercer (2017)  [91] | Compare the relationship between stride length, stride frequency, and velocity while running on a treadmill and over ground. | 10 (8M, 2F)  **Age:** 22.3 ± 2.6  **Height:** 171.0 ± 8.0  **Weight**: 71.4 ± 15.5 | Healthy  Physically active  Free of injury | **IMU:** GarminFenixGPX2, Garmin  **SF:** 10Hz  **Number:** 1  **Location:** Shoe | **Environment:** Indoor (Treadmill), Outdoor (Concrete)  **Speed:** Controlled (Various speeds)  **Distance/Time**: 7 x 100m (outdoor), 7 x 60s (treadmill) | GPS (GarminFenixGPX2, Garmin, measuring velocity and SF) | Stride frequency  Stride length  Velocity | SL – SF combination selected was dependent on whether the participant was running on a treadmill or over ground. SL-velocity relationship had more curvature indicative of a plateau while achieving faster velocities over ground, but not so on a treadmill. Participants behaved differently when attempting to achieve faster running velocities over ground versus running on a treadmill. |
| Brahms et al. (2020)  [42] | Investigate the effects of a fatiguing distance running protocol on different measures of stride pattern variability and long-range correlations in a group of elite and recreational runners. | 32 (16 Elite, 16 Recreational)  **Age:**  Elite: 21.2 ± 3.0, Recreational: 26.8 ± 4.8  **Height:**  Elite: 175.6 ± 8.7, Recreational: 174.5 ± 7.4  **Weight:**  Elite: 63.4 ± 9.5, Recreational: 71.6 ± 10.5 | Healthy  Elite: trained at > 4 days/week during the previous 2 years, competed at a provincial or intercollegiate level Recreational: young, healthy adults who participated in recreational running for < 3 hr/week, no specific training related to competitive running. Capable of running at a vigorous speed for > 15 min.  **Exclusion:** Lower-extremity surgery, RRIs that caused discontinuation of running for > 7 days during the year preceding the experiment  Neuromuscular or cardiovascular pathology, or any medical condition | **IMU (3D Accelerometer, 3D Gyroscope, 3D Magnetometer):**  Xsens MTw  **SF:** 100Hz  **Gyroscope Range:** 2000°/s  **Number:** 1  **Location:** Shoelaces (right) | **Environment:** Indoor (Track)  **Speed:** Controlled (Personal 5 km race pace)  **Distance/Time:** Timed distance run around a 200 m indoor track at the prescribed speed until the 5k pace could no longer be maintained |  | GCT  Stride length  Stride time  Peak impact acceleration **Additional:**  Speed  Distance  Duration | Significant group main effects were found for mean SL, GCT and PIA, as well as ST CV. Run duration exhibited a significant main effect for mean PIA, as well as the FSI for SL, ST, GCT and PIA. Runners maintained a consistent SLT, ST and GCT during the run, while PIA increased. Across groups, long-range correlations significantly decreased over the course of the run, while the magnitude of the variability remained constant. LRCs did not differ significantly between groups. |
| Bräuer et al. (2021)  [43] | To investigate ‘ride’ and rollover with a heel cap-mounted IMU when running under field conditions to get realistic results | 20  **Age:** 29.0 ± 7.3  **Height:** 176.4 ± 4.3  **Weight**: 69.4 ± 6.5 | RFS  Free of injury  Shoe size UK 8 | **IMU (3D Accelerometer, 3D Gyroscope):** ICM-20601, InvenSense.  **SF:** 2000Hz  **Accelerometer Range**: ± 353 m/s^2^  **Gyroscope Range**: 4000 °/s  **Weight:** 4g  **Number:** 1  **Location:** Shoe (right, heel) | **Environment:** Outdoor (Asphalt)  **Speed:** Controlled (3.0 m/s)  **Distance/Time:** 5 (shoe types) x 1km  **Shoe:** Standardised (Puma) |  | PAV | The shoe with a medium midsole bending stiffness had the lowest value for PAV (845.6 °/s) and the best rating of perceived ‘ride’ on average. IMU can be used as a low-cost method to investigate the heel–toe transition during field-running. |
| Clermont et al. (2019)  [29] | To use wearable technology data to quantify alterations in subject-specific running patterns throughout a marathon race and to determine if runners could be clustered into subgroups based on similar trends in running gait alterations throughout the marathon. | 27 (12M, 15F)  **Age:**  M: 50.4 ± 13.0  F: 40.9 ± 10.3  **Height:**  M: 174.9 ± 10.3  F: 160.5 ± 4.3  **Weight:**  M: 79.0 ± 12.0  F: 58.2 ± 7.8 | Healthy  ≥ 18 years  Official registration for a marathon race  **Exclusion:** Cardiac risk  Lower-extremity RRI < 6-months  Lower-extremity surgery  Use of orthotics, or medical conditions/ medications that would impair balance | **IMU (3D Accelerometer, 3D Gyroscope, 3D Magnetometer):** Lumo Run, Lumo Bodytech Inc.  **SF:** 100Hz  **Number:** 1  **Location:** Lower back (near COM Back of runner's shorts, aligned with spine) | **Environment:** Outdoor (Concrete)  **Speed:** Self-selected (Marathon pace)  **Distance/Time:** Marathon segments: 4-14km, 2km test sections throughout (i.e. 14-16, 16-18, …40-finish) | GPS (Garmin Vivoactive HR, Garmin, measuring velocity and SF) | GCT  SF  Vertical oscillation  Pelvic rotation  Pelvic drop  Braking | A significant effect of race section on biomechanical index, F(4.28,111.16) = 13.78, P < .001. Speed was not a significant confounding factor for the changes in biomechanical indices. Recreational runners with higher age-grade performance scores had less atypical running patterns throughout the marathon compared with runners with lower age-grade performance scores. Wearable technology can be used to investigate the effects of fatigue on running biomechanics. |
| Clermont, Pohl and Ferber (2020)  [72] | To use COM acceleration patterns to investigate changes in running patterns prior to, two and seven days following a marathon race | 17 (7M, 10F)  **Age:**  M: 47.7 ± 15.3  F: 34.2 ± 5.7  **Height:**  M: 177.2 ± 12.7  F: 160.8 ± 4.52  **Weight:**  M: 79.9 ± 15.4  F: 59.3 ± 8.1 | Healthy  ≥ 18 years old  Official registration for the 2017 or 2018 Scotiabank Calgary Marathon races  **Exclusion**: Cardiac risk  Lower-extremity RRI < 6-months  Lower-extremity surgery  Use of orthotics, or medical conditions/ medications that would impair balance. | **IMU (3D Accelerometer)**: Shimmer3, Shimmer Inc  **SF:** 201.03Hz  **Accelerometer Range**: 8g  **Number:** 1  **Location:** Lower back (L3 - L5 vertebrae, near COM, elastic straps) | **Environment:** Indoor (Track)  **Speed:** Self-selected  **Distance/Time:** 3 x 5 mins (7mins in total, 1st and last min excluded) |  | SF  ST  Stride time  REGSTEP_ML  REGSTEP_V  REGSTEP_AP  REGSTEP_RES  REGSTRIDE_ML  REGSTRIDE_V  REGSTRIDE_AP  REGSTRIDE_RES  PEAKML  PEAKV  PEAKAP  PEAKRES  RMSML  RMSV  RMSAP  RMSRES  RATIOML  RATIOV  RATIOAP | No evidence to suggest that time point had any effect on SF, ST or stride time. Peak medial-lateral COM increased at 2 days post compared with pre, but little difference at 7 days post. Observed changes in COM motion at 2 days post marathon race may be associated with atypical running biomechanics that can translate to greater mediolateral impulses, potentially increasing the risk of injury. |
| DeJong and Hertel (2020)  [80] | Describe five individual competitive adult runners’ biomechanical outcomes during unique in-field running demands that were captured using heel-mounted RunScribe™ wearable sensors. | 5 (1M, 4F)  **Age:** 30.2 ± 3.3  **Height:** 167.4 ± 8.7  **Weight**: 64.9 ± 11.8 | Healthy  Aged 26-45 years  Distance running at least 5x/week amounting to > 24km  Competed at the regional to national level at distance ranging from 800m to 42km  Involved in running training at least 3x/week over the past 2 years  **Exclusion:** Any current RRI  History of lower extremity injury  Surgery within the past 6 months  Known pregnancy | **IMU (3D Accelerometer, 3D Gyroscope):** RunScribe Plus, RunScribe Labs  **SF:** 200Hz  **Number**: 2  **Location:** Shoe (heel) | **Environment:** Outdoor (Concrete, Track, Trail)  **Speed:** Self-selected  **Gradient:** varying gradients  **Distance/Time:**  Track intervals 2 x 1600m, 800m, 400m, 200m.  Hill sprints: 5 x 150m uphill, downhill recovery.  Stroller running: 2 x 6.44km, (1 x with stroller, 1 x without).  1x 21.1km race: flat.  2 x 5km races: hilly course (598-889m elevation).  **Shoe:** Own |  | GCT  SF  SL  Maximum pronation velocity  Impact  Braking | SFs and lengths increased while GCT decreased during sprints and 5k race portions. Stroller running increased SF, length and pronation. SL decreased and pronation and GCT increased over the half-marathon race. Wearable sensors allowed for identification of spatiotemporal patterns in running activities encountered in the field setting, providing insight into individuals’ adaptations to external demands of running encountered. |
| DeJong Lempke et al. (2022)  [33] | To prospectively monitor biomechanics, session-rating of perceived exertion (sRPE), and wellness in a cohort of collegiate Division-1 cross-country athletes over the course of a single competitive season. | 21 (9M, 13F)  **Age:**  M: 20.0 ± 1.0  F: 20.0 ± 1.0  **Height:**  M: 176.60 ± 5.13  F: 166.65 ± 5.02  **Weight:**  M: 66.62 ± 6.72  F: 58.43 ± 7.98 | Healthy  Currently participating in varsity cross-country practices  Free of lower extremity musculoskeletal injuries > 3 months | **IMU (3D Accelerometer, 3D Gyroscope):** RunScribe, Scribe Labs Inc.  **SF:** 200Hz  **Number**: 2  **Location:** Shoe (shoelaces) | **Environment:** Outdoor  **Speed:** Self-selected (Long runs: tempo pace (approx. 80% race pace), recovery runs: conversational pace)  **Distance/Time:** 1 long runs/week (M:15-18, F:6-12 miles) + 1 recovery run/week (4-8 miles) x 12 weeks |  | GCT  SF  SL  Pace  Maximum pronation velocity  Pronation excursion  Impact  Braking  Shock | Stride length, GCT, impact g, pace, weekly mileage, and running a meet in the day prior to the recorded run explained 25.4% of the variance in sRPE scores across the season (R2 =0.254, p < 0.001). Contact time and braking g helped explain 3.7% of the variance in wellness (R2 = 0.037, F =5.70, p =0.01). |
| Farina, Needle, and van Werkhoven (2021)  [88] | To evaluate changes in FSP throughout a maximal 800-m run using a conformable IMU attached to the foot | 21 (7M, 14F)  **Age:**  M: 24.71 ± 5.06  F: 24.43 ± 3.92  **Height:**  M: 181.53 ± 7.48  F: 166.83 ± 7.29  **Weight:**  M: 72.27 ± 8.53  F: 58.49 ± 7.06 | Recreational - competitive  Aged 18-35 years  Free of injury > 3-months  Running at least 10 miles per week  Capable of running an 800m run in <4mins  No diabetes, cardiovascular, or renal/kidney disease) and. | **IMU (3D Accelerometer, 3D Gyroscope):** BiostampRC, mc10  **SF:** 250Hz  **Accelerometer Range:** 16g  **Gyroscope Range:** 2000°/s  **Number**: 1  **Location:** Foot (right, dorsum) | **Environment:** Outdoor (Track)  **Speed:** Self-selected (Max. effort)  **Distance/Time:** 800n |  | FSP  FSA | No differences in percentage of RFS throughout the run. Significant differences were seen between curve and straight intervals for average FSA(F [1, 20]= 18.663, p < 0.001, ηp^2^ = 0.483), indicating a more NRFS strike angle on the curves. |
| Florenciano Restoy, Solé-Casals and Borràs-Boix (2021)  [107] | To determine the amplitude of movement differences and asymmetries between feet during the stance phase and to evaluate the effects of foot orthoses on foot kinematics in the stance phase during running | 40 (M)  **Age:** 43.0 ± 13.8  **Height:** 175.5 ± 7.0  **Weight**: 72.0 ± 5.5 | Healthy  Recreational runners  Using foot orthotics for > 1 year  No alterations in their locomotor system | **IMU (3D Accelerometer, 3D Gyroscope, 3D Magnetometer):** MotionPod  **SF:** 30Hz  **Size:** 3.3 x 2.2 x 1.5cm  **Weight:** 14g  **Number**: 2  **Location:** Shoe (instep) | **Environment:** Indoor (Treadmill)  **Speed:** Controlled (2.5 m/s)  **Distance/Time:** 2 (conditions) x 20s  **Shoe:** Own |  | GCT  Step time  Number of steps  Ankle kinematics:  Dorsiflexion/Plantarflexion Abduction/Adduction  Eversion/Inversion | GCT and the total step time show a significant increase when foot orthotics are used, but the number of steps is not altered, suggesting that orthotics do not interfere in running technique. Asymmetry between the extremities when foot orthotics are used is of 5.3 ± 0.8% for dorsi-plantarflexion, 24.8± 16.1% for eversion/inversion and 34.8 ± 18.2% for ab/adduction. No statistically significant differences are present in any case with the condition of the footwear without orthotics (p = > 0.05) |
| Fraeulin et al. (2021)  [108] | To investigate potential differences in running kinematics at the beginning of a transition run and a warm-up run in elite male triathletes by using inertial sensors in overground running. | 16 (M)  **Age:** 32.1 ± 6.0  **Height:** 180.0 ± 7.3  **Weight**: 75.4 ± 7.7 | Healthy  Experienced  Trained to complete the Ironman triathlon in the then-current season sub 9 h 30 min  **Exclusion:** intake of perception-altering substances  Acute injuries or serious diseases that affect the quality of life or physical performance | **IMU (3D Accelerometer, 3D Gyroscope, 3D Magnetometer, Barometer):** Xsens MVN Link  **SF:** 240Hz  **Size:** Sensors & battery: 8.5 x 6.0 x 2.5cm  Body pack: 11.2 x 7.0 x 2.8cm  **Weight:** Sensors & battery: 169  Body pack: 114g  **Number**: 17  **Location:** Suit | **Environment:** Outdoor (Paved)  **Speed:** Controlled (Self-reported long distance triathlon intensity)  **Gradient:** Flat, minimal changes in elevation  **Distance/Time:** 1 (Warm-up run, average analysed 25.21 ± 1.56 m) + 1 (Transition run average analysed 24.98 ± 2.25 m) x 20 steps |  | SF  Step length  Ankle kinematics  Hip kinematics  Knee kinematics  Trunk kinematics | No statistically significant differences between the running conditions were found for the velocity, the stride length, and SF. Transition run; more spinal extension (~0.5–1.0°; p = 0.001) and rotation (~0.2–0.5°; p = 0.001–0.004), increases in hip flexion (~3°; p = 0.001–0.004), internal hip rotation (2.5°; p = 0.001–0.024), more knee adduction (~1°; p = 0.001), and complex altered knee flexion patterns (~2–4°; p = 0.001–0.01) occurred. |
| Glassbrook et al. (2020)  [74] | To quantify the differences in accelerations measured at the dorsal foot compared with the distal tibia during running | 16 (10M, 6F)  **Age:**  M: 23.6 ± 3.7  F: 26.0 ± 5.9  **Height:**  M: 177.0 ± 5.0  F: 169.0 ± 6.0  **Weight:**  M: 78.8 ± 11.4  F: 61.6 ± 6.1 | Healthy  Recreationally active  Aged 18–35 years  Free of injury  Able to run freely, without restriction. | **IMU (3D Accelerometer):** iMeasureU  **SF:** 500Hz  **Size:** 4.0 x 2.8 x 1.5cm  **Weight:** 12g  **Number**: 4  **Location:** Tibia (distal, just above the medial malleolus), Shoelaces | **Environment:** Indoor (Treadmill)  **Speed:** Self-selected (max. sprint)  **Distance/Time:** 1 x 15s sprint (23–29 steps per accelerometer, per trial) |  | Resultant accelerations | Resultant accelerations were greater for left foot than left tibia for 60% of the gait cycle (p < 0.001) and greater for foot right than tibia right for 50% of the gait cycle (p < 0.003). The larger accelerations at the dorsal foot than the tibia can be explained by movement at the ankle joint, and the placement location relative to the hip. Results between the two locations should not be compared. |
| Gregory et al. (2019)  [49] | To evaluate the effects of ankle taping, bracing, and fibular reposition taping (FRT) on running biomechanics as measured with wearable sensors | 12 (6M, 6F)  **Age:** 22.0 ± 1.9  **Height:** 171.2 ± 9.0  **Weight:**76.7 ± 9.3 | Injured  History of at least 1 lateral ankle sprain  Recreationally physically active (20 min of exercise at least 3 times per week)  Capable of running a minimum of 2400 m  **Exclusion:** Ankle sprain < 6 weeks prior  History of ankle surgery or lower-extremity surgery < 12 months prior  Vestibular or neurological disorders, cardiovascular conditions  Inability to complete running requirement  Any condition known to adversely affect gait | **IMU (3D Accelerometer, Gyroscope):** RunScribe, ScribeLab  **SF:** 200Hz  **Number**: 2  **Location:** Shoe (heel) | **Environment:** Outdoor (Track)  **Speed:** Self-selected (Fast speed, RPE 5-6/10)  **Distance/Time:** 4 x 400m  **Shoe:** Own |  | GCT  Stride length  Stride pace  Cycle time  Velocity  Pronation excursion  Maximum pronation  Impact g  Braking g | Ankle taping and bracing were shown to be comparable in decreasing ankle kinematics and kinetics, while fibular reposition taping caused minimal changes in running biomechanics. Taping and bracing may be beneficial in stabilizing and protecting the ankle while fibular reposition taping should not be used to restrict ankle motion during running. |
| Hollis et al. (2021)  [152] | To use the RunScribe™ wearable sensors to analyse kinetic, kinematic and spatiotemporal measures while running on different surfaces and at different speeds | 15 (7M, 8F)  **Age:** 20.0 ± 3.1  **Height:** 170.0 ± 8.1  **Weight:** 65.0 ± 12.3 | Healthy  Recreational  RFS  Run > 10 km per week  Free of musculoskeletal injuries > 12 months  **Exclusion:** History of lower extremity surgery or fracture, neurological or vestibular conditions  Any condition known to adversely affect running gait | **IMU (3D Accelerometer, Gyroscope):** RunScribe, ScribeLab  **SF:** 200Hz  **Number:** 2  **Location:** Shoe (heel) | **Environment:** Outdoor (Grass, Track)  **Speed:** Self-selected (Slow; RPE 3-4. Fast; 5-6)  **Distance/Time:** 2 (surfaces/sessions) x 2 (speeds) 4 x 1600m (slow vs. fast, grass vs. track). Average number of steps analysed per participant was 1353 ± 65 steps for the slow runs and 1236 ± 68 steps for the fast runs.  **Shoe:** Own | Polar T-31 chest strap and an FT1 synchronised watch (Polar Electro) | GCT  Stride Length  Cycle Time  Pronation excursion  Pronation velocity  Impact g  Braking g | RunScribe™ sensors identified changes in running biomechanics measures at different speeds and on varying surfaces. All spatiotemporal, kinematic and kinetic measures were affected by increased running speed and increased surface stiffness (p< 0.050). Significant speed by surface interactions were identified for the measures of stride length (p = 0.018), GCT (p = 0.022), and maximum pronation velocity (p = 0.018). For the measures of cycle time, pronation excursion, impact g, and braking g, there were no significant interactions (p ≥ 0.251), however there were significant main effects for both speed and surface (p ≤ 0.002). |
| Huang et al. (2022)  [109] | Investigate treadmill familiarisation time in different shoe conditions by examining lower limb kinematic waveforms within a gait cycle using the trend symmetry method in recreational runners who used to train on treadmills | 18 (11M, 7F)  **Age:** 26.6 ± 3.3  **Height:** 168.0 ± 8.0  **Weight:** 63.0 ± 18.4 | Healthy  18–45 years old  >3 months of treadmill running experience  Free of lower limb musculoskeletal injuries >3 months  Not undergone any lower limb surgery  No running experience with minimalist or maximalist shoes  **Exclusion:** Cardiopulmonary disease | **IMU (3D Accelerometer, 3D Gyroscope, 3D Magnetometer):** MyoMotion, MyoRESEARCH, 3.14, Noraxon  **SF:** 200Hz  **Number:** 7  **Location:** Pelvis, thigh, tibia, foot (bilaterally) | **Environment:** Indoor (Treadmill)  **Speed:** Self-selected  **Distance/Time:** 3 (shoe conditions) x 10mins  **Gradient:** 0%  **Shoe:** Own & standardised (minimalist: Newton MV2, maximalist: LI-NING ARZN009/6-2) | Intrumented Treadmill (AMTI force-sensing tandem treadmill, 1000Hz) | Sagittal ankle kinematics  Sagittal hip kinematics  Sagittal knee kinematics | Sagittal plane kinematic waveforms in the hip, knee and ankle remained consistent (trend symmetry > 0.95) without extreme excursions (range amplitude ratio ≈ 1) over 10 minutes within each testing shoe condition. Significant time × shoe interaction effect was observed in range offset (i.e., absolute differences in the average degree of kinematic waveforms between consecutive minutes) at ankle (p = 0.029, ŋp 2 = 0.096) and knee (p = 0.002, ŋp 2 = 0.126). Posthoc analysis suggested that running with novel shoes required a shorter time to achieve stable lower limb kinematics (2 to 3 minutes) compared with usual shoes (7 minutes). |
| Jeker et al. (2020)  [82] | To investigate the effects of altitude and distance on uphill vertical speed (VS) and the main spatio-temporal gait parameters during an extreme mountain ultra-marathon | 27 (24M, 3F)  **Age:** 45.3 ± 9.5  **Height**: 176.7 ± 8.0  **Weight:** 72.8 ± 9.0 | Healthy  Experienced (Mountain ultra-marathons) | **IMU (3D Accelerometer, 3D Gyroscope, Barometric pressure):** Physilog® 4 Silver, Gait up SA  **SF:** 100Hz  **Weight:** 19g  **Number**: 1  **Location:** Tibia (lateral malleoli) | **Environment:** Outdoor (Mountain)  **Speed:** Self-selected ( Race pace)  **Altitude:** Low-altitude (1300–2000 m) and a high-altitude (2400–3200 m)  **Distance/Time:** 2 different altitude ranges (low 1300–2000 m vs high 2400–3200 m) of 10 mountains passes distributed over 220 km |  | Stride frequency  Stride height  Vertical speed | Vertical speed was progressively diminished with race progression and this decrease in speed was greater at low than at high altitude. The individual vertical speed change for each uphill portions was more strongly correlated with the changes in SH (r = 0.80, P < 0.001, n = 321) than SF (r = 0.43, P < 0.001, n = 321). Significant interaction (F(4,52) = 4.04, p < 0.01) for the effect of altitude and distance on vertical speed. Suggests a large effect of the knee extensors strength loss on the diminution of vertical speed. |
| Johnson, Outerleys and Davis (2021)  [56] | To examine the correlation between PTAs and GRF loading rates in the medial–lateral and posterior directions. | 18 (10M, 8F)  **Age:** 33.0 ± 11.0  **Height**:173.0 ± 11.0  **Weight:** 72.4 ± 15.4 | Healthy  Recreational  RFS  Run at least 5 miles per week for the previous 6 months  Comfortable running on a treadmill  Free of injury > 3 months  Free of prior surgery or conditions that would affect gait | **IMU (3D Accelerometer):** IMeasureU, Blue Trident  **SF:** 1600Hz  **Accelerometer Range:** 200g  **Number:** 1  **Location:** Tibia (distal-medial, 1 cm above the superior border of the malleolus, right) | **Environment:** Indoor (Treadmill)  **Speed:** Self-selected  **Distance/Time:** 16s (approx. 20 strides each leg)  **Shoe:** Own | Instrumented treadmill (AMTI, 1500 Hz) | Tibial acceleration **Additional:** GRF/Loading rates | Tibial accelerations in the medial–lateral plane seem to be a valid surrogate for the respective GRF measures during running on a treadmill, explaining 74–83% of the variance in loading rates. However, with only 26% of the variance explained, the same may not be true for anterior tibial accelerations and posterior loading rates. |
| Johnson et al. (2020)  [57] | To compare two methods of attaching a skin mounted sensor on mean tibial accelerations, stride-to-stride variability, and correlations with vertical load rates | 18 (10M, 8F)  **Age:** 33.0 ± 11.0 | Healthy  Recreational  RFS  Run at >5 miles/week for the >6 months  Comfortable running on a treadmill  Free of injury >3 months  Free of prior surgery or conditions that would affect gait | **IMU (3D Accelerometer):** IMeasureU, Blue Trident  **SF:** 1600Hz  **Accelerometer Range:** 200g  **Number:** 1  **Location:** Tibia (distal-medial, 1 cm above the superior border of the malleolus, right) | **Environment:** Indoor (Treadmill)  **Speed:** Self-selected  **Distance/Time:** 2 (Attachment methods) x 16s (approx. 20 strides)  **Shoe:** Own | Instrumented treadmill (AMTI, 1500 Hz) | Vertical and resultant tibial acceleration  **Additional:**  GRF  VILR  RILR | Mean vertical accelerations were significantly lower in the wrap condition (p = 0.02, d = 0.57), a more secure attachment method may be necessary for capturing the most representative measure of tibial accelerations during running. No differences were detected in resultant accelerations, vertical loading rates, or stride-to-stride variability. Correlations between tibial accelerations and vertical loading rates were strong (r = 0.79–0.91) and similar between conditions. However, a less secure method (i.e. the strap) is sufficient for capturing tibial accelerations as a surrogate for impact loading forces. |
| Koldenhoven et al. (2020)  [50] | To compare biomechanical measures obtained from wearable sensors as well as lower extremity alignment, range of motion, and strength during running between runners with and those without exercise related lower leg pain. | 32 (16M, 16F)  **Age:**  Healthy: 24.0 ± 4.0, Injured: 23.0 ± 6.0  **Height:**  Healthy: 170.1 ± 8.7, Injured: 171.4 ± 8.4 | Healthy and injured  Recreational runners  Run an average of 10 or more miles (16 km) per week for at least the past 3 months  Aged 18- 45 years.  ERLLP group: pain and tenderness below the knee and above the ankle affecting 5 cm or more of the tibia or cramping or burning pain in the lower leg during or after exercise and currently be experiencing pain during physical activity that had persisted for at least 2 weeks. Healthy runners: visual analogue scale score of <10/100 mm for lower leg pain and no pain with clinical evaluation  **Exclusion:** History of lower extremity surgery < 12 months  Currently experiencing Achilles tendon pain  Had been instructed not to run by a physician  Recent history of fracture  Prolonged period of inactivity  Self-reported any condition known to affect gait.  ERLLP runners were excluded if they had any lower extremity weight-bearing restrictions, a VAS score of .70/100 mm, or were in too much pain to complete the runs in the study. Healthy participants were excluded if they had any other condition that might have interfered with running, such as low back pain, hip pain, knee pain, or another running-related injury, a recent history of ERLLP (within 12 months), or pain with running in general. | **IMU (3D Accelerometer, 3D Gyroscope):** RunScribe, ScribeLab  **SF:** 200Hz  **Number:** 2  **Location:** Shoe (heel) | **Environment:** Outdoor **Speed:** Self-selected **Distance/Time:** Runners wore the sensors for a week, during which time they completed and recorded at least 3 runs, running at their normal pace and usual locations. Healthy: 300684 vs Injured: 270,066 steps analysed.  **Shoe:** Own |  | GCT  SL  Stride length  Stride Pace  Flight ratio  Velocity  Pronation excursion  Maximum pronation  Braking g  Impact g | The injured group had decreased stride length (MD = -0.11 ± 0.05 m, P = .02) and increased GCT (MD = 18.0 ± 8.27 milliseconds, P = .05) versus the healthy group. All other running outcomes were comparable across groups. |
| Meyer et al. (2021)  [59] | To assess the evolution of stride by-stride spatiotemporal parameters, stiffness, and foot strike angle during a marathon and determine possible abrupt changes in running patterns. | 12 (8M, 4F)  **Age:** 36.0 ± 10.0  **Height**:178.0 ± 7.0  **Weight:** 72.0 ± 6.0 | Healthy  Aged > 18 years  Recreational runenrs  Train at least 2x per week  Not suffering from any injury impacting running capacities for the last 6 months | **IMU (3D Accelerometer, 3D Gyroscope, Barometer):** Physilog 5, Gait Up  **SF:** 512Hz  **Accelerometer Range:** 16g  **Gyroscope Range:** 2000°/s  **Number:** 2  **Location:** Shoe (dorsum) | **Environment:** Outdoor **Speed:** Self-selected (race pace)  **Distance/Time:** 26.2 miles (Analysed as 8 x 5km)  **Shoe:** Own | GNSS tracking system | GCT  SF  Stride length  Stride duration  FSA  FT  Swing time  Gait cycle events (initial contact, terminal contact, and mid-stance)  COM oscillation  Peak GRF  Duty factor  Stretch shortening cycle | Running a marathon alters spatiotemporal parameters early in the race, GCT and the duty factor significantly increased during the race (both p < 0.001) while the FT, swing time, stride length, FSA and speed significantly decreased (p < 0.001). All participants maintained a RFS until the end. |
| Moltó et al. (2020)  [36] | To establish the differences between the sexes in terms of lower limb sEMG activity and 3D kinematics of the pelvis during running. | 38 (22M, 16F)  **Age:**  M: 26.4 ± 6.6  F: 27.07 ± 9.6  **Height:**  M: 177.5 ± 0.07  F: 166.3 ± 0.06  **Weight:**  M: 70.1 ± 8.3  F: 58.3 ± 7.1 | Healthy  >90 min/week of running training  Free of injury  **Exclusion:** Reported having suffered an orthopaedic, neurological, or surgical injury < 1 year | **IMU (3D Accelerometer, 3D Gyroscope, 3D Magnetometer):** G-Sensor 2, BTS  **SF:** 4-1000Hz  **Accelerometer Range:** 2, 4, 6, 8, 16g  **Gyroscope Range:** 250, 500, 1000, 2000 °/s  **Number:** 1  **Location:** Lower back (Sacrum (S1), Ergonomic belt) | **Environment:** Indoor (Treadmill, Overground)  **Speed:** Self-selected  **Gradient:** 1%  **Distance/Time:** 1 x 3min  **Shoe:** Own | sEMG (BTS FREEMG 1000, BTS Bioengineering, 1000Hz, 8 muscles) | Gait events (initial contact, toe-off)  Pelvic rotation  Pelvic obliquity  Pelvic tilt  **Additional:**  Muscle activation | Significant differences in the range of pelvic rotation (p = 0.011) were observed between the sexes, with female runners presenting a greater range of rotation during running at their self-selected speed, but no significant differences were observed in the tilt or obliquity between the sexes. |
| Napier et al. (2022)  [23] | To compare the peak acceleration signal associated with RFS, MFS, and FFS strike patterns when measured with an insole-embedded IMU | 187 (98M, 89F)  **Age**: 41.8 ± 12.0 | Healthy  Recreational runners  Free of any musculoskeletal or neurological pain  **Exclusion:** Could not run on a treadmill unaided  Not fit the range of shoe sizes M:8.5–12 US or F:6.5–11 US | **IMU (Accelerometer):** Plantiga Technologies Inc.  **SF:** 500Hz  **Number:** 2  **Location:** Insole | **Environment:** Indoor (Treadmill)  **Speed:** Controlled (2.5, 3.0, 3.5m/s)  **Distance/Time:** 3 (speeds) x 30 strides  **Shoe:** Standardised (New Balance 880v9 or8809v10) |  | Peak vertical, posterior, and resultant accelerations **Additional:**  FSP | 81% of participants exhibited an RFS pattern. RFS pattern was associated with a higher peak resultant (0.29 SDs; p = 0.029) and vertical (1.19 SD; p<0.001) acceleration compared to a FFS pattern. An MFS was associated with the highest peak accelerations in the resultant direction (0.91 SD vs. FFS; p = 0.002 and 0.17 SD vs. RFS; p = 0.091). FFS pattern was associated with the lowest peak accelerations in both the resultant and vertical directions. An RFS was also associated with a significantly greater peak acceleration in the anteroposterior direction (0.28 SD; p = 0.033) than an FFS pattern, while there was no difference between MFS and FFS patterns. |
| Perrotin et al. (2021)  [90] | To (i) measure the variation of spatiotemporal parameters according to the typology of the terrain (ii) in an ecological condition to expose the participants in a variety of trail paths that they could find in competition and (iii) to use so-called “low-cost” portable sensors already used by a large majority of runners | 28 (25M, 3F)  **Age:** 36.0 ± 8.0  **Height**: 175.4 ± 7.2  **Weight:** 68.7 ± 8.7 | Healthy  Recreational - competitive  Aged > 18 years  Participated in a trail competition previously  Free of injury > 6 months | **IMU (3D Accelerometer, 3D Gyroscope):** Stryd, Stryd Inc.  **SF:** 100Hz  **Weight:** 8g  **Number:** 1  **Location:** Shoelaces (right) | **Environment:** Outdoor (Trail/mountains)  **Speed:** Self-selected (race pace)  **Gradient:** 490 m of positive elevation gain  **Distance/Time:** 8.5km  **Shoes**: Own | GPS watch (Suunto 9 Baro-multi sports GPS watch) | GCT  SF  Step length  Step time  FT  Vertical oscillation  Acceleration  Velocity  Force  Distance  Work  Power  Leg stiffness  **Additional:**  Speed  Pace  Climbing speed  Descending speed | GCT was negatively correlated with SF and FT (p < 0.001) over the entire route. Uphill; first ascent, the total ascent time was correlated with the GCT (r = 0.81; p < 0.001). Average speed correlated with the SL and FT was positively correlated with the runner’s SF (r = 0.69; p < 0.001). Second ascent, ascent time was negatively correlated SL, SF, power, and speed (p < 0.001). Downhill; GCT that showed a positive correlation whereas SL showed a negative correlation with the total descent time (p < 0.001). In the first descent, the FT was correlated with SL (r = 0.80; p < 0.001). |
| Prigent et al. (2022)  [89] | To investigate the concurrent evolution of running biomechanics and heart rate dynamics in response to perceived fatigability for recreational runners, using body-worn smartphone, IMU, GNSS and ECG sensors | 13 (11M, 2F)  **Age:**  Group 1: 35.5 ± 9.3  Group 2: 35.6 ± 5.8 | Healthy  Recreational runners | **IMU (3D Accelerometer, 3D Gyroscope):** Fieldwiz, ASI & Physilog 5, Gaitup SA  **SF:** Fieldwiz: 200Hz Physilog: 512Hz  **Accelerometer Range:** Physilog 16g  **Gyroscope Range:** Physilog: 2000 °/s  **Number:** 2  **Location:** Fieldwiz: Chest, Physilog: Foot | **Environment:** Outdoor **Speed:** Self-selected (race pace)  **Distance/Time:** 13.1 or 26.2 miles |  | GCT  FSA  FT  Swing time  Gait cycle time  Peak swing velocity of the foot  Foot eversion angle  Vertical stiffness  Duty factor | The GCT, duty factor, and trunk AP acceleration values significantly increase during the race (p<0.001), FSA (p<0.001) and vertical stiffness (p<0.05) significantly decrease. GCT, duty factor, trunk AP acceleration (all p < 0.001) and vertical stiffness (p < 0.05) show significant changes at all fatigue states. A significant decrease of peak swing vel. appears at moderate fatigue states and FSA and swing time values became significantly lower only at high fatigue scores. |
| Provot et al. (2019)  [77] | Assess the effect of running speed on several indicators derived from the acceleration measurement, based on three specific body locations | 18 (10M, 8F)  **Age:** 31.4 ± 8.9  **Height:** 172.0 ± 9.0  **Weight:** 64.9 ± 12.3 | Healthy  Recreational runners  Minimum training frequency of 2 x/week  Met at least one of the following criteria: 10 km < 55 min, or 13.1 miles in <1hr50min | **IMU (3D Accelerometer):** Hikob  **SF:** 1344Hz  **Accelerometer Range**: 24g  **Size:** 4.5x 3.6 x 1.7cm  **Weight:** 22g  **Number:** 3  **Location:** Lower back (L4-L5), Tibia (COM of leg - protruding part (midshaft)), Shoe (dorsal surface of the right shoe above the metatarsals) | **Environment:** Indoor (Treadmill)  **Speed:** Controlled (8 - 18 km/hr)  **Distance/Time:** 9 x 30s (60s trials)  **Shoe:** Standardised (Kalenji®, Ekiden One) |  | Mean contact duration  Mean stride frequency  Mean flight duration mechanical & physiological  RMS  Spectral energy  mechanical & physiological  Mechanical leg stiffness  Stable leg stiffness  Median frequency for stable contact | Raw signal indicators related to the energy of the signal (RMS and Spectral energy) presented an accuracy of 100% for each case. Indicators were considered linear compared to speed with r2m > 0.90. Complete stride: good linearity (r2m > 0.93) with perfect accuracy (100%). Mean contact duration (mechanical) had acceptable linearity (r2m= 0.89) and excellent accuracy (100%); Mean flight duration (mechanical) seemed to be independent of speed (p-value = 0.172). Mean contact duration physiological, Mean flight duration physiological showed poor linearity (r2m =0.55) and accuracy ( > 70%). |
| Reenalda et al. (2019)  [19] | Examine the effects of a prolonged run on shock attenuation, PTA and peak sacral acceleration (PSA), and lower limb kinematics using IMUs | 10 (M)  **Age:** 31.0 ± 5.0  **Height:** 183.0 ± 3.0  **Weight:** 76.0 ± 9.0 | Healthy  Male  Well-trained runners  Run > 40 km per week  Free of musculoskeletal injury >6 months | **IMU (3D Accelerometer, 3D Gyroscope, 3D Magnetometer):** MTx, Xsens Technologies  **SF:** 100Hz  **Accelerometer Range:** 18g  **Gyroscope Range**: 1200 °/s  **Weight:** 30g  **Number:** 8  **Location:** Sternum, Lower back (sacrum), Thigh (lateral at the iliotibial tract), Tibia (anteromedial part of the tibia), Shoelaces (bilaterally) | **Environment:** Outdoor (Track)  **Speed**: Controlled (Estimated lactate threshold speed -estimated using each participants’ 10 km seasonal best)  **Distance/Time:** 40 strides (20 at beginning and at end, on the straights of the track) **Shoe:** Own |  | Peak joint angle in the sagittal plane at initial contact and midstance  PTA  Peak sacral acceleration  Shock attenuation | PTA increased significantly (p<0.05) while peak sacral acceleration and shock attenuation did not change following the prolonged run. Hip and knee flexion at midstance decreased significantly. (p < 0.05). Knee flexion increased at initial contact and decreased significantly at midstance between the beginning and end of the run (p < 0.05). Vertical lower leg angle at initial contact did not change. By using IMUs, it was shown that a prolonged run at estimated lactate threshold speed had significant effects on kinematics and tibial acceleration parameters. |
| Reenalda et al. (2020)  [20] | To present a measurement set-up based on IMUs, to perform a continuous 3D kinematic analysis of running technique during the course of an actual marathon to objectify changes in running mechanics. | 3 (M)  **Age:** 38.7 ± 8.2  **Height:** 182.0 ± 2.4  **Weight**: 73.0 ± 3.7 | Healthy  Experienced runners Expected marathon finish time around 3h  Free of musculoskeletal injury >1 year | **IMU (3D Accelerometer, 3D Gyroscope, 3D Magnetometer):** MTx, Xsens Technologies  **SF:** 60Hz  **Gyroscope Range:** 1200°/s  **Size:** 3.45 x 5.78 x 1.45cm  **Weight:** 27g  **Number:** 8  **Location**: Sternum, Lower back (sacrum), Thigh (lateral at the iliotibial tract), Tibia (anteromedial part of the tibia), Shoelaces (bilaterally) | **Environment:** Outdoor (Asphalt)  **Speed:** Self-selected (Race pace)  **Distance/Time**: 4 stages x 100 strides (approximately at 8, 18, 27, 36 km) | GPS enabled watch (Garmin Forerunner 210, Garmin) Measuring distance and velocity | SF  SL  Vertical oscillation  Peak ankle angle at initial contact  Peak hip and knee joint angles during mid-stance and mid-swing | Running velocity decreased significantly for one runner (p<0.001) while stride length decreased in two runners and increased in one runner between the first and last stage of the marathon (p<0.001). SF decreased in one runner and increased in two runners between the first and last stage (p<0.001). Significant changes in running mechanics were observed between the first and the last stage of the marathon. |
| Strohrmann et al. (2012)  [21] | To monitor kinematics by extracting kinematic parameters from motion data collected using wearable sensors. To demonstrate the potential of wearable technology for monitoring running kinematics in the field with a study including two exhausting runs: one on a treadmill and one on a conventional track | 21 | Healthy | **IMU (3D Accelerometer, 3D Gyroscope, 3D Magnetometer):** ETHOS  **SF:** 100Hz  **Accelerometer Range**: 6g  **Gyroscope Range**: 2000°/s  **Weight:** 27g  **Number:** 12,  **Location:** Wrist, Upper back, Lower back, Thigh, Tibia, Shoelaces (bilaterally) | **Environment:** Indoor (Treadmill), Outdoor (Track)  **Speed:** Controlled (85% max. speed)  **Distance/Time:** 2 (environments/days) x 45 min | Video recordings | SF  FSP  Vertical oscillation  Normalised foot contact  Maximum knee rotation velocity  Arm movement, Impact acceleration on upper body  Trunk forward leaning  Heel lift  Shoulder rotation | Kinematic changes during treadmill running were not consistent with those observed during the over ground runs. On the treadmill, runners lowered their SF as they fatigued. Less experienced runners changed their running kinematics more than experienced runners (increased GCT with fatigue, more vertical oscillation on the treadmill, higher impact acceleration on upper body). Changes in kinematics can be categorized in 3 groups: 1) changes that occurred for all runners such as the decrease of the heel lift and increased trunk forward lean; 2) changes that depended on the runner’s skill level (e.g., increased GCT); 3) and changes that were highly dependent on the individual, (e.g., increased shoulder rotation). |
| Tenforde et al. (2020)  [52] | To examine the relationship between tibial acceleration and loadrates in injured runners who are RFS, MFS, and FFS strikers. | 169 (95M, 74F)  **Age:** 38.7 ± 13.1  **Height:** 172.0 ± 9.0  **Weight:** 70.4 ± 12.0 | Previously injured  Habituated to neutral cushioned shoes  **Exclusion:** Pain greater than 2 out of 10 on a numerical rating scale during the testing | **IMU (3D Accelerometer):** IMeasureU  **SF:** 1000Hz  **Size:** 4.0 x 2.8 x 1.5cm  **Weight:** 12g  **Number:** 1  **Location:** Tibia (distal medial portion above the medial malleolus, left) | **Environment:** Indoor (Treadmill)  **Speed:** Self-selected  **Distance/Time:** 1 x 16s/8 consecutive strides (3 min trial)  **Shoe:** Own | Video camera (125Hz)  Instrumented treadmill (AMTI, 1500Hz) | Vertical and resultant tibial acceleration | Loadrates were each associated with vertical tibial acceleration (r = 0.66-0.82, P < .001) and resultant tibial acceleration (r = 0.41-0.68, P < .05) across all FSPs with the exception of association of vertical instantaneous loadrates to resultant tibial acceleration in the FFS group. The strength in correlations was lowest between resultant tibial acceleration and loadrates for the FFS runners (r = 0.41-0.47, P < .05). Vertical tibial acceleration is the stronger surrogate for loadrates in injured runners across differing FSPs. |
| Wunsch et al. (2017)  [70] | To investigate whether LEAF shoes alters spatiotemporal variables (SL and stride rate) and running economy in over ground running compared to a standard midsole shoe (FOAM). To examine whether midsole design-induced alterations in spatiotemporal variables are related to running economy. | 10 (M)  **Age:** 33.1 ± 7.1  **Height:** 178.0 ± 5.0  **Weight:** 73.0 ± 6.8 | Healthy  Recreational runners  RFS>20km/week  >8 years of running experience  Free of injury >1 year  Shoe size US 9  No history of cardiovascular or neurological disorders | **IMU:** Humotion  **SF:** 400Hz  **Size**: 6 x 1.8 x 0.6  **Weight:** 5g  **Number:** 1  **Location:** Ankle (right) | **Environment:** Outdoor (Track)  **Speed:** Controlled (Running speed at 2 mmol/l blood lactate)  **Distance/Time:** 4 (2 runs in 2 shoe conditions) x 15min. (>1800 stride per shoe condition)  **Shoe:** Standardised (Leaf spring-structured midsole shoe and standard foam shoe) | 2D Video Analysis (50Hz) | Stride rate  Stride length  **Additional:**  FSP | Due to the shoe mechanics of the LEAF on average an increase in SL and reduction in stride rate were found in over ground running. Furthermore, the alterations of these spatiotemporal variables were correlated to running economy. The response to the structured midsole concept was highly individual. |
| *Accelerometers* |  |  |  |  |  |  |  |  |
| Boey et al. (2017)  [118] | To look at the influence of running surface, running speed (comfortable running and imposed speed), and running experience on vertical acceleration of the tibia. | 35 (18M, 17F)  Untrained (6M, 6F)  Recreational (6M, 6F)  Well-trained (6M, 5F)  **Age:**  Untrained: 22.3 ± 1.8  Recreational: 22.3 ± 2.5  Well-trained: 25.4 ± 5.0  **Height:**  Untrained: 173.2 ± 8.9  Recreational: 176.3 ± 9.2  Well-trained:178.0 ± 7.9  **Weight:**  Untrained: 65.0 ± 9.1  Recreational: 65.8 ± 8.1  Well-trained: 63.3 ± 5.1 | Healthy  Free of injury > 3 months  Untrained: no running experience and < 2 hours/week  Recreational: ran between 10 and 30 km/week for > 6 months  Well-trained: ran > 50 km per week under supervision of a coach for > 2 years | **3D Accelerometer:**  X-50-2, Gulf Coast Data Concepts  **SF:** 1024Hz  **Accelerometer Range:** 50g  **Weight:** 33g  **Number:** 1  **Location:** Tibia (8 cm above the medial malleolus, right) | **Environment:** Outdoor (Concrete, Track, Woodchip)  **Speed:** Controlled and self-selected (3.06 m/s)  **Distance/Time:** 6 x 70m (2 on each surface, 90m in total - first and last 10m excluded) | Timing gates (Kit Racetime2 Light radio) | PVAP | Vertical acceleration was significantly lower during running on the woodchip trail in comparison with the synthetic running track and the concrete, and significantly lower during running at lower speed in comparison with during running at higher speed on all surfaces. No significant differences in vertical acceleration were found between the three groups of runners at fixed speed. Higher self-selected speed due to higher performance level also did not result in higher vertical acceleration. |
| Burns, Zendler and Zernicke (2019)  [31] | Recorded the SF patterns of highly trained ultramarathon runners in an elite-level competition and explored the effects of fatigue, pace, and intrinsic characteristics on SF | 20 (12M, 8F)  **Age:** 38.1 ± 6.4  **Height:** 172.0 ± 8.0  **Weight:** 60.0 ± 7.9 | Healthy  Elite runners; Finished in top 25 male or female participants  Continuously used a recording wrist-worn device with an embedded accelerometer that reported step count during the entire competition | **Accelerometer (GPS Watch):** 12 x Garmin, 7 x Suunto, 1 x Polar  **SF:** 1 to 6 s across devices  **Number:** 1  **Location:** Wrist | **Environment:** Outdoor (Concrete)  **Speed**: Self-selected (Race pace)  **Distance/Time:** 100km (10 x 10km laps) |  | SF | After control for all other factors, SFs and SF variability of the individual runners were significantly affected by their running speed, with faster speeds eliciting higher SFs and lower SF variability. Stature also had a significant effect on SF, with taller runners having lower SFs, however no effect on SF variability. SFs of runners did not significantly change across 100 km of running. Sex, age, weight, or years of running experience did not exert a significant effect on SF or SF variability. Distance had a significant effect on the SF variability, as within-lap variability decreased throughout the race. |
| Butler, Hamill and Davis (2007)  [44] | Evaluate changes in kinematics and kinetics over the course of a prolonged run when low (LA) and high (HA) arched runners wear motion control and cushioning shoes. | 24  **Age:**  HA: 20.9 ± 3.0  LA: 21.8 ± 3.2  **Height:**  HA: 170.0 ± 7.0  LA: 173.0 ± 11.0  **Weight:**  HA: 68.4 ± 5.8  LA: 70.4 ± 7.3 | Healthy  Recreational runners  Aged 18-40 years  Running > 10 miles/week  Subjects were classified as HA if they were at least 1.5 S.D. above the population mean Arch Height Index value and as LA is they were at least 1.5 S.D. below the population mean  **Exclusion:** History of cardiovascular problems  Any lower extremity condition that would affect their gait pattern | **1D Accelerometer:**  PCB Piezotronics  **SF:** 1080Hz  **Weight:** 2.83g  **Number:** 1  **Location:** Tibia (distal anteromedial) | **Environment:** Indoor (Treadmill)  **Speed:** Self-selected (training pace (they would use for a 30–45 min run))  **Distance/Time:** HA: 52 ± 25 min  LA: 47 ± 24  **Shoe:** Standardised (New Balance 1122 (motion control shoe) and the New Balance 1022 (cushioning shoe)) | 3D Motion Capture (Vicon Motion Systems Ltd, 120Hhz). | PPA  Peak to peak tibial acceleration  **Additional:**  Peak eversion velocity  Rearfoot eversion excursion Peak tibial internal rotation  Tibial internal rotation excursion | Low arch: Peak tibial internal rotation decreased in the motion control shoe and was increased in the cushioned over the course of the run. No interactions or main effects were noted for peak eversion or eversion excursion. High arch: No shoe by time interaction was observed for tibial shock. Main effect for shoe, with lower tibial shock associated with the cushioned shoe. |
| Clermont et al. (2019)  [32] | To classify runners in sex-specific groups as either competitive or recreational based on features extracted from a 3D accelerometer positioned near the COM, and to compare the classification accuracies to a group model that included both males and females | 41 (25M, 16F) (M: 10 competitive and 15 recreational. F: 7 competitive and 9 recreational)  **Age:**  Competitive: 35.0 ± 12.5,  Recreational: 30.8 ± 12.37  **Height:**  Competitive: 180.7 ± 5.6  Recreational: 177.5 ± 7.1  **Weight**:  Competitive: 75.9 ± 3.9  Recreational: 74.2 ± 8.4 | Healthy  Competitive: age-graded score  ≥60 %  Recreational: never participated in a race or had recently participated in a middle- to long-distance race and achieved an age-graded score <60 %  Free of injury | **3D Accelerometer:** Shimmer3, Shimmer Inc  **SF:** 201.03Hz  **Accelerometer Range:** 8g  **Number:** 1  **Location:** Lower back (L3 - L5 vertebrae, near COM, elastic straps) | **Environment:** Indoor (Treadmill)  **Speed:** Self-selected (Preferred)  **Distance/Time:** 4m30s (5 mins in total, 1st and last 15s removed)  **Shoe:** Own |  | StepCV  StrideCV  REGSTEP_ML  REGSTEP_V  REGSTEP_AP  REGSTRIDE_ML  REGSTRIDE_V  REGSTRIDE_AP  SYMML  SYMV  SYMAP  PEAKML  PEAKV  PEAKAP  RMSML  RMSV  RMSAP  RMSR  RATIOML  RATIOV  RATIOAP  RMSCVML  RMSCVV  RMSCVAP | Competitive and Recreational runners could be classified with 82.63 % and 80.4 % in the male and female models, respectively. Dominant features in both models were related to regularity and variability, with competitive runners exhibiting more consistent running gait patterns, but the specific features were slightly different in each sex-specific model. |
| Encarnación-Martínez et al. (2021)  [35] | To analyse impact accelerations, spatiotemporal parameters and perceptual differences while running on curved non-motorized treadmill compared to motorized treadmill at different speeds. | 27 (22M, 5F)  **Age:** 25.0 ± 7.0  **Height:** 170.3 ± 8.1  **Weight:** 64.4 ± 10.3 | Healthy  Physically active (run > 2x/week in the last year)  Training volume of > 20 km/week  Free of lower limb injury for > 6 months  No suffering of heart failure, neurological or musculoskeletal disorders affecting normal locomotion and to not be taking medication that interferes with stability during running  **Exclusion:** Injury, surgery, or illness within the previous 6 months  Overweight or obese | **3D Accelerometer:**  Pikkulab, Blautic Design  **SF:** 180Hz  **Accelerometer Range:** 16g  **Size:** 0.5 x 0.2 x 0.1cm  **Weight:** 50g  **Number:** 2  **Location:** Head (anterior), Tibia (distal anteromedial aspect) | **Environment:** Indoor (Treadmill)  **Speed:** Controlled & self-selected (2.77 & 3.33 m/s)  **Gradient:** 1%  **Distance/Time:** 2 (sessions) x 3 (speeds) x 8 mins  **Shoe:** Own |  | SF  Stride length  Tibial acceleration  Tibial acceleration rate  Tibial acceleration magnitude  Head acceleration  Head acceleration rate  Head acceleration magnitude | Spatiotemporal parameters were not significantly different between treadmills in any of the conditions of the study (p > 0.05). A reduction in impact accelerations, head acceleration rate (mean ES = 0.86), tibia peak (mean ES = 0.45) and tibia magnitude (mean ES = 0.55), was observed while running on curved non-motorised treadmill in comparison with running on motorised treadmill. Stride length increased as velocity was higher, finding significant (p < 0.05) differences between self-selected speed and 3.33 m/s (ES = 1.723), and between 2.77 m/s and 3.33 m/s (ES = 2.777). No significant differences (p > 0.05) in stride frequency when comparing running velocities. Impact accelerations were significantly higher (p < 0.05) when running at 3.33 m/s. |
| Garcia, Gust and Bazett-Jones (2021)  [131] | To investigate peak triaxial and resultant tibial acceleration as well as axial and resultant shock attenuation among dirt, gravel, and paved surfaces | 15 (3M, 12F)  **Age:** 27.7 ± 9.1  **Height:** 165.0 ± 6.0  **Weight:** 65.9 ± 13.1 | Healthy  Free of injury  >30 mins of activity 5 days/week, with 3 days of weekly running over the past 3 months  **Exclusion:** History of injury < 6 months | **3D Accelerometer:**  TeleMyo DTS, Noraxon  **SF:** 1500Hz  **Accelerometer Range:** 24g  **Weight:** 28g  **Number:** 2  **Location:** Head (anterior), Tibia (right, distal anteromedial aspect) | **Environment:** Outdoor (Dirt, gravel, paved)  **Speed:** Self-selected  **Gradient:** <10°  **Distance/Time:** 3 (surfaces) x 4 (trials) x 30m  **Shoe:** Standardised (Saucony Jazz) |  | SF  Peak tibial braking, propulsion, axial, medial, lateral, and resultant acceleration  Shock attenuation | No significant differences were found between the average number of strides analysed (p=0.99), stride rate for each running surface (p=0.83), tibial acceleration variables (p = 0.85 - 0.95) or shock attenuation variables (p=0.87 - 0.90). |
| Howe et al. (2021)  [132] | To investigate the changes in metabolic variables, running energetics and spatiotemporal gait parameters during an 80.5 km treadmill ultramarathon and establish which key predictive variables best determine ultramarathon performance. | 12 (9M, 3F)  **Age:** 34.0 ± 7.0  **Height:** 173.7 ± 7.3  **Weight:** 68.4 ± 7.4 | Healthy,  Endurance runners  > 3 years running experience  Free of injury | **3D Accelerometer:**  GT3X+, ActiGraph  **SF:** 100Hz  **Number:** 1  **Location:** Hip (dominant, mid-axillary line) | **Environment:** Indoor (Treadmill)  **Speed:** Controlled & self-selected (8km/hr & max. effort)  **Distance/Time:** 80.5km |  | SF  SL | No significant change in the spatiotemporal parameters measured across all 16.1 km measurement splits at the control speed of 8 km/hr; SF (F (2.02, 22.3) = 1.76, p = 0.195, ηp^2^ = 0.14, observed power = 0.33), and SL (F (2.4, 26.3) = 2.28, p = 0.11, ηp^2^ = 0.17, observed power = 0.46) at the control speed. No significant correlation between ΔSL (%) and ΔSF (%) at self-selected running speed when compared to ΔEnergy cost of running (%) (r = − 0.23; p = 0.48 and r = 0.02; p = 0.95, respectively) |
| Lucas-Cuevas et al. (2017)  [119] | To analyse the acute differences in stride and shock parameters while running on a treadmill with custom-made and prefabricated insoles | 38 (20M, 18F)  **Age:** 29.8 ± 5.3  **Height:** 170.3 ± 11.4  **Weight:** 65.4 ± 10.1 | Healthy  > 20 km/week  Free of injury > 1 year  No lower-limb surgery > 3 years  No previous use of insoles | **3D Accelerometer:**  Sportmetrics  **SF:** 500Hz  **Size:** 4.0 x 2.2 x 1.2cm  **Weight:** 2.5g  **Number:** 2  **Location:** Head (anterior), Tibia (proximal anteromedial aspect) | **Environment:** Indoor (Treadmill)  **Speed:** Controlled (3.33 m/s)  **Distance/Time:** 2 (sessions) x 4 (pre-fatigue x 2 insoles, post-fatigue x 2 insoles) x 60s  **Shoe:** Own, standardised insoles (prefabricated insoles (Tecnoped Run, Herbitas), custom-made insoles (OPCT Run, Sidas S.L.)) |  | Stride frequency  Stride length  PTA  Head acceleration  Acceleration magnitude  Acceleration rate  Shock attenuation | Stride rate and stride length were not influenced by the intense run (p > 0.05). Different insoles did not influence stride rate and stride length (p > 0.05). Insole conditions affected the shock accelerations during running; Pre-fatigue state, the use of custom-made insoles reduced tibial acceleration rate (p = 0.014, mean difference: 85.38, 95%CI mean difference: 14.56±156.20) compared to the prefabricated insoles. 95%CI mean difference: 0.56±13.38). No difference was observed between the custom-made and the control insoles for any of the parameters analysed (p > 0.05). |
| McNair and Marshall (1994)  [58] | To examine the acceleration and energy absorption characteristics associated with material testing of four shoes with different shock attenuating materials and design concepts, and then to relate these findings to tibial accelerations and sagittal plane kinematics measured during running in these same shoes. | 10 (M)  **Age:** 25.0 ± 5.0  **Weight**:75.0 ± 6.0 | Healthy  Recreational runners  RFS | **Accelerometer:** PC9367/20, Phillips  **SF:** 1000Hz  **Number**: 1  **Location:** Tibia (lower medial aspect) | **Environment:** Indoor (Treadmill)  **Speed:** Controlled (3.5 m/s)  **Distance/Time:** 5 x 5min (8 strides digitised)  **Shoe:** Standardised (double density EVA with a cantilever outsole; double density EVA; air filled chambers within a double density EVA midsole; encapsulated double density EVA) | Motion analysis (Motion analysis flextrack system)  Video data (200 fps) | PTA  Time to peak acceleration **Additional:**  Knee kinematics | No significant differences (p > 0.05) were observed in the peak acceleration and time to peak acceleration during running in shoes, these variables were significantly greater in the barefoot running condition (p < 0.05), as compared with running in shoes. Small and subtle kinematic differences were observed between the barefoot and shoe conditions. Runners prefer to operate within an impact acceleration 'bandwidth' as evidenced by the similarity of tibial accelerations across shoes, and by subtle but distinct differences in the ankle and knee kinematics. |
| Meardon, Hamill and Derrick (2011)  [48] | To evaluate the effects of a prolonged run on stride time variability. | 18 (M/F)  **Age:**  No Injury: 25.9 ± 8.5  Injury: 29.3 ± 10.3  **Height:**  No Injury: 170.2 ± 10.9  Injury: 170.6 ± 9.3  **Weight:**  No Injury: 62.6 ± 8.3  Injury: 66.3 ± 7.8 | Healthy and injured  Free of injury  Injured: history RRI of the lower extremity that prohibited running for > 1 week | **1D Accelerometer:** ADXL250, Analog Devices  **SF:** 1000Hz  **Number**: 1  **Location**: Tibia (distal anteromedial) | **Environment:** Indoor (Track)  **Speed:** Controlled (5 km race pace (self-reported) + 5%)  **Distance/Time:** 1 x max distance at consistent speed (Average: 19 ± 3 laps of 300m track, average number of strides: 661) |  | Stride time  PTA | Significant linear trend in alpha for interval occurred with a reduction in alpha over the course of the run (p = 0.01) indicating that over the run, stride times of runners became more unpredictable - likely due to movement errors associated with fatigue necessitating frequent corrections. Injured group exhibited lower alpha (M = 0.79, CI95 = 0.70, 0.88) than the non-injured group (p = 0.01) (M = 0.96, CI95 = 0.88, 1.05); a reduction hypothesized to be associated with altered complexity. |
| Milner, Hawkins and Aubol (2020)  [60] | To determine whether laboratory and field measures of tibial acceleration are comparable, and whether peak axial and peak resultant tibial acceleration are interchangeable | 19 (9M, 10F)  **Age:** 31.0 ± 6.0  **Height:** 170.0 ± 8.0  **Weight:** 68.6 ± 11.6 | Healthy  Recreational runners  RFS  Run > 10 miles/week for > 1 year  Free of injury  **Exclusions**: History of major lower extremity injury or surgery | **Accelerometer:** 356A45, PCB Piezotronics  **Number:** 1  **Location:** Tibia (distal, right) | **Environment:** Indoor (Treadmill, Track), Outdoor (Concrete, Grass)  **Speed:** Controlled (3 m/s)  **Distance/Time:** 4 (conditions) x 10-foot strikes  **Shoe:** Own | 3D Motion Capture (8 camera Vicon T40S system, 200Hz)  Force plates (AMTI, 1000Hz)  2 Photocells (Brower)  GPS Watch (Garmin 735XT, Garmin record velocity and SF) | Peak positive axial acceleration  Peak resultant acceleration  Peak medial, lateral, anterior, and posterior acceleration  Angles of inclination of the peak resultant vector  Percent stride to peaks | PPA was lower in laboratory and treadmill compared with grass and sidewalk conditions. Laboratory and treadmill were similar in magnitude, as were grass and sidewalk. Peak resultant acceleration was consistently higher than peak axial acceleration, with the same pattern among conditions. Laboratory acceleration measures explained at best only half of the variance in the field conditions and did not explain the variance for grass. Tibial impact acceleration magnitude is influenced by testing procedures in runners. |
| Mizrahi et al. (2000)  [111] | Investigate the effects of fatigue in long distance running on several kinematic parameters of the leg, including stride rate, knee angle in the sagittal plane and hip vertical excursion in view of the significant increase in the impact acceleration on the shank | 14 (M)  **Age:** 24.2 ± 3.7  **Height**: 175.5 ± 5.9  **Weight**: 73.2 ± 8.3 | Healthy  No previous histories of muscle weakness, neurological disease, or drug therapy | **Accelerometer**: 8634B50, Kistler PiezoBea  **SF:** 1667Hz  **Weight:** 4.2g  **Number**: 1  **Location:** Tibia (tibial tuberosity, right) | **Environment:** Indoor (Treadmill)  **Speed:** Controlled (Steady speed exceeding the anaerobic threshold level of each subject by 5% and lasted 30 minutes  **Distance/Time:** 27 Heel strikes (30 minutes, acceleration data acquired every 5 minutes for a time span of 20s)  **Shoe:** Standardised | 2D Video Analysis (NV-M3000EN Panasonic camera (50frames/second)) | Stride rate (times between the impact accelerations)  Impact acceleration on the shank  **Additional:**  Hip and knee kinematics | From the 1st to the 30th minute of running: average stride rate decreased from 1.46 ± 0.05 to 1.39 ± 0. 03 seconds; The average impact acceleration on the shank increased from 6.9 ± 2.9 to 11.1 ± 4.2 g. Kinematic changes due to fatigue were consistent with the substantially higher impact accelerations, increasing the risk of overload injuries in the shank. |
| Montgomery et al. (2016)  [46] | To examine tibial acceleration and muscle activation during overground, motorised treadmill and non-motorised treadmill conditions when walking, jogging and running at matched velocities | 15  **Age**: 24.2 ± 3.8  **Height**: 179.5 ± 3.9  **Weight:** 81.0 ± 7.2 | Healthy  Recreationally active | **3D Accelerometer:** DTS 3D accelerometer, Noraxon  **SF:** 1500Hz  **Accelerometer Range**: 16g  **Number:** 1  **Location:** Tibia (mid-anterior, right) | **Environment:** Indoor (Treadmill, Overground)  **Speed:** Self-selected; Jogging, running (and walking)  **Distance/Time:** 6 (3 jogging, 3 running) x 8 cycles (Additional 3 walking trials)  **Shoe:** Standardised (Umbro 5v5) | Speed Meter (Speed Real Time, AP Lab)  Surface EMG electrodes (Ambu Blue Sensor N, Ambu, Cambridgeshire, UK) | Cycle time  Tibial acceleration peak  Acceleration gradient **Additional:**  Muscle activation | The largest acceleration peaks and gradients were seen in running trials, followed by jogging then walking. Non-motorised treadmill produced large reductions in acceleration peaks compared to over ground and motorised conditions across conditions but had no effect on acceleration gradients across conditions. The non-motorised treadmill condition generated large and very large decreases in cycle time compared to other conditions during jogging and very large decreases during running. Overground and motorised treadmill conditions produced similar cycle times. |
| Pla et al. (2021)  [51] | To determine how movement dynamics alter in response to fatigue, and the possible link with developing lower-leg overuse injuries during a six-month follow-up period | 132 (96M, 42F)  **Age**: 18.7 ± 2.0  **Height:** 176.0 ± 8.0  **Weight**: 67.8 ± 9.1 | Healthy  Recreational runners | **Accelerometer:** Byteflies  **SF:** 1000Hz  **Accelerometer Range**: 16g  **Weight:** 5g  **Number**: 1  **Location:** Lower back (L3-5) | **Environment:** Outdoor (Track)  **Speed:** Self-selected  **Distance/Time:** 12 mins  **Shoe:** Own |  | GCT  SF  Impact acceleration  RMS ratio  Step regularity  Stride regularity | No significant differences between injured and non-injured groups were observed for GCT and SF (p > 0.05). Significant differences between the groups were observed for AP impact acceleration, AP and vertical RMS ratio (p < 0.05). |
| Sheerin, Besier and Reid (2020)  [137] | To investigate the impact of increasing running velocity on resultant PTA in injury-free runners. | 85 (65M, 20F)  **Age**: 39.6 ±9.0  **Height:** 176.0 ± 9.0  **Weight**: 73.9 ± 11.0 | Healthy  Run > 3x /week and > 20km for > 6 months  Free of injury | **3D Accelerometer:** IMeasureU  **SF:** 1000Hz  **Accelerometer Range**: 16g  **Weight:** 12g  **Number**: 2  **Location:** Tibia | **Environment:** Indoor (Treadmill)  **Speed:** Controlled (2.7, 3.0, 3.3, 3.7 m/s)  **Distance/Time:** 4 x 50s (2 min trials)  **Shoe:** Standardised (Asics Kudrow) | Instrumented treadmill (Bertec) | Peak tibial accelertaion  Symmetry angle | Tibial acceleration increased with higher velocities, with an average increase of 3.8 g (38%) between the slowest and fastest speeds. Moderate correlation was demonstrated between tibial acceleration and running velocity, and 19% of tibial acceleration was explained by velocity. While velocity influences tibial acceleration, individual variances to this relationship exist. |
| Shun-Ping et al. (2014)  [30] | To use a triaxial accelerometer to compare the 3D COM accelerations of two groups of ultramarathon runners with distinct performances during different running speeds and distances | 10 (M)  **Age:** 50.3 ± 9.4  **Height:** 166.1 ± 6.0  **Weight:** 59.5 ± 5.2 | Healthy  Male  Members 12-h Taipei International Ultramarathon Race team  Free of injury and disease | **3D Accelerometer:** ADXL330, Analog Devices  **SF:** 500Hz  **Size:** 5.2 x 3.2 x 0.6 cm  **Weight:** 10g  **Number:** 1  **Location**: Lower back (Height of the COM, intersection where the anterior superior iliac spine line connected with the vertebrae) | **Environment:** Indoor (Treadmill)  **Speed:** Controlled (3, 6, 8, 9, and 12 km/hr)  **Distance/Time:** 5 x 60s (5 min trials)  **Shoe:** - |  | COM acceleration | Elite group showed negative correlations between mediolateral acceleration (r = −0.83 to −0.93, p < 0.05), and between anterior–posterior acceleration and running distance (r = −0.8953 to −0.9653, p < 0.05), but not for vertical control of the COM. Runners reduce stride length to minimize mediolateral sway and the effects of braking on the trunk; moreover, cadence must be increased to reduce braking effects and enhance impetus |
| Sinclair (2017)  [64] | To examine the effects of minimalist, maximalist and conventional footwear on skeletal accelerations and shock attenuation during running. | 10 (M)  **Age**: 24.3 ± 3.5  **Height**: 177.0 ± 11.0  **Weight**: 75.2 ± 6.9 | Healthy  Recreational runners  Run > 3x/week  > 35 km/week  RFS  Wore conventional running footwear  Free of injury | **Accelerometer:** ACL300, Biometrics  **SF:** 1000Hz  **Number**:2  **Location:** Lower back, Tibia (distal anteriomedial aspect, longitudinal axis 0.08 m above the medial malleolus, right) | **Environment:** Indoor (Overground)  **Speed:** Controlled (4 m/s)  **Distance/Time:** 3 (types of footwear) x 5 (right footstrikes)  **Shoe:** Standardised (conventional footwear (New Balance 1260 v2), minimalist (Vibram five-fingers) and maximalist (Hoka One-One)) | 3D Motion Capture (8 camera, Qualisys Track Manager, 250Hz, Visual 3D, C-Motion)  Force plate (Kistler, 1000Hz).  Timing gates (SmartSpeed Ltd) | PTA  Tibial acceleration slope  Peak sacrum acceleration  Shock attenuation **Additional:**  Ankle kinematics | Peak tibial acceleration and shock attenuation were significantly lower in the minimalist in comparison to the conventional and maximalist footwear. Running in conventional and maximalist footwear may place increased demands on the musculoskeletal structures in order to attenuate impact transients, which may be detrimental to passive tissues. |
| Sinclair and Dillon (2016)  [112] | To comparatively examine the effects of energy return, spring and conventional footwear on the kinetics and kinematics of running. | 12 (M)  **Age:** 23.6 ± 2.0  **Height:** 177.1 ± 4.6  **Weight:** 77.5 ± 5.5 | Healthy  Free of injury | **Accelerometer:** ACL300, Biometrics  **SF:** 1000Hz  **Number:** 1  **Location:** Tibia (distal anteriomedial aspect, longitudinal axis 0.08 m above the medial malleolus) | **Environment:** Indoor (Overground)  **Speed:** Controlled (4 m/s)  **Distance/Time:** 3 (types of footwear) x 5 (trials) x right footstrikes  **Shoe:** Standardised | 3D Motion Capture (8 camera, Qualisys Track Manager, 250Hz, Visual 3D, C-Motion)  Force plate (Kistler, 1000Hz).  Timing gates (SmartSpeed Ltd) | PTA  Tibial acceleration slope  Tibial acceleration instantaneous loading rate  **Additional:**  FSA  Ankle kinematics  Peak sacrum acceleration  Shock attenuation, Impact peak  Time to impact peak  AVLR  IVLR | No significant differences in kinetic parameters between footwear. However, it was shown that that spring footwear were associated with significantly greater angles of peak eversion (–12.49°) and tibial internal rotation (13.09°) in comparison to the conventional footwear (eversion = –10.52° & tibial internal rotation = 10.84°). Spring footwear may place runners at increased risk from chronic injury. |
| Sinclair et al. (2014)  [115] | To examine the 3D kinetics and kinematics of footwear designed to promote energy return in relation to conventional running trainers. | 15 (M)  **Age:** 21.0 ±2.0  **Height**: 176.6 ± 5.3  **Weight:** 76.8 ± 6.3 | Healthy  Free fof injury | **Accelerometer:** ACL300, Biometrics  **SF:** 1000Hz  **Number:** 1  **Location:** Tibia (distal anteriomedial aspect, longitudinal axis 0.08 m above the medial malleolus) | **Environment:** Indoor (Overground)  **Speed:** Controlled 4 m/s)  **Distance/Time:** 2 (types of footwear) x 5 (trials) x right footstrikes  **Shoe:** Standardised | 3D Motion Capture (8 camera, Qualisys Track Manager, 250Hz, Visual 3D, C-Motion)  Force plate (Kistler, 1000Hz).  Timing gates (SmartSpeed Ltd) | PTA  Tibial acceleration slope  Tibial acceleration instantaneous loading rate  **Additional:**  FSA  Ankle kinematics  Peak sacrum acceleration  Shock attenuation, Impact peak  Time to impact peak AVLR  IVLR | Tibial accelerations (t(14)=3.26, P<0.05, D=1.74), peak eversion (t(14)=2.75, P<0.05, D=1.47) and tibial internal rotation (t(14)=2.88, P<0.05, D=1.54) were significantly greater in the footwear designed to improve energy return. New footwear may place runners at an increased risk of chronic injury. |
| Sinclair et al. (2015)  [41] | To examine the effects of cooled footwear on the kinetics and kinematics of running in comparison to footwear at normal temperature. | 12 (F)  **Age:** 21.5 ± 3.0  **Height:** 166.0 ± 6.0  **Weight:** 60.9 ± 4.4 | Healthy  Female  University level runners  Free of injury | **Accelerometer:** ACL300, Biometrics  **SF:** 1000Hz  **Weight:** 9g  **Number:** 1  **Location:** Tibia (distal anteriomedial aspect, longitudinal axis 0.08 m above the medial malleolus) | **Environment:** Indoor (Overground)  **Speed:** Controlled (4 m/s0  **Distance/Time:** 2 (footwear temperatures) x 5 (trials) x right footstrikes  **Shoe:** Standardised (Nike Free Run 5.0) | 3D Motion Capture (8 camera, Qualisys Track Manager, 250Hz, Visual 3D, C-Motion)  Force plate (Kistler, 1000Hz).  Timing gates (SmartSpeed Ltd) | PTA  Tibial acceleration slope  Tibial acceleration instantaneous loading rate  **Additional:**  Stance time  FSA  Lower extremity kinematics  Peak sacrum acceleration  Shock attenuation, Midsole deformation impact peak  Time to impact peak  AVLR  IVLR | Instantaneous loading rate (cooled = 186.21 BW/s and normal = 167.08 B W/s), PTA (cooled = 12.75 g and normal = 10.70 g) and tibial acceleration slope (cooled = 478.69 g/s and normal = 327.48 g/s) were significantly greater in the cooled footwear. Peak eversion (cooled = -10.57 ° and normal = -7.83°) and tibial internal rotation (cooled = 10.67 ° and normal = 7.77°) were also shown to be significantly larger in the cooled footwear condition. |
| Sinclair et al. (2017)  [114] | To examine the influence of high and low-cut American football specific footwear on the 3D kinematics and tibial accelerations of three sport specific movements | 12 (M)  **Age:** 22.5 ± 1.1  **Height**:177.0 ± 8.0  **Weight:** 80.3 ± 6.3 | Healthy  Male  Experienced university first team level American football players – offensive positions  Wore low cut footwear  Free of lower extremity injuries | **Accelerometer:** ACL300, Biometrics  **SF:** 1000Hz  **Number:** 1  **Location:** Tibia (distal anteriomedial aspect, longitudinal axis 0.08 m above the medial malleolus) | **Environment:** Indoor (Synthetic grass)  **Speed:** Controlled (4 m/s)  **Distance/Time:** 2 (footwear types) x 5 (trials) x right footstrikes  **Shoe:** Standardised (High (Nike Lunar code pro) and low cut (Nike Vapor pro low TD)) | 3D Motion Capture (8 camera, Qualisys Track Manager, 250Hz, Visual 3D, C-Motion)  Timing gates (SmartSpeed Ltd). | PTA  **Additional:**  FSA  Lower extremity kinematics | Tibial accelerations were significantly greater in the low cut footwear for the run (F(11) = 12:59, p < 0:05, pn2 = 0:53). Peak ankle eversion and tibial internal rotation parameters were significantly greater in the low cut footwear in the running conditions (F(11)= 11:22, p < 0:05, pn2 = 0:48). |
| Stickford et al. (2015)  [139] | To examine whether wearing graduated lower-leg compression sleeves during exercise evokes changes in running economy, perhaps due to altered gait mechanics. | 16 (M)  **Age:** 22.4 ± 3.0  **Height:** 180.6 ± 4.6  **Weight**: 66.4 ± 5.2 | Healthy  Men  College or professional distance runners  Active training  Aged 18-30 years  5000-m time of ≤16:30 Runners who had not raced a 5000-m in the past year were deemed highly trained by VO2max measures (>65 mL/ kg/min) obtained within the past year | **3D Accelerometer:** G-Link, Microstrain  **SF:** 1024Hz  **Accelerometer Range:** 10g  **Number**: 2  **Location:** Shoelaces | **Environment:** Indoor (Treadmill)  **Speed:** Controlled (233, 268, and 300 m/min)  **Distance/Time:** 2 (conditions) x 3 (Speeds) x 30s (minimum of 20 consecutive steps) |  | GCT  SF  SL  FT  Swing Time  Gait variability  Vertical displacement of COM  GRF  Peak displacement of the leg spring  Vertical stiffness  Leg spring stiffness | No differences in GCT, swing time, SF, and SL between control and compression sock trials at any of the running speeds (P = .28–.94). Gait variability was not different from control to compression sock conditions at any of the speeds (P = .42–.73). Runners who improved economy with compression to have lower SF and length variability measures (Cohen d = 0.77–2.24). . GCT was inversely correlated with submaximal VO2. |
| *Pressure Insoles* |  |  |  |  |  |  |  |  |
| Fereydounnia et al. (2022)  [39] | To compare ROM, perceived tightness, and kinetic variables during balance, walking, and running tasks in athletes with and without hamstring tightness. | 30 (F)  **Age:**  Control: 27.27 ± 4.04 Tightness: 29.53 ± 3.40  **Height**: Control: 163.33 ± 4.26  Tightness: 165.20 ± 4.41  **Weight**: Control: 58.93 ± 8.05  Tightness: 60.87 ± 8.14 | Exercised 3x/week for > 2hr  Control: Free of previous injury/tightness to the hamstring muscles  Tightness: ROM tests (popliteal angle of > 30° and higher in the active knee extension test, and straight leg raise < 90°)  Shoe size EU 38  **Exclusion**: History of low back pain, musculoskeletal and neurological disorders in the lower extremities < 1 year  Disease or condition that affected the flexibility of the hamstring muscle | **Pressure Insole:** Moticon SCIENCE  **SF:** 50Hz  **Number:** 2  **Location:** Insole | **Environment:** Indoor (Overground)  **Speed:** Self-selected **Distance/Time:** 15m (Additional slow and fast walking)  **Shoe:** Standardised (Skech-knit Model, Skecher) |  | Mean swing time  Mean total force  Max total force | No variables were significantly different between groups of athletes with and without hamstring tightness |
| Fu et al. (2015)  [53] | To explore the effects of running on different surfaces on the characteristics of in-shoe plantar pressure and tibial acceleration. | 13 (M)  **Age:** 23.7 ± 1.2  **Height**: 173.7 ± 5.7  **Weight**: 65.7 ± 5.2 | Healthy  RFS  Free of musculoskeletal injuries in lower extremity >6 months | **Pressure Insole:** version 4.4, T&T Medilogic Medizintechnik GmbH  **2D Accelerometer:**  Biovision Corp  **SF:** Insole -300Hz  Accelerometer- 1200Hz  **Accelerometer Range:** 50g  **Size:**  Insole: 1.4 × 0.9 × 0.5  Accelerometer 0.6 × 0.4.  **Weight:** Insole 4g  **Number:**  Insole – 2  Accelerometer -1  **Location:** Insole, Tibia (tuberosity, dominant leg) | **Environment:** Indoor (Treadmill and treadmill with EVA cushioning underlay), Outdoor (Concrete, Grass, Track)  **Speed:** Controlled (12 km/hr)  **Distance/Time:** Outdoor: 9 (3 x 3 surfaces) x 10 strides (from 15m zone of a 30m run)  Treadmills: 2 x 10 strides (last min of 3)  **Shoe:** Standardised (Shanghong Shoes Co. Ltd) | High speed camera (MotionPro X-4; Integrated Design Tools Inc, 100hz) | GCT  1st and 2nd PP  PTI  PP distribution  PPA | Among the 5 surfaces, no significant differences were observed in the total GCT, 1st and 2nd PP of the foot, the time to the 1st and 2nd PP, and the PTI during the stance phase. Compared to the concrete surface, running on a cushioned treadmill showed a 12.1% decrease in 1st PP and a 20.8% decrease in PTI during the impact phase. No significant differences in temporal and plantar pressure variables among the 3 over ground surface conditions. No surface effect was observed for the peak plantar pressure at the forefoot, midfoot, medial, and lateral areas. PPA ranged from 10.3 to 12.4 g across different surfaces, however, no significant differences in PPA across the 5 surfaces. |
| García-Pérez et al. (2013)  [143] | Analyse under pre/post fatigue conditions the effect of treadmill vs over ground on plantar pressures | 27 (17M, 10F)  **Age:** 34.0 ± 7.8  **Height:** 173.0 ± 8.0  **Weight:** 66.2 ± 9.4 | Healthy  Recreational Runners | **Pressure Insole:** Biofoot (2001)  **SF:** 500Hz  **Number:** 1  **Location:** Insole (left) | **Environment:** Indoor (Treadmill), Outdoor (Track)  **Speed:** Controlled (3.33, 4.00 m/s)  **Distance/Time:** 2 (speeds) x 2 (surfaces) x 2 (conditions) x 2 stances (left)  **Shoe:** Own |  | GCT  Stride frequency  Stride length  Mean PP  Relative load  **Additional:**  Speed | Running on a treadmill increases CT (7.70% S1 and 9.91% S2), modifies the pressure distribution and reduces PP (25.98% S1 and 31.76% S2), especially under the heel, medial metatarsals, and hallux, compared to running over ground. On both surfaces, fatigue (faster speed) led to a reduced stride frequency (2.78%) and reduced PP on the lateral heel and hallux (15.96% and 16.35%, respectively), and (slower speed) increased relative load on the medial arch (9.53%). No significant interaction between the two factors analysed (surface and fatigue). |
| Hong et al. (2012)  [55] | To compare plantar loads during treadmill running and running on concrete and grass surfaces | 16 (M)  **Age:** 22.9 ± 1.8  **Height:** 170.0 ± 4.7  **Weight:** 63.9 ± 9.7 | Healthy  RFS  University long distance running team  Experienced treadmill or over ground surface  Run > 20 km per week  Free of injury > 6 months  Free of neuromuscular, vestibular and visual systems disease  Right leg dominant  Shoe size of EU41 | **Pressure Insole**: Pedar-X, Novel GmbH  **SF:** 100Hz  **Number**: 2  **Location:** Insole | **Environment:** Indoor (Treadmill), Outdoor (Concrete, Grass)  **Speed:** Controlled (3.8m/s)  **Distance/Time:** 3 (Surfaces) x 5 trials x 5 right foot-strikes  **Shoe:** Standardised (TN600-neutral, ASICS) |  | GCT  Relative GCT  Maximum plantar pressure  Maximum plantar force | Maximum plantar pressures were higher when running on concrete than on natural grass at the total foot and at the central and lateral forefoot. Treadmill running induced a lower maximum pressure at the total foot compared with running on concrete. Compared with over ground running, treadmill running also recorded lower maximum pressures at the great toe and at the lesser toes. Maximum plantar force, there was no difference between running on concrete and on natural grass surfaces. However, compared with over ground running, treadmill running induced a lower maximum plantar force at the total foot, medial forefoot, great toe, and lesser toes. No significant differences were found in the total GCTs of the right foot between surfaces; however, differences were found in specific regions of the foot. |
| Kernozek, Meardon and Vannatta (2014)  [40] | To compare plantar loads between RFS and NRFS after four weeks of running in minimalist footwear | 30 (F)  **Age**: 23.1 ± 1.9  **Height:** 169.0 ± 5.3  **Weight:** 61.7 ± 1.9 | Healthy  Size EU 38 or 41  Interested in making the transition to minimalist footwear  Self-reported running at least 24.1 km/ week  Free of RRI > 6 months | **Pressure Insole**: Pedar X measurement system, Novel GmbH  **SF:** 200Hz  **Number:** 1  **Location:** Insole (right) | **Environment:** Indoor (Treadmill)  **Speed:** Controlled (2.9 m/s or 6.5 mph)  **Distance/Time:** 30 right steps (60s trial)  **Shoe:** Standardised (Vibram Bikila) | - | GCT  FSP  Mean total foot PP  Peak force  Regional PP  PTI  PF  FTI | Differences in plantar loading occurred between FSP running in minimalist footwear. Pressure and force variables were greater in the metatarsals and lower in the heel region in NRFS. PP for the whole foot was greater in NRFS while no difference was observed in maximum force or GCT for the whole foot between strike types. |
| Mei et al. (2015)  [145] | To clarify the function and characteristics of habitually unshod and shod runners under shod and unshod conditions through comparative analysis of spatiotemporal parameters, kinematics, kinetics and plantar pressure. | 38 (M) (19 barefoot, 20 shod)  **Age:**  Barefoot: 23.0 ± 1.2. Shod: 24.0 ± 2.1  **Height:**  Barefoot: 165.0 ± 12.0. Shod: 172.0 ± 16.0  **Weight:**  Barefoot: 65.0 ± 6.9. Shod: 66.0 ± 6.5 | Healthy  Physically active > 3x/week for > 1hr  Right foot dominant  Shoe size EU 41  Free of foot deformities, injuries, lower limb surgery > 6 months  History of amateur running outdoors or on treadmills  No training experiences in running or any other sports | **Pressure Insole:** Novel Pedar Insole System, Novel GmbH  **Number**: 2  **Location**: Insole | **Environment:** Indoor (Overground)  **Speed:** Controlled (3 m/s)  **Distance/Time**: 6 x gait cycles (10m)  **Shoe:** Standardised | 3D Motion Capture (8 camera Vicon system, 200Hz)  High Speed Camera (Fastcam SA 3, 1000Hz)  Force plates (Kistler, 1000Hz) | Plantar Pressure  PP  PTI  Contact area  **Additional:**  GCT  Stride length  Stride Time  FSA  Speed  Lower limb kinematics  GRF (PP, Contact area  PTI, AVLR) | Habitually shod runners exhibited a decreased FSA under unshod conditions; and the vertical average loading rate of shod runners under unshod conditions was larger than that under shod conditions. Under shod conditions, the separate hallux of habitually unshod runners and other toes of habitually shod runners show greater PP and PTI with reduced value compared to the medial forefoot and lateral forefoot while comparing with running with shoes. FSP is more important than the shod or unshod running style. |
| Mei, Graham and Gu (2014)  [146] | To probe into the difference in plantar pressure and dorsal pressure of three types of sports shoes: Basketball Shoes (BS); Running Shoes (RS); Tennis Shoes (TS). | 9 (M)  **Age**: 24.0 ± 2.1  **Height:** 171.0 ± 4.96  **Weight**: 63.0 ± 6.78 | Healthy  Recreational runners  Free of injury  Experience of treadmill running  Right foot dominant  Shoe size EU 41 | **Pressure Insole**: Novel Pedar Insole System, Novel GmbH  **SF:** 100Hz  **Number:** 2  **Location:** Insole | **Environment:** Indoor (Treadmill)  **Speed:** Controlled (8 km/hr)  **Distance/Time:** 3x 5 steps  **Shoe:** Standardised (basketball, tennis, running) |  | Distribution of plantar pressure in different zones | No significant differences in anterior-posterior or medial-lateral max. pressure and mean pressure of 4 zones with 3 shoes nor upper max. and mean pressure of 3 zones (p > 0.05). Pressure of lateral fifth metatarsophalangeal joint is distinctly different, with maximal pressure (p < 0.05) and mean pressure (p < 0.05). Maximal Lateral metatarsophalangeal joint of tennis is distinctively different from basketball and running shoes, mean metatarsophalangeal of running shoes is apparently distinct compared to other shoes. |
| Musgjerd et al. (2021)  [147] | To examine effects of increased cadence on peak impact force during running in an outdoor setting. | 15 (7M, 8F)  **Age**: Average 23.5 years (range 22-26) | Healthy  Recreational runners  **Exclusion:** Lower extremity injury <3 months  History or current of lower extremity surgery or pain during running  Medical pathology that would cause difficulty running for up to 1hr | **Pressure Insole**: Loadsol, Novel GmbH  **SF:** 100Hz  **Number:** 2  **Location:** Insole | **Environment:** Outdoor (Road)  **Speed:** Self-selected (comfortable)  **Gradient:** Flat  **Distance/Time:** 2 (sessions) x 2.4 mile (20 right foot steps surrounding each quarter-mile increment)  **Shoe:** Own | GPS watch (measuring SF, Garmin Forerunner 25) | SF  Peak Force | Cadence differences of 7.3% were observed between baseline and cadence sessions (p<0.001). A concurrent decrease in average peak force of 5.6% was demonstrated during the cadence run (p<0.05). Average cadences measured by GPS watch and insoles were found to be the same at both baseline (p=0.096) and during cadence (p=0.352) sessions. |
| Orendurff et al. (2008)  [148] | To compare the PPs at seven anatomical regions of the foot during running straight, accelerating, cutting, jump take-off, and jump landing with two different shoe types | 10 (M)  **Age:** 20.5 ± 1.0  **Height:** 186.8 ±5.3  **Weight**: 102.3 ±18.9 | Healthy  Collegiate runners, footballers, soccer players  Free from musculoskeletal injury requiring medical attention for at least 2 years by self-report  Never had foot or ankle injury requiring surgery or casting | **Pressure Insole**: Pedar Mobile, Novel GmbH  **SF:** 99Hz  **Number:** 2  **Location:** Insole | **Environment:** Indoor (Overground)  **Speed:** Self-selected (75% max. speed)  **Distance/Time:** 2 x 9m  **Shoe:** Standardised (Turf shoe (Air Pro Turf Low, Nike, Beaverton, Ore) and a cleat (Speed TD, Nike) | Digital Video Camera (30Hz) | PP | Running straight ahead had the highest pressure at the great toe, with a general decline in PP from the first metatarsal head to the central forefoot, fifth metatarsal head, medial midfoot, lateral midfoot, and heel. During straight running, the heel showed low PPs compared with cutting left, cutting right, jump take-off, and jump landing; only acceleration had lower peak heel pressures. PP at the heel showed a high degree of variability and no statistically significant differences between shoes. However, significant reductions in PP were observed in the Air Pro Turf compared with the Speed TD at the first metatarsal head (42.6 ±12.1N/cm2 vs 51.7 ±10.8 N/cm2; P<.0001). |
| Queen et al. (2010)  [37] | Determine differences in plantar pressure between genders when running in training shoes and racing flats | 34 (17M, 17F)  **Age:**  M: 23.1 ± 3.4  F: 22.9 ± 2.8  **Height:**  M: 175.0 ± 4.0  F: 166.0 ± 5.0  **Weight:**  M: 69.2 ± 6.8  F: 57.7 ± 5.6 | Healthy  Running > 10 miles/week  Free of lower extremity injuries > 6 months  No history of foot or ankle surgery > 3 years  No history of metatarsal stress fractures | **Pressure Insole:** Pedar-X, Novel GmbH  **Number:** 2  **Location:** Insole | **Environment:** Indoor (Overground)  **Speed:** Self-selected  **Distance/Time:** 2 (shoes) x 7 (trials) x 4 (footsrikes, 2L, 2R)  **Shoe:** Standardised (Nike Air Zoom Katana IV (racing flat), Nike Air Pegasus (training shoe)) | - | GCT  Maximum force  Contact area | A significant main effect existed for total foot maximum force according to shoe type (p<0.0001), total foot contact area according to shoe type (p= 0.029), gender (p= 0.05) with women having the greatest contact area (0.932 NICA vs. 0.902 NICA). No significant main effects or interaction for GCT. Based on the gender differences in loading, running shoe design should be gender specific in an attempt to prevent injuries. |
| Roca-Dols et al. (2018)  [71] | To know how five different cushioning shoes (Boost®, Ethyl-vinyl-acetate (EVA), Air® chamber, pronation-control cushioning technologies and minimalist) may interfere in GCT of each gait phase of walking and running in contrast with barefoot condition. | 30 (M)  **Age**: 36.2 ± 8.5  **Height:** 177.2 ± 4.3  **Weight:** 72.1 ± 6.4 | Healthy  Recreational runners  Wear neutral sports shoes  Neutral FSP or RFS  Normal dorsiflexion in ankle joint complex with at least 10° with the knee fully dorsiflexed  Normal ROM in subtalar joint of 30°  Normal ROM in non-weight-bearing in the first metatarsocuneiform joint with 4mm in dorsiflexion and 4mm in plantarflexión of first metatarsal bone  Normal unrestricted motion along the longitudinal axis of the midtarsal joint of 15°  ROM > 82° in first MPJ mobility according to Buell technique | **Pressure Insole:** SX230  **Size:** 1.27  **Number**: 6  **Location:** Insole | **Environment:** Indoor (Treadmill)  **Speed:** Controlled (9km/hr)  **Distance/Time**: 30 x 30s (5 trials x 6 conditions x 30s (last 30s of 3 min trials)). (Additionally: 30 x 30s for walking condition at 5km/hr)  **Shoe:** Standardised (barefoot, minimalist, Boost®, Air® chamber, EVA and pronation control) | - | Pre-activation phase  Heel contact phase  Midstance phase  Propulsion phase | Barefoot condition provided the shortest GCT. Cushioning running shoes, and even minimalist, induced higher GCT in PP during running than barefoot condition. Air® chamber sport shoe was the model that spent significantly more time in contact with the ground in all three analysed dynamic phases and showed the highest GCT during PP versus barefoot. |
| Sun et al. (2018)  [65] | To explore the effects of strike patterns and shoe conditions on foot loading during running. | 12 (M)  **Age:** 21.0 ± 1.3  **Height:** 177.1 ± 4.2  **Weight**: 68.1 ± 7.4 | Healthy  Recreational runners  3-4 years of running experience  RFS  Free of lower extremity injury > 6 months  No vigorous exercise within 24 hours | **Pressure Insole:** T&T Medilogic Medizintechnik GmbH  **SF:** 120Hz  **Size:** 0.6 × 0.4 x 0.12  **Number**: 2  **Location**: Insole | **Environment:** Indoor (Overground)  **Speed**: Controlled (3 m/s)  **Distance/Time**: 2 (FSP) x 2 (shoe conditions) x 3 (trials/footstrikes)  **Shoe:** Standardised | Force plates (2 x 9287B, Kistler Corporation, 1200Hz). Timing gates (Witty-Manual, Microgate) | PP  PTI  **Additional:**  GCT  vGRF  Peak vertical loading rate | FSP (15 of the 18 variables) had a more significant effect on the plantar pressure characteristics than shod conditions (7 of the 18 variables). FSP had a significant effect on the plantar pressure at all plantar regions, time to peak plantar pressure at four plantar regions, the PTI at the five plantar regions. Average PP and average PTI of the entire foot were significantly reduced with FFS compared to RFS. Moreover, the lowest PP and PTI were obtained during shod running with FFS. |
| Tao et al. (2019)  [125] | To compare the spatiotemporal factors between over ground and treadmill running in collegiate runners, using pressure sensor insoles | 21 (4M, 17F)  **Age:** 20.1 ± 1.5  **BMI:** 20.1 ± 1.6 | Healthy  USD Division I Men’s and Women’s Cross-Country team  **Exclusion:** Injury or illness | **Pressure Insole:** Moticon  **SF:** 50Hz  **Number:** 2  **Location:** Insole | **Environment:** Indoor (Treadmill, Track)  **Speed:** Self-selected; (Easy run pace)  **Gradient:** 1%  **Distance/Time:** 3 (Overground, flat and 1%) x 6s (trials =60s)  **Shoe:** Own | Timing (PowerMax Speed Timer) | SF  Stance time  Swing time  Center of pressure progression  Foot pressure distributions  Rate and magnitude of force development | Compared to over ground running, level and incline treadmill running was associated with increased SF (mean difference [MD]=3.55-3.22 strides per minute; p< 0.01), decreased stance duration (MD=14-16 ms; p< 0.01), and decreased swing duration (MD=11-12 ms; p< 0.05). |
| Tessuti et al. (2008)  [151] | To investigate how loads are distributed over the plantar surface while running on natural grass and on a rigid surface—asphalt | 44 (32M, 12F)  **Age:** 35.7 ± 6.8  **Height:**  M:177.0 ± 6.0  F:163.0 ± 5.0  **Weight:**  M:75.5 ± 10.6  F:58.1 ± 4.0 | Healthy  Recreational runners  Experience running on natural grass and asphalt  Run > 20 km weekly  Free of musculoskeletal injury > 6 months  Leg length discrepancy < 1cm | **Pressure Insole**: Pedar-X, Novel GmbH  **SF:** 100Hz  **Number**: 2  **Location:** Insole | **Environment:** Outdoor (Grass, Asphalt)  **Speed:** Controlled (12 km/hr)  **Distance/Time:** 2 (surfaces) x 40m  **Shoe:** Standardised (Rainha System, Rainha) |  | GCT  PP  Contact area | Asphalt and natural grass were statistically different in all variables. Higher peak pressures were observed on asphalt at the central (p < 0.001) [grass: 303.8(66.7) kPa; asphalt: 342.3(76.3) kPa] and lateral rearfoot (p < 0.001) [grass: 312.7(75.8) kPa; asphalt: 350.9(98.3) kPa] and lateral forefoot (p < 0.001) [grass: 221.5(42.9) kPa; asphalt: 245.3(55.5) kPa]. For natural grass, GCT and contact area were significantly greater at the central rearfoot (p < 0.001). Natural grass may be a surface that provokes lighter loads on the rearfoot and forefoot in recreational runners. |
| Tessuti et al. (2012)  [66] | To investigate the influence of running on asphalt, concrete, natural grass, and rubber on in-shoe pressure patterns in adult recreational runners | 47 (34M, 13F)  **Age:**  M: 35.1  F: 38.9  **Height:**  M:178.0 ± 6.0  F:159.0 ± 5.0  **Weight:**  M:73.5 ± 10.6  F:53.2 ± 4.0 | Healthy  Recreational runners  Aged 18-50 years  Experience running on natural grass and asphalt  Run > 20 km weekly  Free of musculoskeletal injury > 6 months  Leg length discrepancy < 1cm | **Pressure Insole:** Pedar-X, Novel GmbH  **SF:** 100Hz  **Size:** 2.55  **Number:** 2  **Location:** Insole | **Environment:** Outdoor (Concrete, Grass, Track, Asphalt)  **Speed:** Controlled (12 km/hr)  **Distance/Time:** 4 (surfaces) x 2 (trials) x 40m  **Shoe:** Standardised (Rainha System, Rainha) |  | GCT  PP  PTI | Asphalt and concrete were similar for all plantar variables and pressure zones. Running on grass produced peak pressures 9.3% to 16.6% lower (P< 0.001) than the other surfaces in the rearfoot and 4.7% to 12.3% (P<0.05) lower in the forefoot. The GCT on rubber was greater than on concrete for the rearfoot and midfoot. Running on natural grass attenuates in-shoe plantar pressures in recreational runners. |
| Wang et al. (2012)  [67] | To compare plantar loads during running on different over ground surfaces | 15 (M)  **Age:** 22.0 ± 1.8  **Height:** 170.4 ± 3.3  **Weight:** 62.8 ± 9.5 | Healthy  Male  University long distance running team  RFS  Experienced treadmill or over ground surface  Run > 20 km/week  Free of lower limb musculoskeletal injury or other medical problems >6 months  Shoe size EY41  Right leg dominant | **Pressure Insole**: Pedar-X, Novel GmbH  **SF:** 100Hz  **Number**: 2  **Location**: Insole | **Environment:** Outdoor (Concrete, Grass, Track)  **Speed:** Controlled (3.8m/s)  **Distance/Time:** 3 (Surfaces) x 5 trials x 5 right foot-strikes  **Shoe:** Standardised (TN600-neutral, ASICS) |  | GCT  Relative GCT  Maximum plantar pressure  Maximum plantar force | Compared with running on concrete surface, running on natural grass showed a lower magnitude of maximum plantar pressure at the total foot (451.8kPa vs. 401.7kPa, p = 0.016), lateral midfoot (175.3kPa vs. 148.0kPa, p = 0.004), central forefoot (366.3kPa vs. 336.8kPa, p = 0.003), and lateral forefoot (290.2kPa vs. 257.9kPa, p = 0.004). Running on natural grass showed a longer relative GCT compared with running on a concrete surface at the central forefoot (81.9% vs. 78.8%, p = 0.017) and lateral forefoot (75.2% vs. 73.1%, p = 0.007). |
| Wei et al. (2020)  [69] | To compare the plantar loads between habitual RFS and NRFS during running under the participant’s preferred speed | 66 (M)  **Age:**  RFS = 24.0 ± 2.6  NRFS = 26.4 ± 4.4  **Height:**  RFS = 171.7 ± 5.7  NRFS = 173.1 ± 4.0  **Weight:**  RFS = 67.8 ± 10.6  NRFS = 68.9 ± 9.6 | Healthy  Male amateur distance runners  Free of lower limb musculoskeletal injury or other medical problems >6 months  Right leg dominant | **Pressure Insole:** Pedar-X, Novel GmbH  **SF:** 100Hx  **Number:** 2  **Location:** Insole | **Environment:** Indoor (Track)  **Speed:** Self-selected  **Distance/Time:** 3 (right foot strikes) (3m testing area within a 15m runway)  **Shoe:** Standardised (Asics, Sortiemagic RP 4 TMM467-0790) | High-speed video camera (Motion Pro X-4, 100Hz)  Photoelectric timing system (Wittysem, Microgate) | Strike index  PP  PTI  Contact area | Running speed has a significant effect on the total contact area [F (1, 64) = 7.061, P= 0.01, n2 = 0.101], which also affects midfoot and forefoot regions. No significant difference was found on the total maximum force, force-time-integral, PP and pressure time- integral, but the total contact area of RFS was higher than that of NRFS runners [F (1, 64) = 77.406, P < 0.001, n2 = 0.551]. Plantar loads were mainly focused on the heel and midfoot for RFS runners in all variables, and NRFS runners experienced increased PP and PTI in medial forefoot regions. |
| Wei et al. (2019)  [68] | To explore the plantar loading variables between habitual RFS and NRFS during running | 78 (M)  **Age:**  RFS = 24.2 ± 2.5.  FFS = 27.9 ± 6.2  **Height:**  RFS = 171.8 ± 5.7  FFS = 173.0 ± 4.2  **Weight:**  RFS = 68.4 ± 10.2  FFS = 68.3 ± 9.4 | Healthy  Male amateur distance runners  Free of lower limb musculoskeletal injury or other medical problems >6 months  Right leg dominant  **Exclusion:** Musculoskeletal, visual system, vestibular and/or other medical conditions in the previous six months | **Pressure Insole**: Pedar-X, Novel GmbH  **SF:** 100Hz  **Number**: 2  **Location:** Insole | **Environment:** Indoor (Track)  **Speed:** Controlled (12.0 ± 5% km/hr)  **Distance/Time:** 3 (right foot strikes) (3m testing area within a 15m runway)  **Shoe:** Standardised (Asics, Sortiemagic RP 4 TMM467-0790) | High-speed video camera (Motion Pro X-4, 100Hz)  Photoelectric timing system (Wittysem, Microgate) | Strike index  PP  PTI  Contact area | No difference was found for the total foot in PP or PTI between different FSPs. Force and pressure parameters were much higher in the rearfoot and midfoot regions during RFS running and relatively greater in forefoot region during NRFS running. However, compared with NRFS running, the contact area, maximum force and force-time-integrals during RFS running on total foot were 21.44% (P < 0.001, ES = 2.29), 13.99% (P = 0.006, ES = 0.64) and 21.27% (P < 0.001, ES = 0.85) higher, respectively. Total foot PP and pressure-time-integral between two FSPs were similar. |
